# Supplementary material for: CDK4/6 inhibition uncovers subtype-specific vulnerabilities and immune-related responses in esophageal squamous cell carcinoma
Source: Cell Death Dis. 2026 May 29;17(1):666. doi: 10.1038/s41419-026-08892-x (PMC13424112; doi:10.1038/s41419-026-08892-x)

## Supplemental Data

## Supplementary Figures

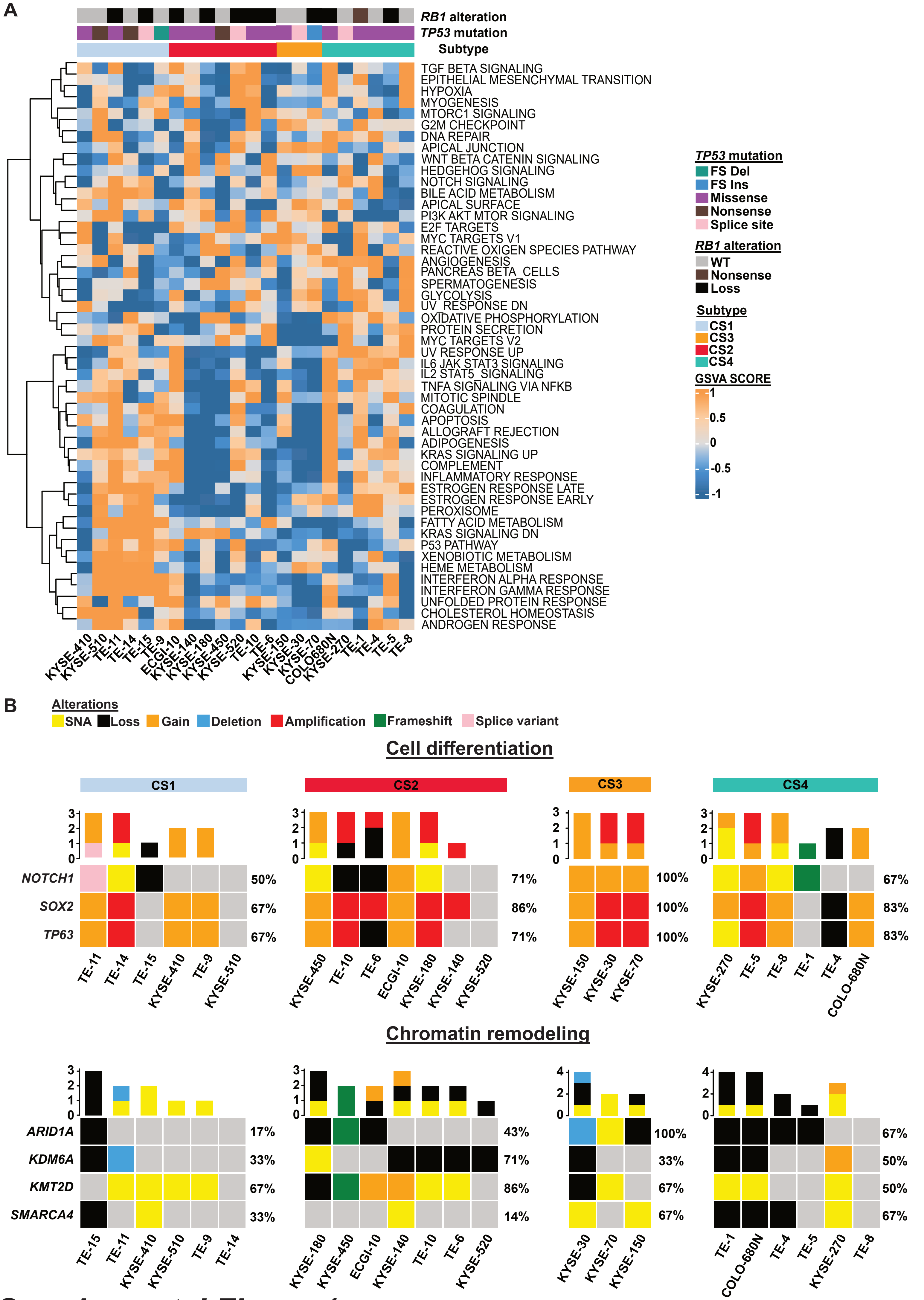


# Supplemental Figure 1

**Supplementary Figure S1. Mutational background of eSCC cell lines**

**(A)** Gene Set Variation Analysis (GSVA) of Hallmark gene sets across 22 eSCC cell lines. Heatmap displays pathway activity scores, highlighting heterogeneity in signaling programs relevant to proliferation, differentiation, metabolism, and immune response. Cell lines are grouped according to the MOVICS-derived clusters (CS1–CS4). **(B)** OncoPrint visualization showing genomic alterations in genes associated with squamous cell differentiation and chromatin remodeling complexes. The plot displays amplifications, deletions, gains, losses, and point mutations across the 22 eSCC cell lines, grouped by the MOVICS-derived clusters (CS1–CS4). Percentages indicate the proportion of altered cell lines per gene within each cluster.

A

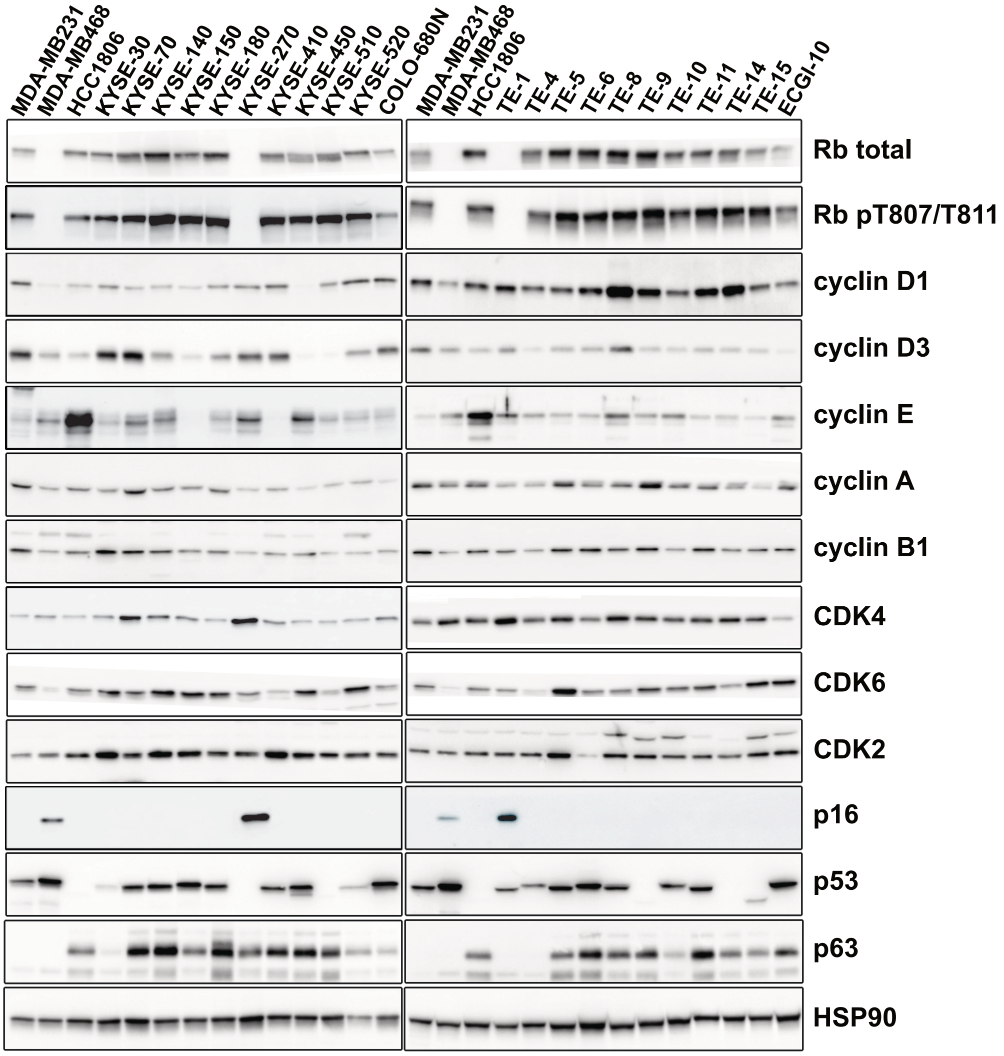


B

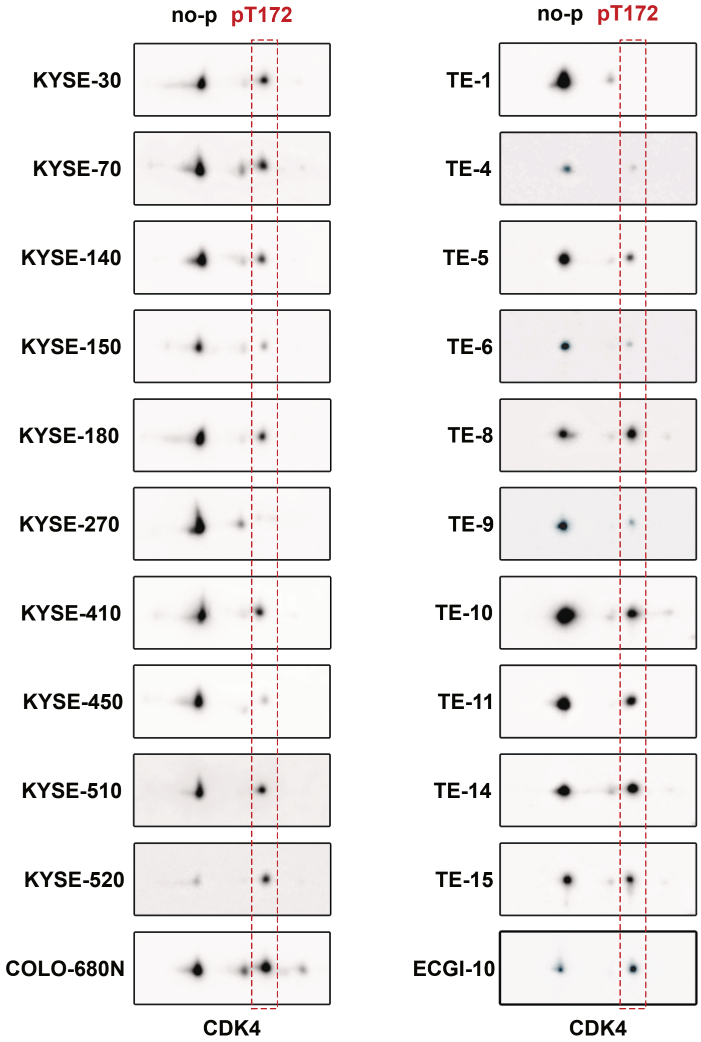


*Supplemental Figure 2*

**Supplementary Figure S2. Mutational background of eSCC cell lines**

**(A)** Western blot analysis of key components of the cyclin D-CDK4/6–Rb pathway across 22 eSCC cell lines in proliferative untreated condition. Protein lysates from three breast cancer cell lines (MDA-MB-231, MDA-MB-468, and HCC1806) were included as reference controls. **(B)** 2D Western blotting of total CDK4 and its activating phosphorylation at threonine 172 (pT172) in representative untreated eSCC cell line.

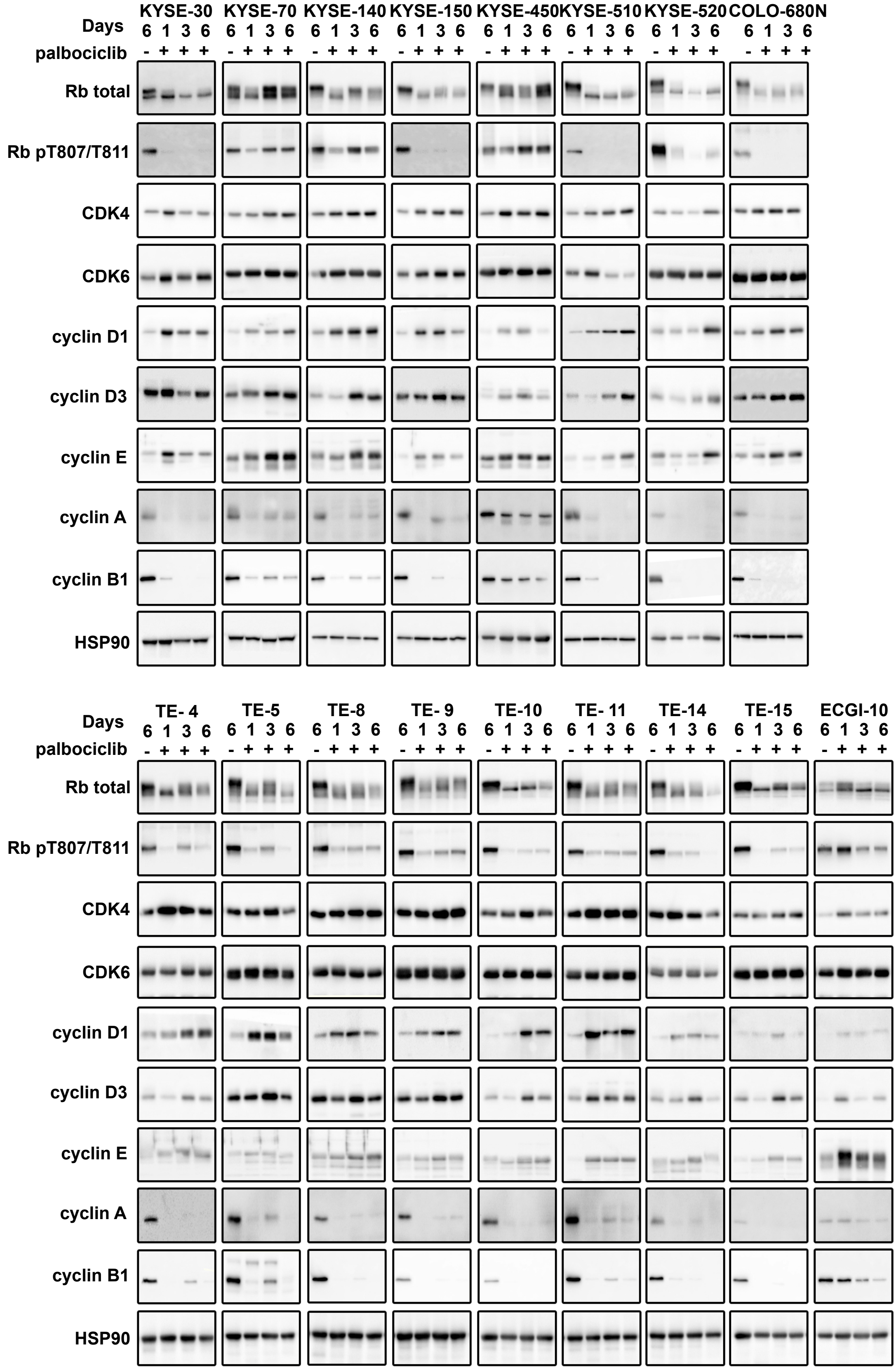


*Supplemental Figure 3*

**Supplementary Figure S3. Protein expression dynamics following 6 days of Palbociclib treatment (extended panel).**

Western blot analysis on whole cell lysates collected after 6 days of continuous 1 μM palbociclib or DMSO treatment in 17 eSCC cell lines. The panel includes phosphorylated Retinoblastoma protein (Rb pS807/811), total Rb, CDK4, CDK6, cyclin D1, cyclin D3, cyclin A, cyclin E, and cyclin B1. HSP90 was used as a loading control. TE-1, KYSE270, TE-6, KYSE-180 and KYSE-410 cell lines are shown in the Figure 3 panel (A-B) as representative cell lines.

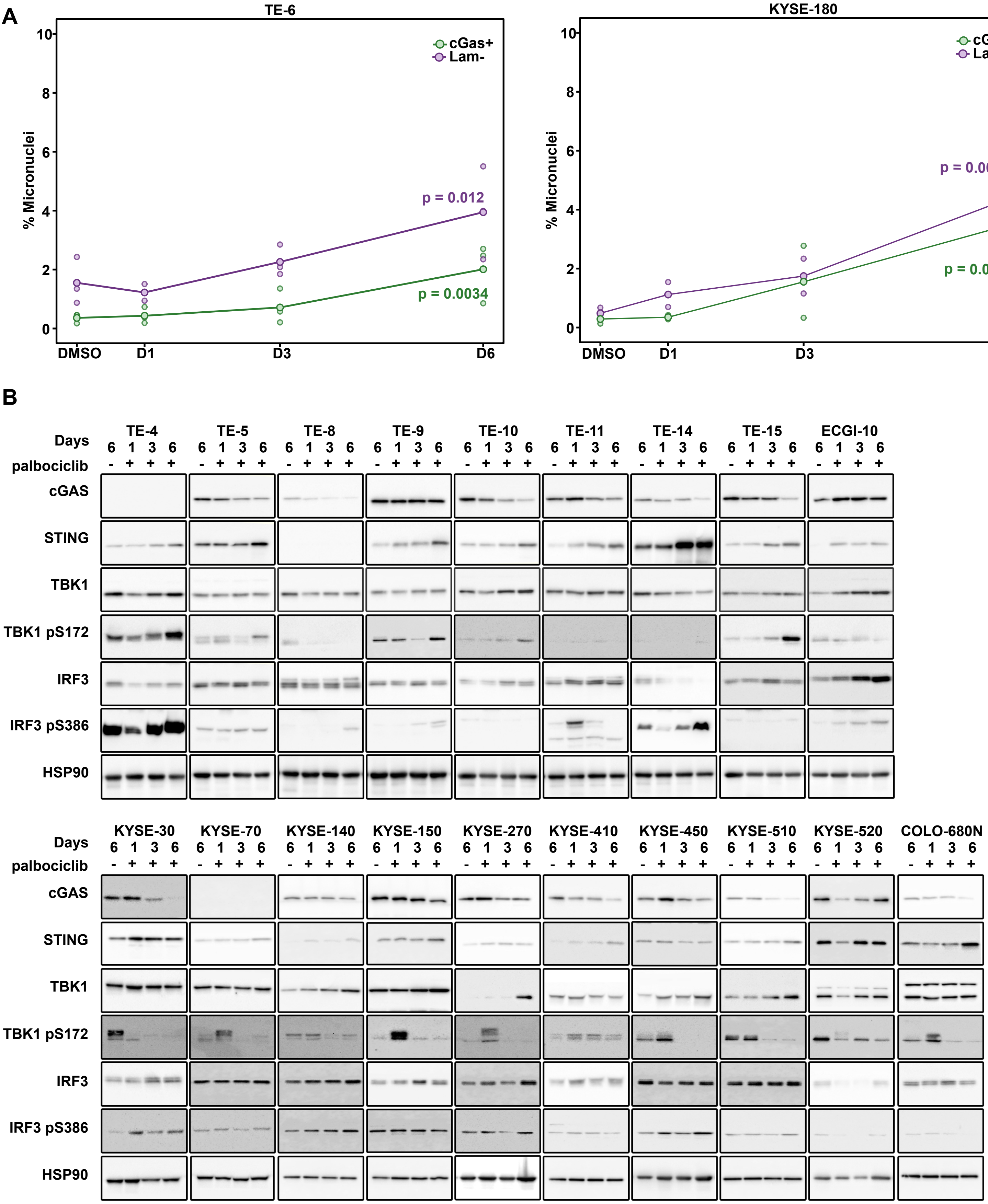


*Supplemental Figure 4*

**Supplementary Figure S4. Extended analysis of micronuclei composition and cGAS–STING pathway activation across eSCC cell lines.**

**(A)** Quantification of micronuclei status over time in two eSCC cell lines (TE-6 and KYSE-180) treated with 1 μM palbociclib or DMSO. Micronuclei were classified as cGAS-positive ($cGAS^{+}$), Lamin A/C-negative ($Lamin^{-}$). Shown are the percentages of $cGAS^{+}$ and $Lamin^{-}$ micronuclei across days 1, 3, and 6. **(B)** Western blot analysis of cGAS–STING pathway activation across the full panel of 19 eSCC cell lines treated with 1 μM palbociclib or DMSO for 1, 3, or 6 days. TE-1, TE-6, and KYSE-180 cell lines are shown in Figure 4. HSP90 was used as a loading control.

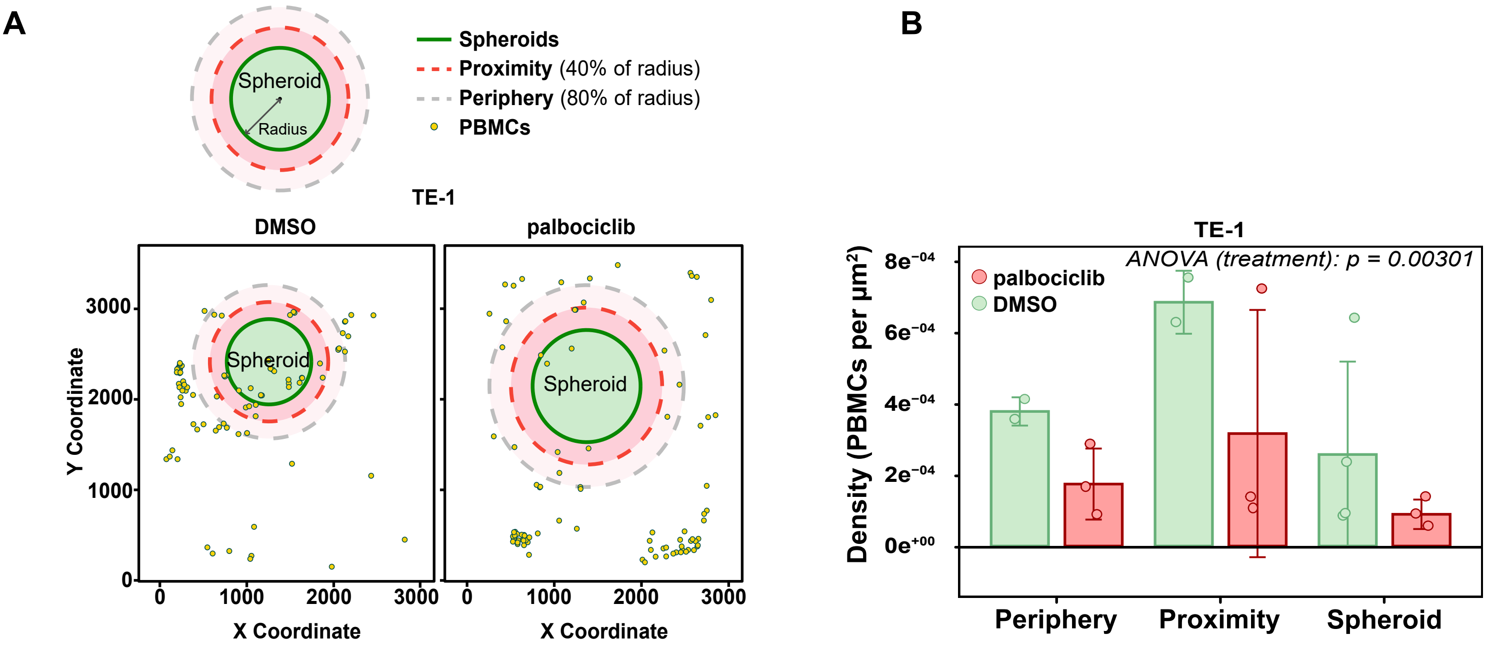


*Suppl.Figure 5*

**Supplementary Figure S5. Elevated basal immune cell infiltration in a palbociclib-resistant eSCC microfluidic model.**

**(A)** Computational mapping of PBMCs spatial distribution in the TE-1 (palbociclib-resistant) OrganiX device after PBMCs co-culture. PBMC positions (cyan dots) are plotted relative to the spheroid center for 1 µM palbociclib (left) and DMSO (right) conditions. Coordinates were extracted from QuPath segmentations of the immunofluorescence images and processed in R. Red and gray dashed circles indicate the spheroid, proximity, and periphery regions used for density quantification. **(B)** Quantification of PBMC spatial density across three concentric radial regions: spheroid, proximity, and periphery. Density was calculated as the PBMC area normalized to the surface area of each region. Statistical significance was assessed using aligned rank transform (ART) ANOVA; p-values for treatment effects are indicated.

## Supplementary Table Legends

**Supplementary Table S1**

Table reporting the information about the used eSCC cell lines and their culturing medium and conditions.

| Cell Line | MORPHOLOGY | MEDIUM | PATIENT SEX | PATIENT AGE | LOCATION | Pre-Treatment? |
|---|---|---|---|---|---|---|
| **COLO-680N** | Adherent, epitheloid cells growing in monolayers | 90% RPMI + 10% FBS + 1% PenStrep | FEMALE | 57 | NA | NO |
| **KYSE-30** | Adherent, epitheloid cells growing in monolayers | 45% RPMI + 45% Ham's F12 + 5-10% FBS + 1% PenStrep | MALE | 64 | middle intra-thoracic esophagus | NO |
| **KYSE-70** | Adherent, epitheloid cells growing in monolayers | 90% RPMI + 10% FBS + 1% PenStrep | MALE | 77 | Lower thoracic esophagus | NO |
| **KYSE-140** | Adherent cells growing as monolayer with the tendency to pile up | 90% RPMI + 10% FBS + 1% PenStrep | MALE | 54 | middle intra-thoracic esophagus | NO |
| **KYSE-150** | Adherent, epitheloid cells growing in monolayers | 49% RPMI + 49% Ham's F12 + 2% FBS + 1% PenStrep | FEMALE | 49 | cervical esophagus | Radiotherapy |
| **KYSE-180** | Adherent, epitheloid cells growing in monolayers | 90% RPMI + 10% FBS + 1% PenStrep | MALE | 53 | Lower thoracic esophagus | NO |
| **KYSE-270** | Adherent, epitheloid cells growing in monolayers | 49% RPMI 1640 + 49% Ham's F12 + 2% FBS + 1% PenStrep | MALE | 79 | middle intra-thoracic esophagus | NO |
| **KYSE-410** | Adherent, epitheloid cells growing in monolayers | 90% RPMI + 10% FBS + 1% PenStrep | MALE | 51 | cervical esophagus | NO |
| **KYSE-450** | Adherent, epitheloid cells growing in monolayers | 45% RPMI + 45% Ham's F12 + 10% FBS + 1% PenStrep | MALE | 59 | Lower thoracic esophagus | NO |

| | | | | | | |
|---|---|---|---|---|---|---|
| **KYSE-510** | Adherent, epitheloid cells growing as monolayer with multilayer foci | 90% RPMI + 10% FBS + 1% PenStrep | FEMALE | 67 | cervical esophagus | Cisplatin and Radiotherapy |
| **KYSE-520** | Adherent, epitheloid cells growing as monolayer, occasional giant cells present (ca. 1%) | 90% RPMI + 10% FBS + 1% PenStrep | FEMALE | 58 | lower intra-thoracic esophagus | NO |
| **ECGI-10** | Adherent, epitheloid cells growing as monolayer | Ham's F12 90% + 10% FBS + 1%PenStrep | MALE | 65 | NA | NO |
| **TE-1** | Adherent, epitheloid cells growing as monolayer | 90% RPMI + 10% FBS + 1% PenStrep | MALE | NA | Lower thoracic esophagus | NO |
| **TE-4** | Adherent, epitheloid cells growing as monolayer | 90% RPMI + 10% FBS + 1% PenStrep | FEMALE | NA | Middle thoracic esophagus | NO |
| **TE-5** | Adherent, epitheloid cells growing as monolayer | 90% RPMI + 10% FBS + 1% PenStrep | FEMALE | NA | upper thoracic esophagus | NO |
| **TE-6** | Adherent, epitheloid cells growing as monolayer with the tendency to pile up | 90% RPMI + 10% FBS + 1% PenStrep | MALE | NA | Lower thoracic esophagus | NO |
| **TE-8** | Adherent, epitheloid cells growing as monolayer | 90% RPMI + 10% FBS + 1% PenStrep | MALE | NA | Lower thoracic esophagus | NO |
| **TE-9** | Adherent, epitheloid cells growing as monolayer | 90% RPMI + 10% FBS + 1% PenStrep | MALE | NA | Pleural effusion | NO |
| **TE-10** | Adherent, epitheloid cells growing as monolayer | 90% RPMI + 10% FBS + 1% PenStrep | MALE | NA | upper thoracic esophagus | NO |

| | | | | | | |
|---|---|---|---|---|---|---|
| **TE-11** | Adherent, epitheloid cells growing as monolayer | 90% RPMI + 10% FBS + 1% PenStrep | MALE | 58 | Lower thoracic esophagus | NO |
| **TE-14** | Adherent, epitheloid cells growing as monolayer with the tendency to pile up | 90% RPMI + 10% FBS + 1% PenStrep | MALE | 57 | upper thoracic esophagus | NO |
| **TE-15** | Adherent, epitheloid cells growing as monolayer with the tendency to pile up | 90% RPMI + 10% FBS + 1% PenStrep | FEMALE | 58 | Middle thoracic esophagus | NO |

**Supplementary Table S2**

List of the used antibodies and their information

**Supplementary Table S3**

Matrices for 1° RNA sequencing reads before normalization and filtering for all 22 cell lines in both conditions (DMSO or PD palbociclib 1µM) and each biological replicate _1/_2 , 2° differentially regulated genes (DEG) for each cell lines based on the comparison PD vs DMSO CTL condition, 3° Gene set enrichment scores for each cell line comparison based on DEGs

| Target | Source | Company | Catalog No. |
| --- | --- | --- | --- |
| **Primary antibody** | | | |
| HSP90 | Mouse | BD | 610419 |
| Rb | Mouse | BD | 554136 |
| p-RB | Rabbit | Cell Signaling Technology | 8516 |
| CDK4 | Rabbit | Cell Signaling Technology | 12790 |
| CDK6 | Rabbit | Santa Cruz | 177 |
| Cyclin D1 | Mouse | Abcam | 187364 |
| Cyclin D3 | Mouse | Santa Cruz | 56308 |
| Cyclin E | Mouse | Invitrogen | MA5-14336 |
| Cyclin A | Mouse | Santa Cruz | 53232 |
| Cyclin B1 | Mouse | Santa Cruz | 245 |
| CDK2 | Rabbit | Santa Cruz Biotechnology | 6248 |
| p16 CDKN2A | Mouse | Abcam | 117443 |
| p53 | Mouse | Santa Cruz Biotechnology | 126 |
| TP63 | Rabbit | Abcam | 124762 |
| cGAS | Mouse | Cell Signaling Technology | 15102S |
| STING | Rabbit | Cell Signaling Technology | 13647 |
| TBK1/NAK | Rabbit | Cell Signaling Technology | 3504 |
| IRF3 | Rabbit | Cell Signaling Technology | 4302 |
| IRF3 phospho S396 | Rabbit | Cell Signaling Technology | 4947 |
| pIRF3 (Ser396) | Rabbit | Cell Signaling Technology | 29047 |
| pIRF3 (Ser386) | Rabbit | Cell Signaling Technology | 37829 |
| phospho-TBK1/NAK (S172) | Rabbit | Cell Signaling Technology | 5483 |
| BrdU | Mouse | Bio-Rad | MCA6144 |
| Lamin A/C | Mouse | Santa Cruz | 376248 |
| Phospho-Histone H2A.X (Ser139) | Rabbit | Cell Signaling Technology | 9718 |
| CD45 (YAML501.4) | Rat | Invitrogen | MA5-17687 |
| CD31 | Mouse | BD Pharmingen | 555444 |
| **Secondary antibody** | | | |
| anti-rabbit Alexa Fluor 488 | Donkey | Jackson ImmunoResearch | 711-545-152 |
| anti-mouse Alexa Fluor 488 | Goat | Jackson ImmunoResearch | 115-545-003 |
| anti-mouse Alexa Fluor 647 | Goat | Jackson ImmunoResearch | 115-357-062 |
| Anti-mouse IgG, HRP-linked Antibody | Horse | Cell Signaling Technology | 7076 |
| Anti-rabbit IgG, HRP-linked Antibody | Goat | Cell Signaling Technology | 7074 |
| Anti-Rat IgG (H+L), Alexa Fluor 555 | Goat | ThermoFischer scientific | A48263 |
| Anti-Mouse IgG1,Alexa Fluor 647 | Goat | ThermoFischer scientific | A-21240 |

## Supplementary Original Western Blot Images

# Figure 3 & supplementary Figure 3

Exposure 6 + marker

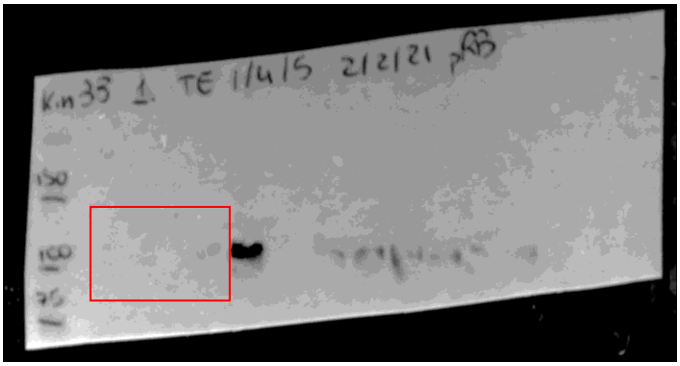

pRb

TE-1

**Exposure 8**

**Cyclin E**

**50 KDa**

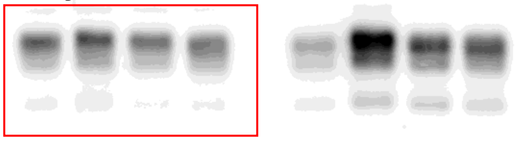

**TE-1**

Exposure 6 + marker

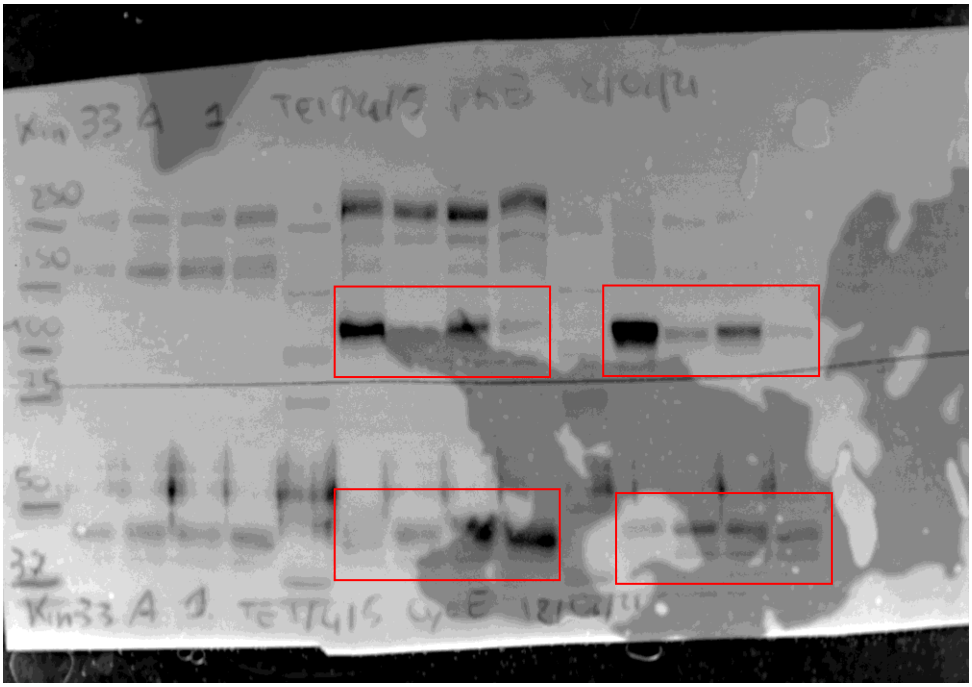

**pRb**

**Cyclin E**

**TE-4** **TE-5**

Exposure 7 + marker

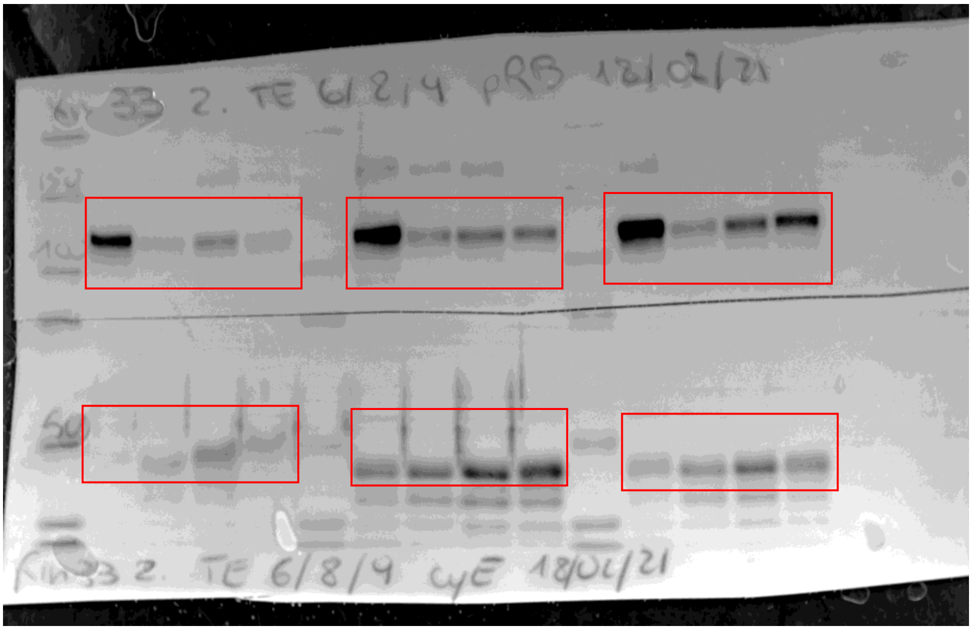

**pRb**

**Cyclin E**

**TE-6** **TE-8** **TE-9**

# Figure 3 & supplementary Figure 3

**Exposure 7 + marker**

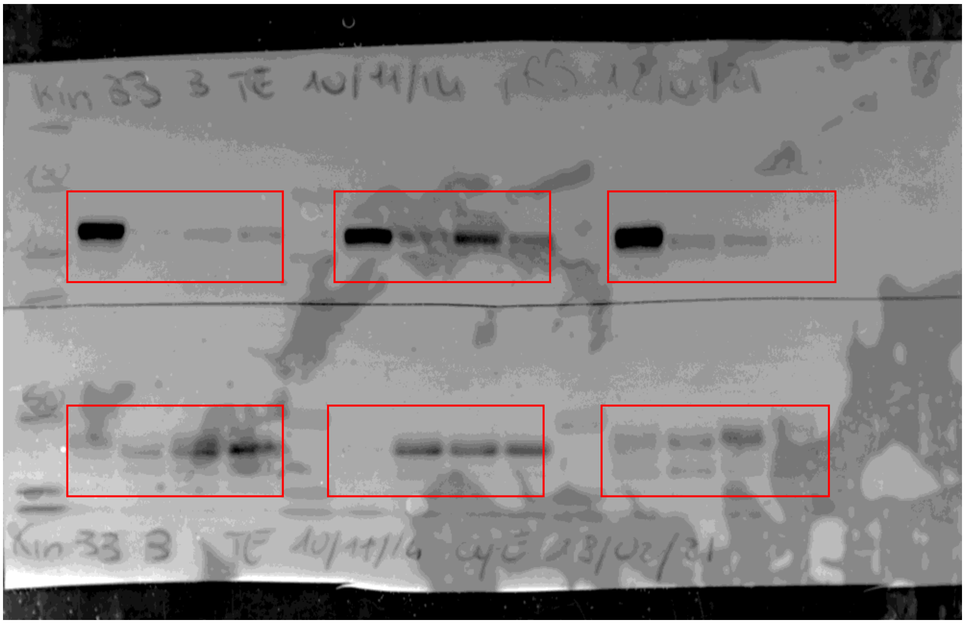

Rb pT807/T811

Cyclin E

TE-10 TE-11 TE-14

**Exposure 7 + marker**

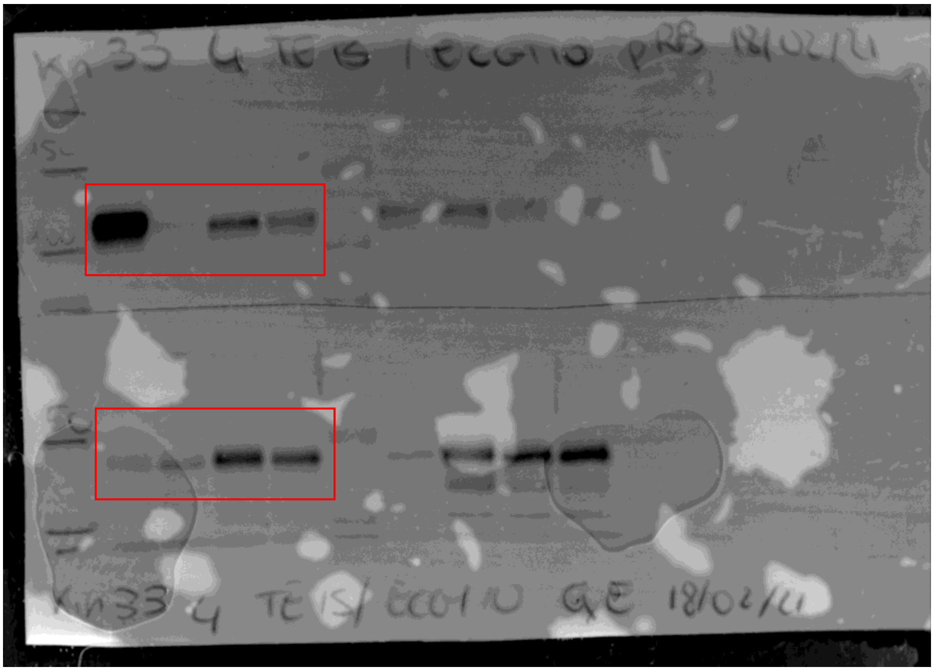

Rb pT807/T811

Cyclin E

**Exposure 6sum**

ECGI-10

100 KDa

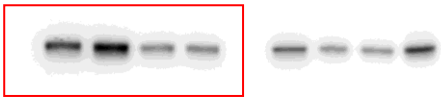

Rb pT807/T811

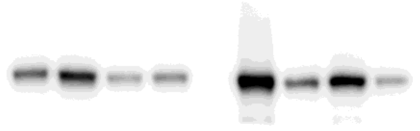

**Exposure 8**

ECGI-10

50 KDa

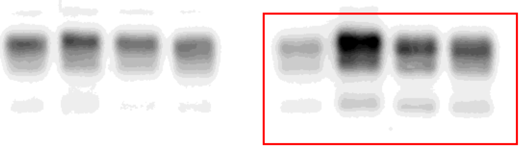

Cyclin E

Exposure 6 + marker

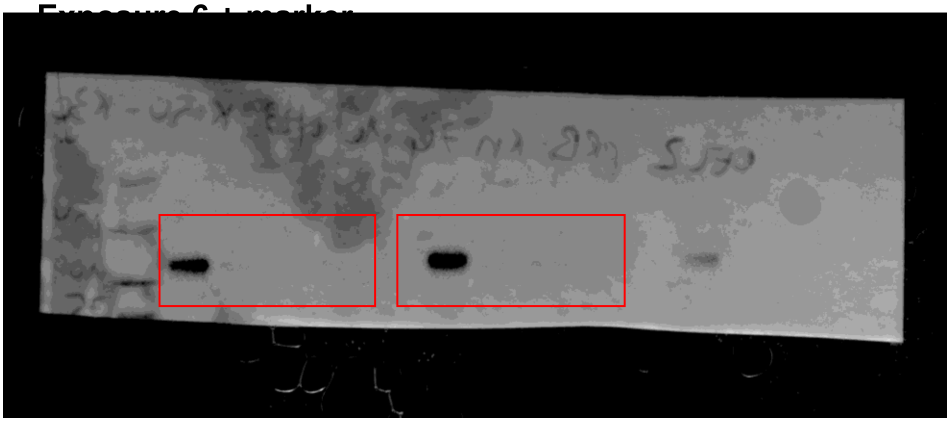

Rb pT807/T811

KYSE-30 KYSE-150

Exposure 6 + marker

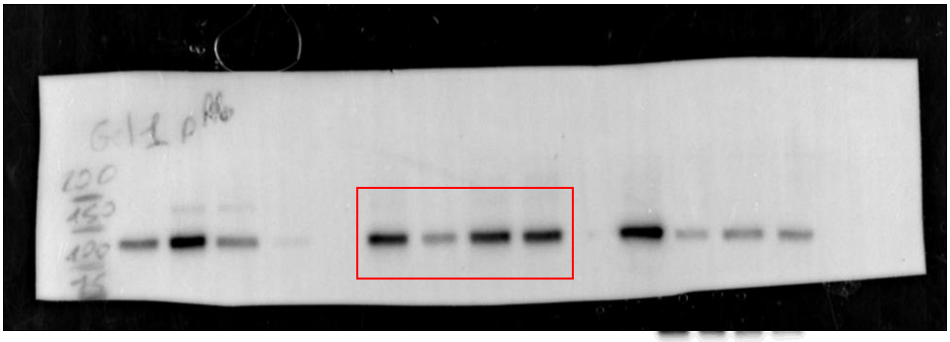

Rb pT807/T811

KYSE-70

Exposure 6 + marker

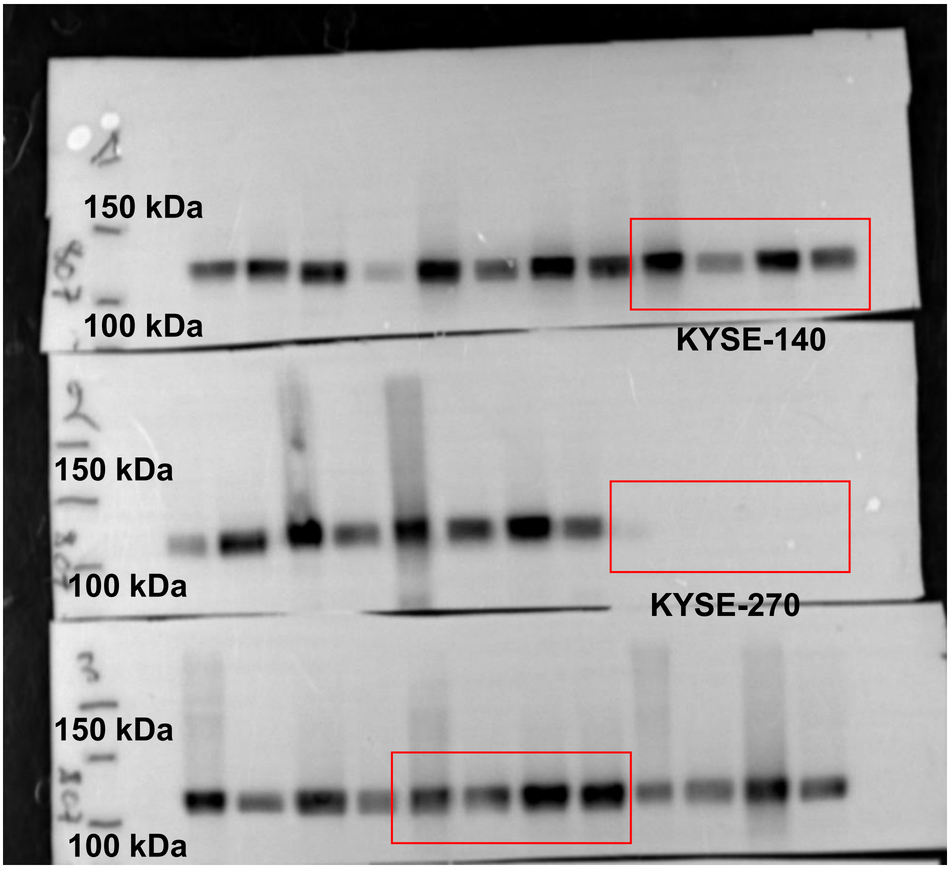


Rb pT807/T811

Rb pT807/T811

Rb pT807/T811

KYSE-450

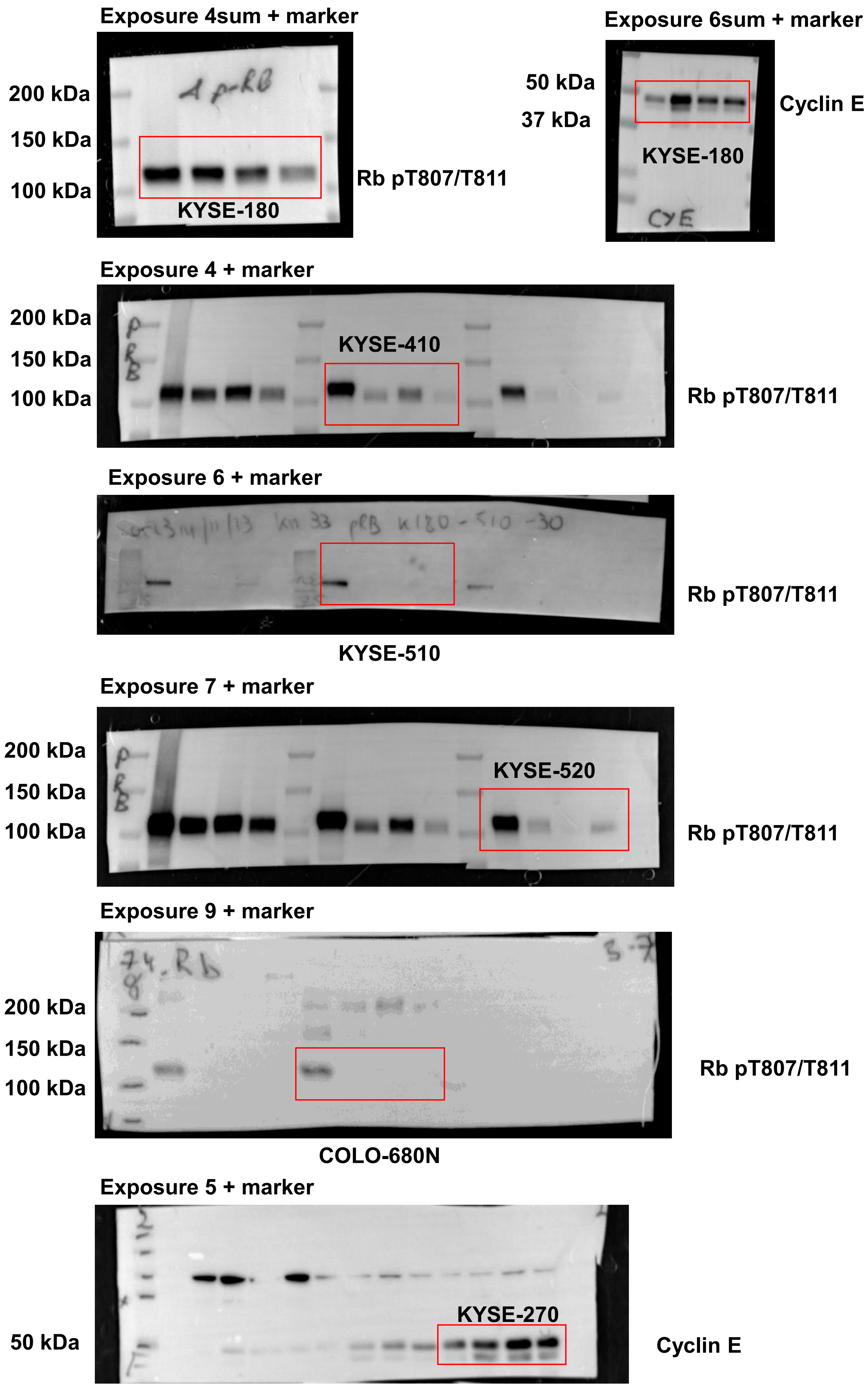

Exposure 4sum + marker
200 kDa
150 kDa
100 kDa
KYSE-180
Rb pT807/T811
Exposure 6sum + marker
50 kDa
37 kDa
Cyclin E
KYSE-180
Exposure 4 + marker
200 kDa
150 kDa
100 kDa
KYSE-410
Rb pT807/T811
Exposure 6 + marker
Rb pT807/T811
KYSE-510
Exposure 7 + marker
200 kDa
150 kDa
100 kDa
KYSE-520
Rb pT807/T811
Exposure 9 + marker
200 kDa
150 kDa
100 kDa
Rb pT807/T811
COLO-680N
Exposure 5 + marker
KYSE-270
50 kDa
Cyclin E

Exposure 7sum + marker

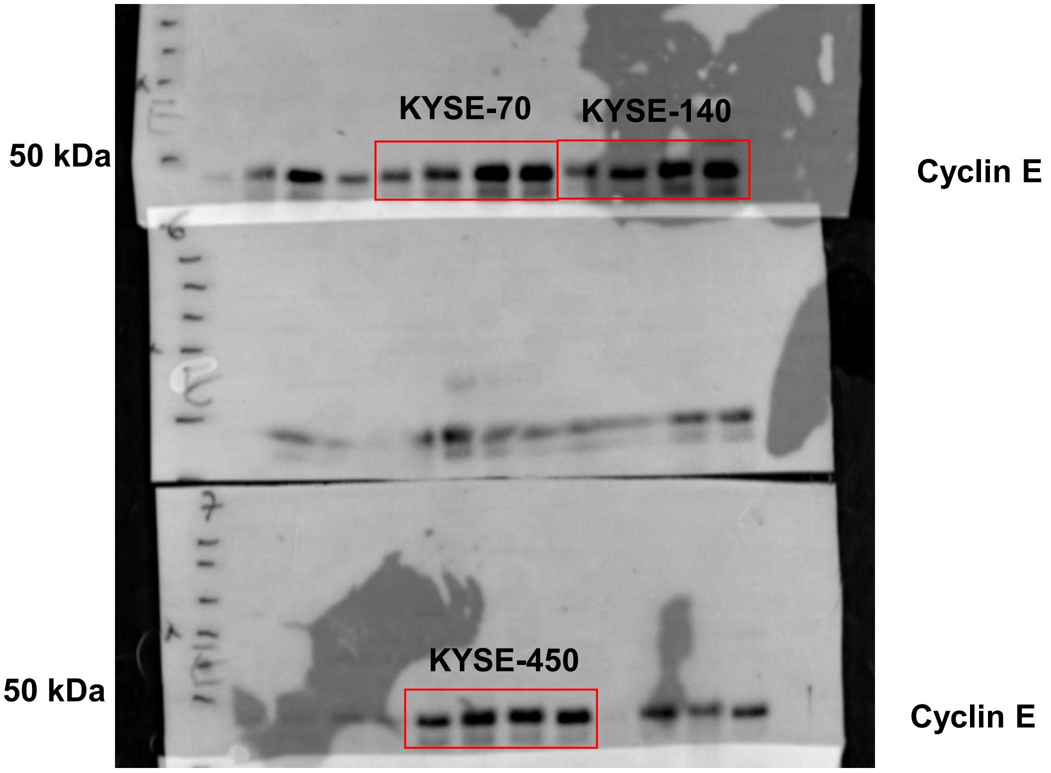


Exposure 3sum + marker

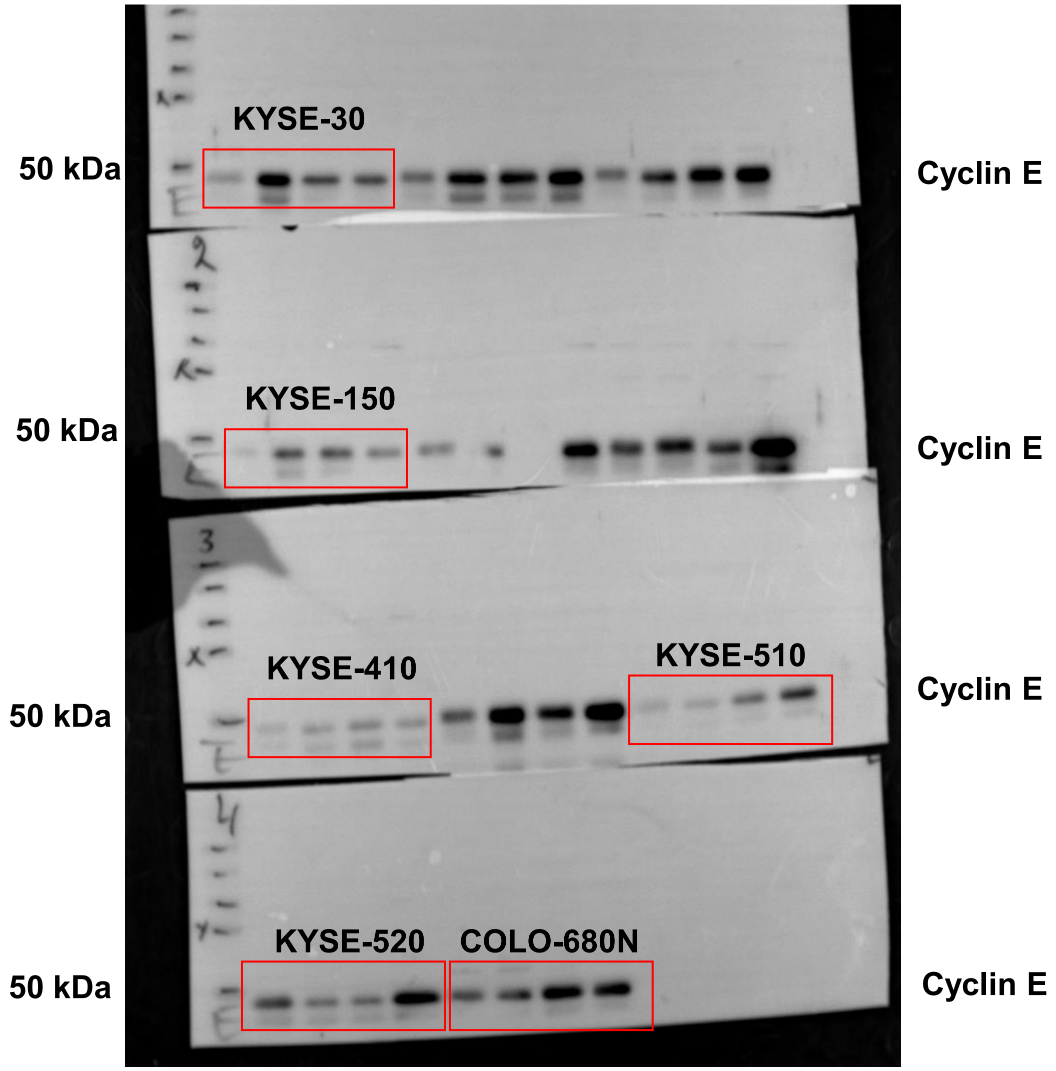

Exposure 7 + marker

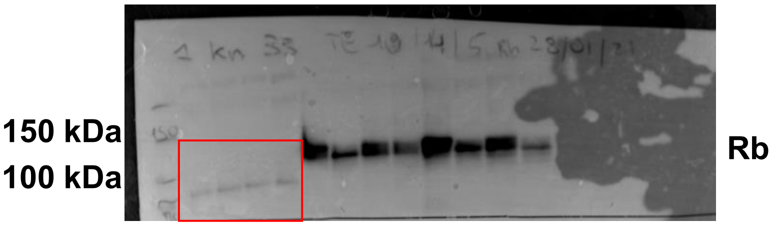


Exposure 7 + marker

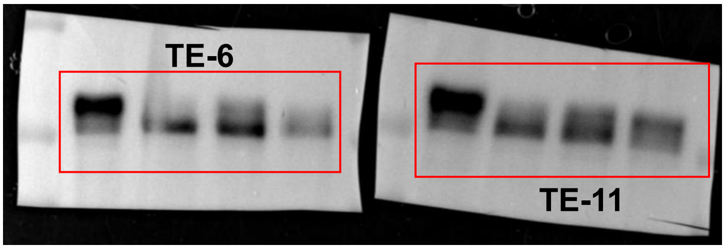


Exposure 7sum + marker

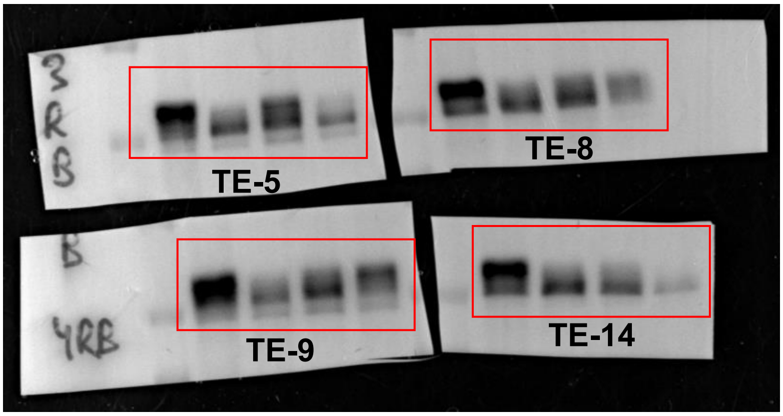


Exposure 5sum + marker

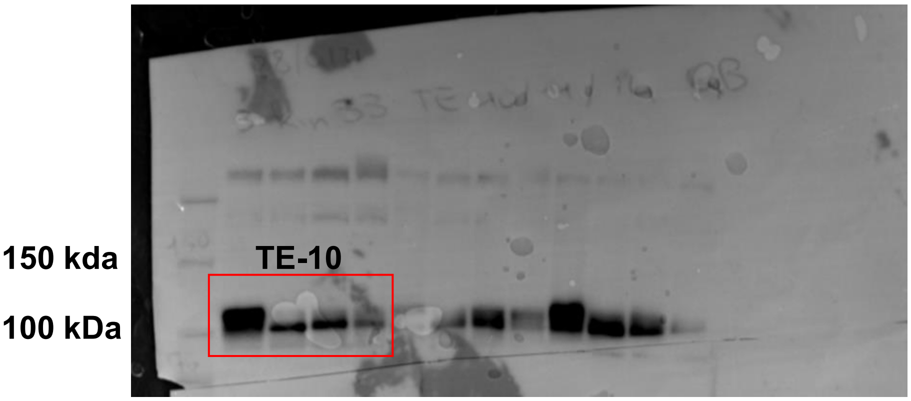


Exposure 6sum + marker

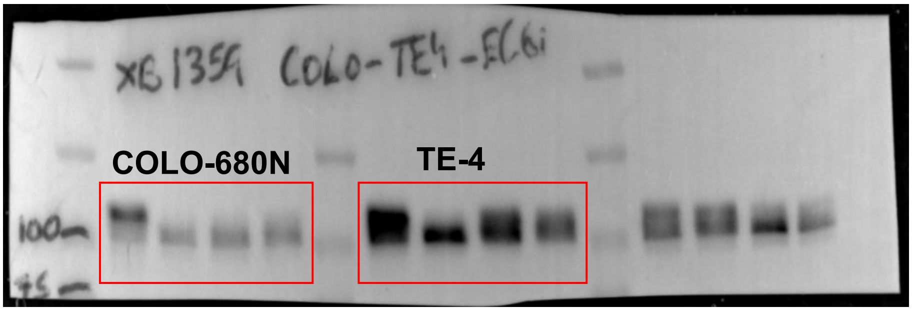

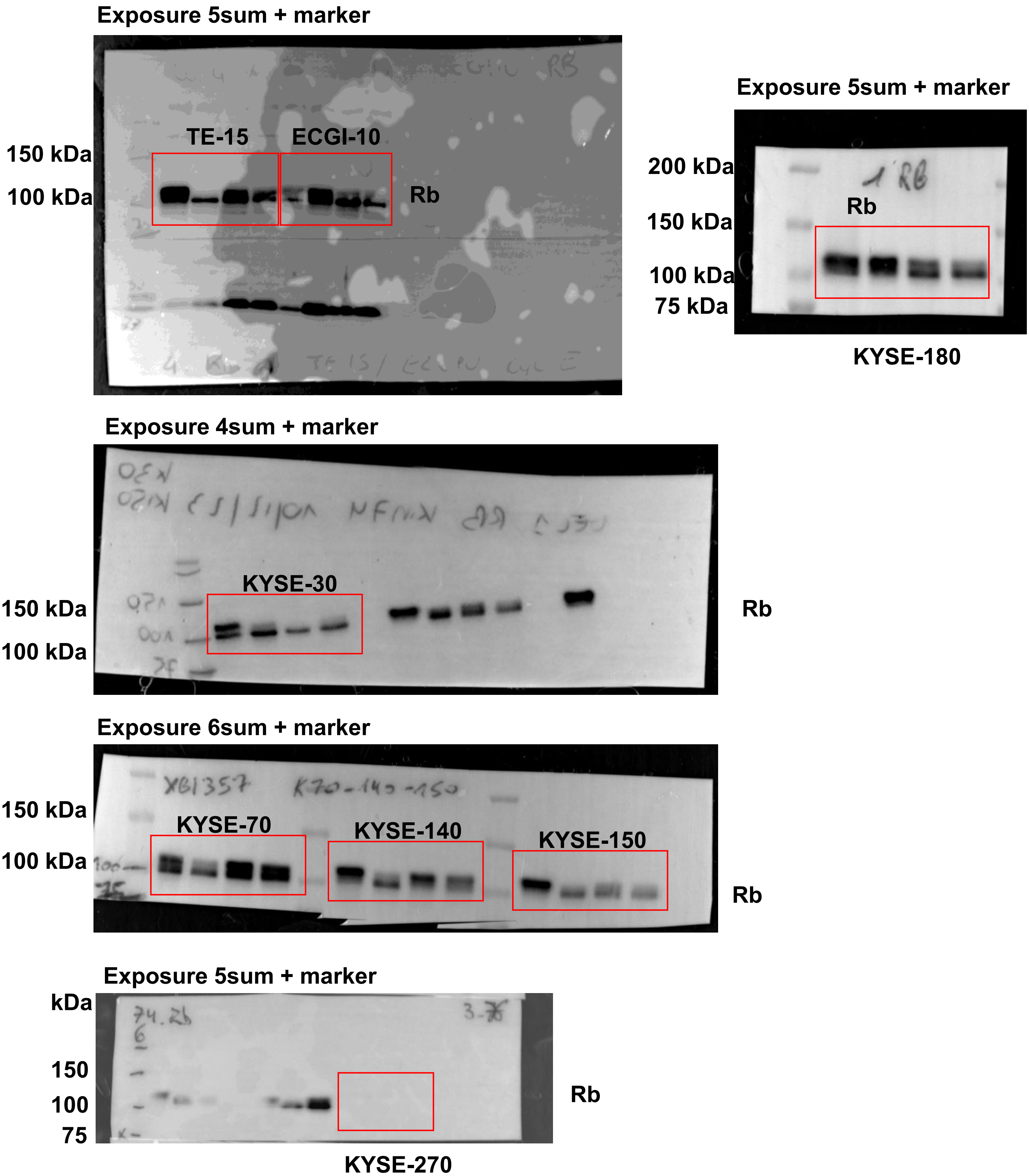

Exposure 5sum + marker
150 kDa
100 kDa
TE-15
ECGI-10
Rb
Exposure 5sum + marker
200 kDa
150 kDa
100 kDa
75 kDa
Rb
KYSE-180
Exposure 4sum + marker
150 kDa
100 kDa
KYSE-30
Rb
Exposure 6sum + marker
150 kDa
100 kDa
KYSE-70
KYSE-140
KYSE-150
Rb
Exposure 5sum + marker
kDa
150
100
75
Rb
KYSE-270

Exposure 8 + marker

150 kDa
100 kDa

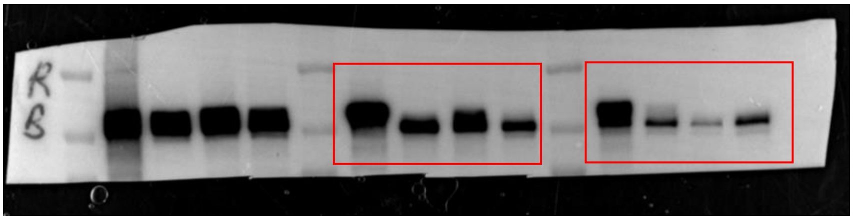

KYSE-410 KYSE-520

Exposure 6sum + marker

150 kDa
100 kDa

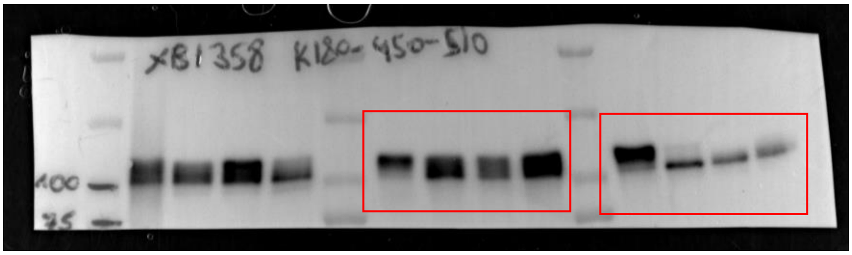

Rb

KYSE-450 KYSE-510

Exposure 5 + marker

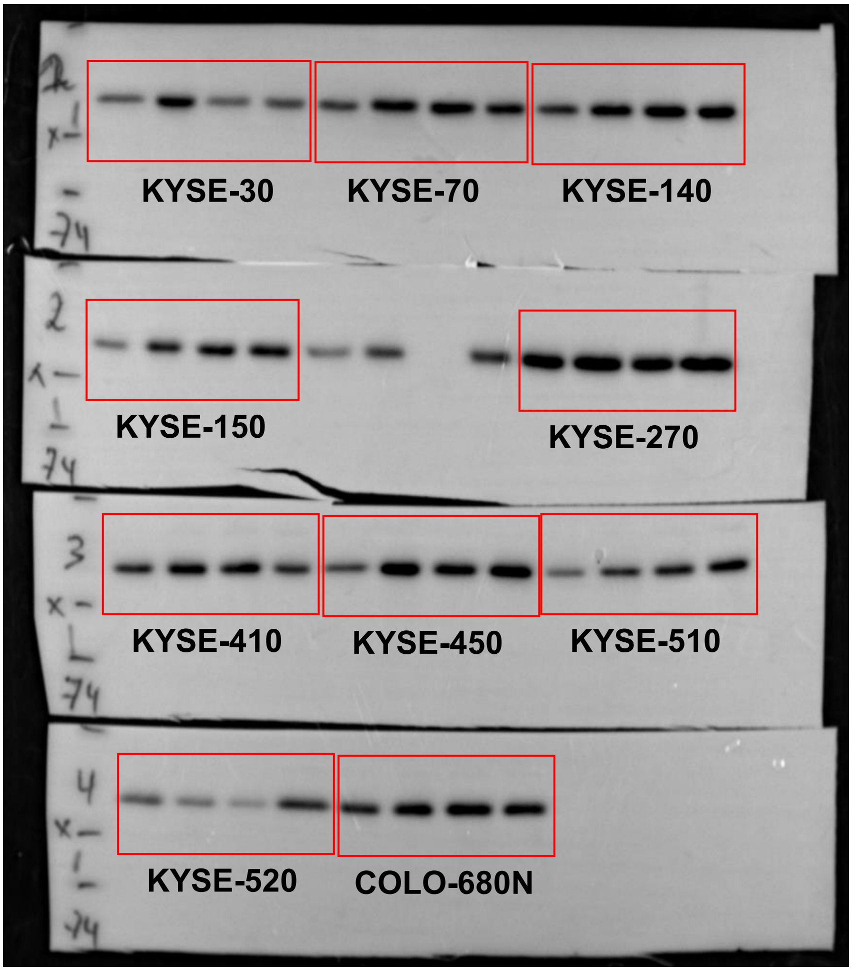


25 kDa
20 kDa
CDK4

25 kDa
20 kDa
CDK4

25 kDa
20 kDa
CDK4

25 kDa
20 kDa
CDK4

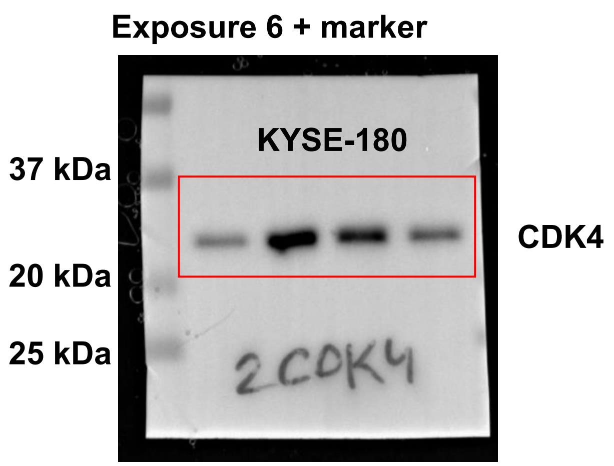
Exposure 6 + marker
KYSE-180
37 kDa
20 kDa
25 kDa
CDK4
2 CDK4

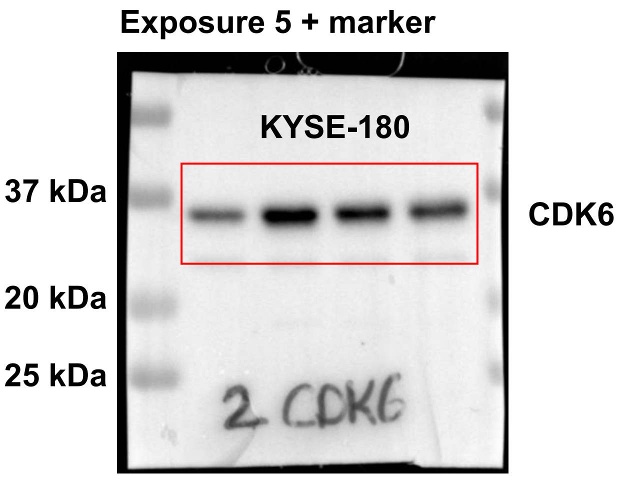
Exposure 5 + marker
KYSE-180
37 kDa
20 kDa
25 kDa
CDK6
2 CDK6

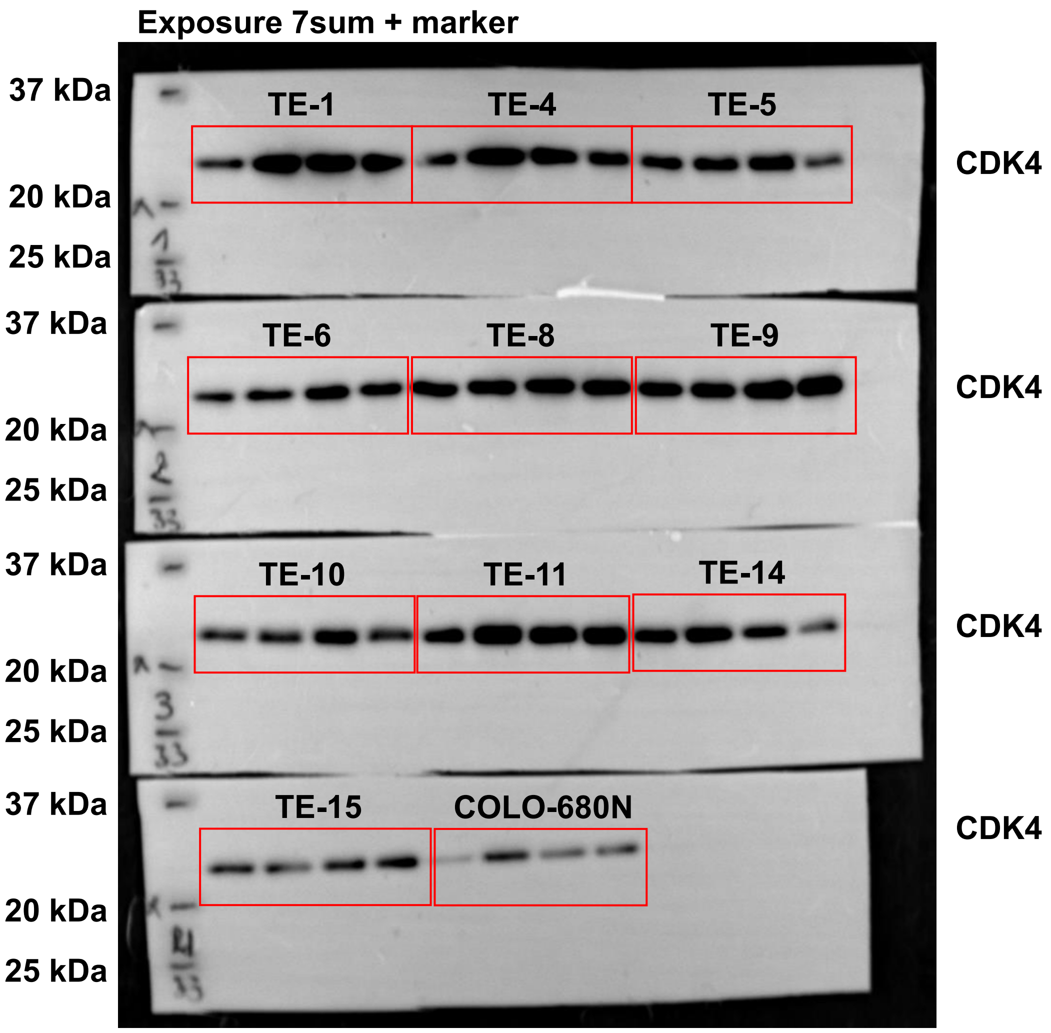
Exposure 7sum + marker
37 kDa
20 kDa
25 kDa
TE-1
TE-4
TE-5
CDK4
TE-6
TE-8
TE-9
CDK4
TE-10
TE-11
TE-14
CDK4
TE-15
COLO-680N
CDK4

Exposure 3sum + marker

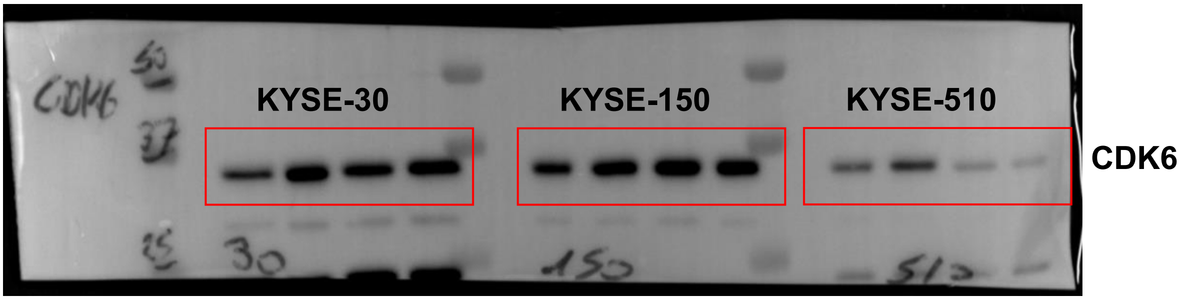


Exposure 3sum + marker

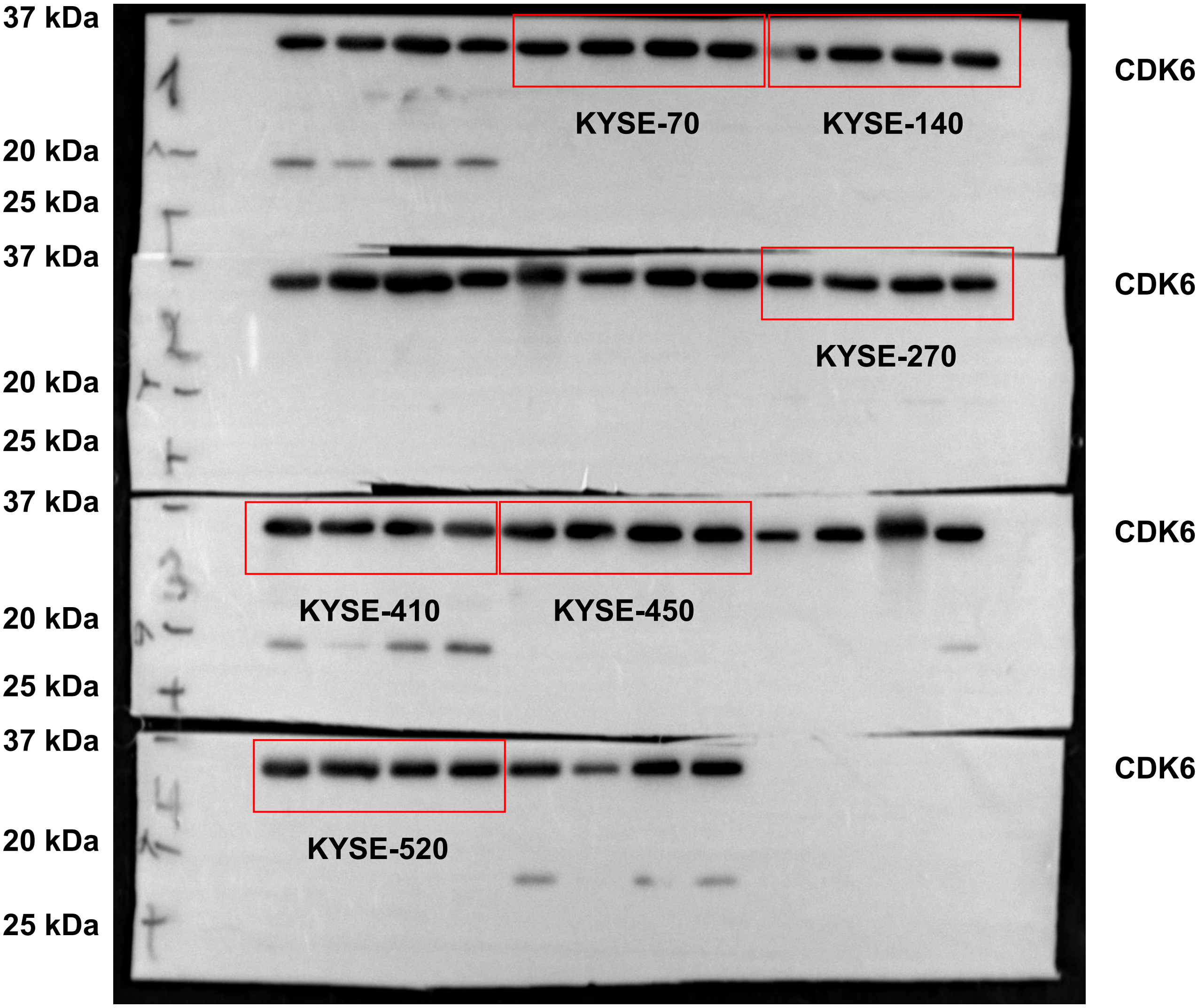

Exposure 3 + marker

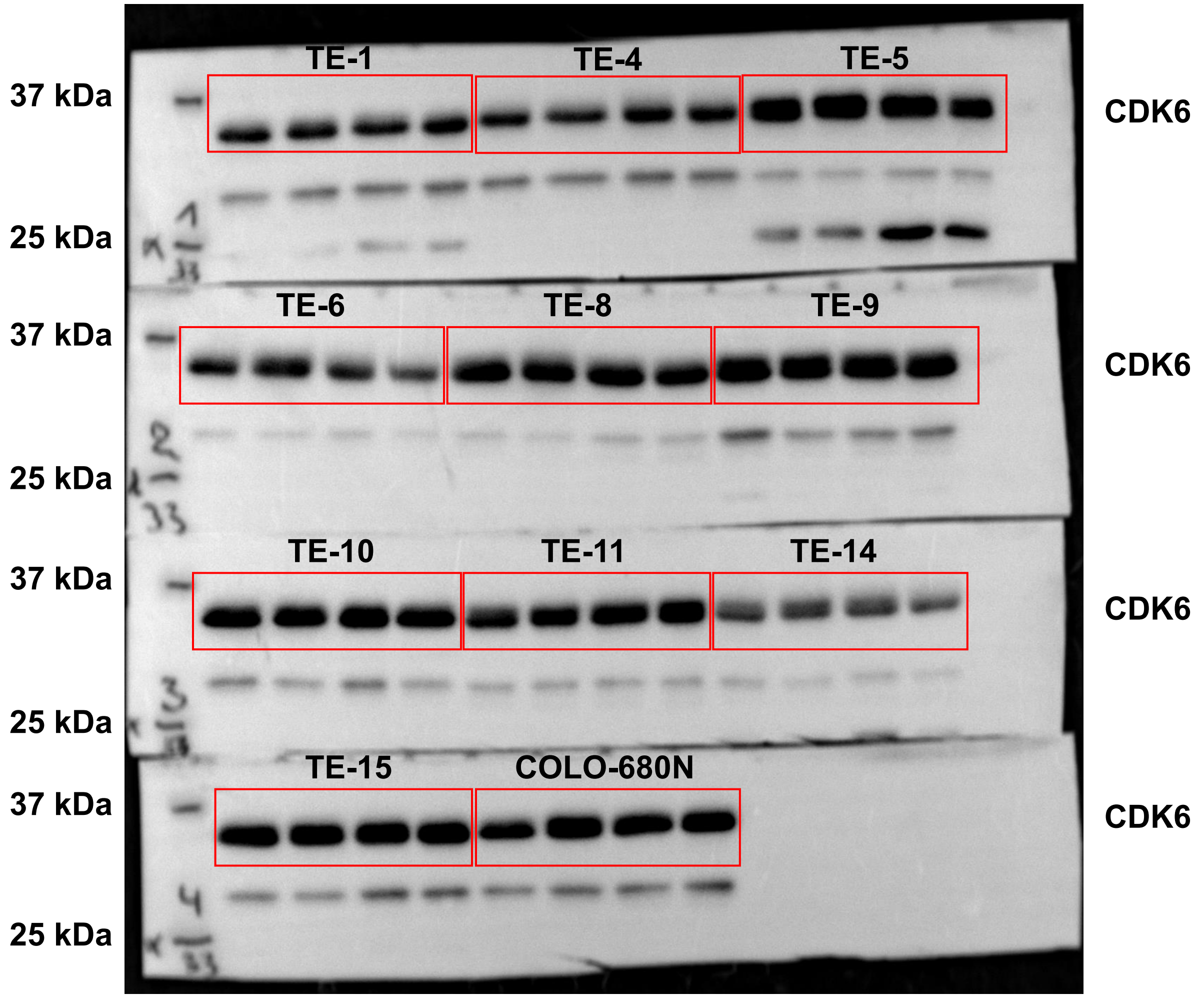


Exposure 6 + marker

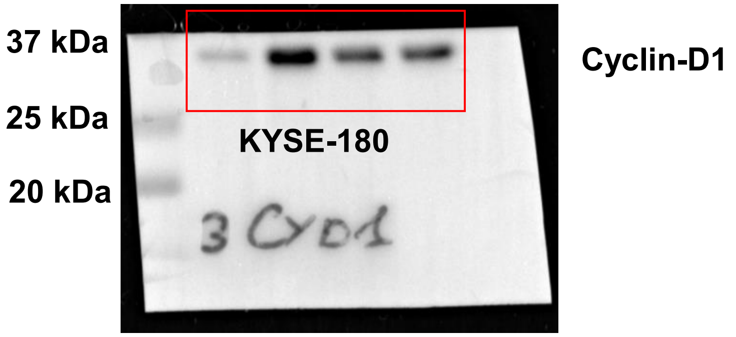


Exposure 6 + marker

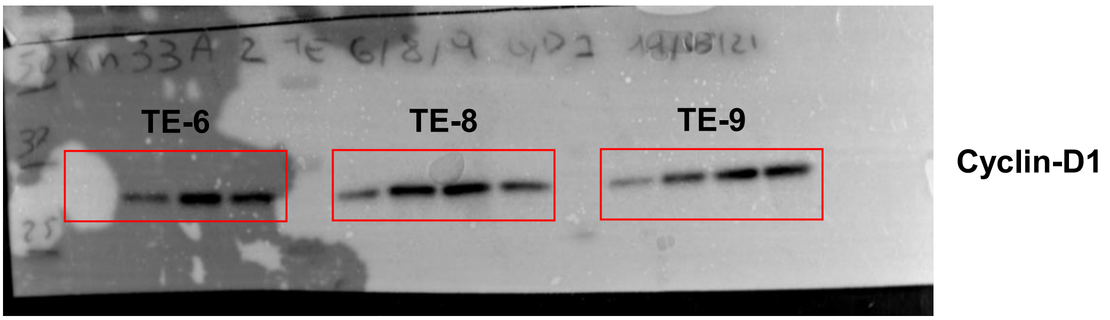

Exposure 2sum + marker

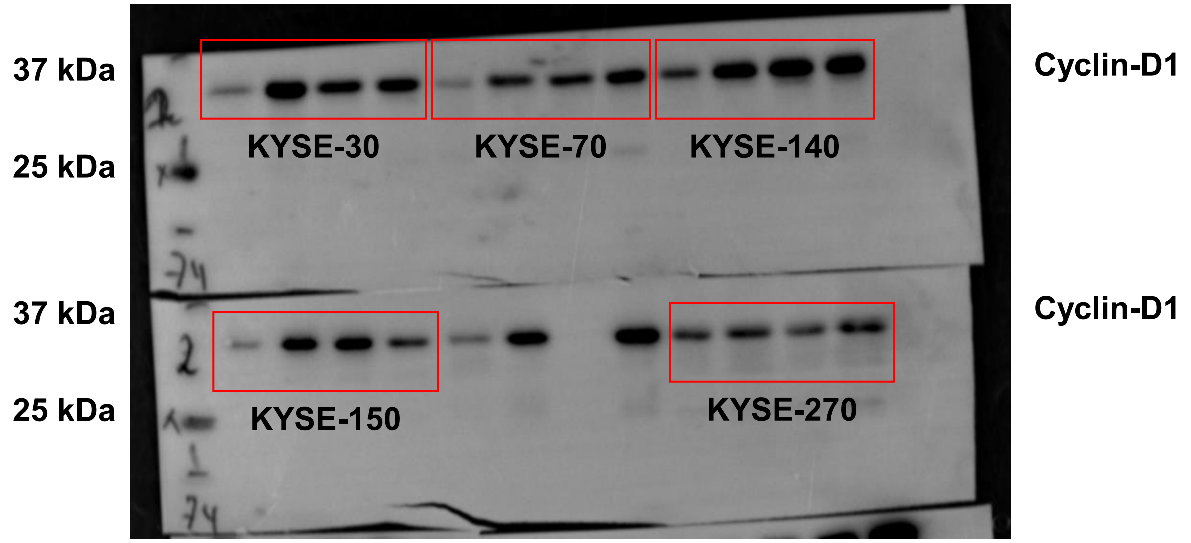


Exposure 2sum + marker

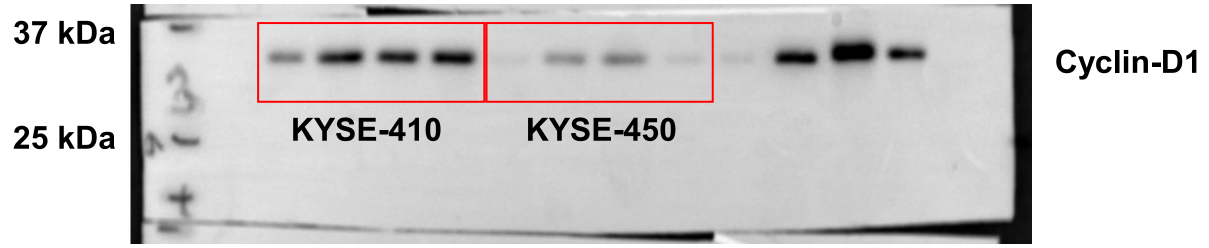


Exposure 2sum + marker

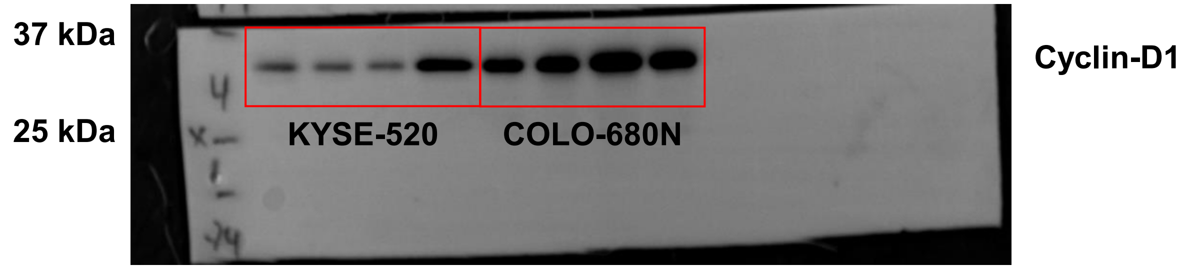


Exposure 6sum + marker

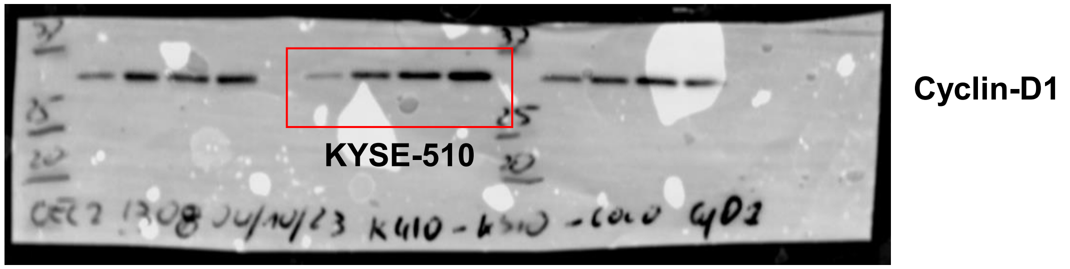

Exposure 8 + marker

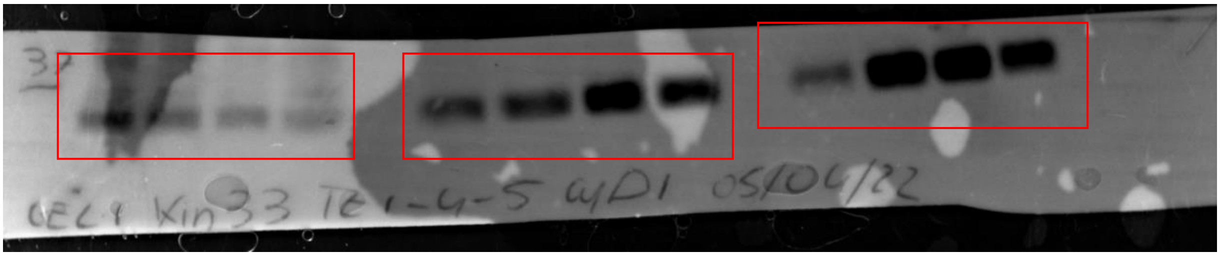

Cyclin-D1

TE-1 TE-4 TE-5

Exposure 8sum + marker

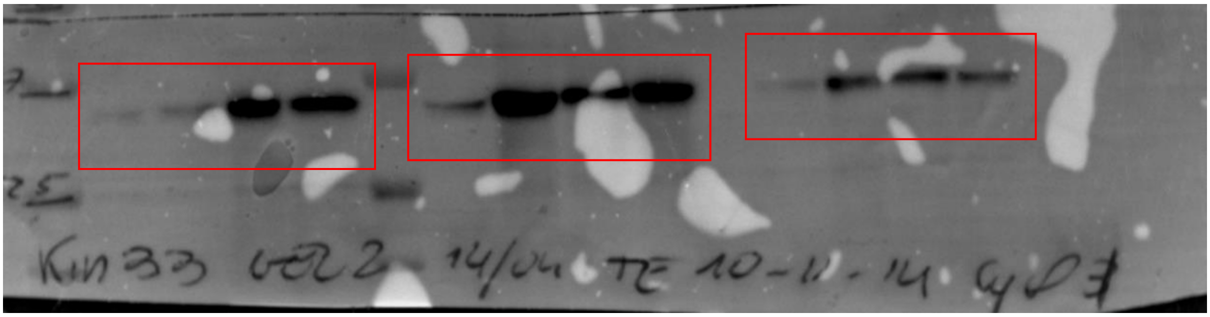

Cyclin-D1

TE-10 TE-11 TE-14

Exposure 5sum + marker

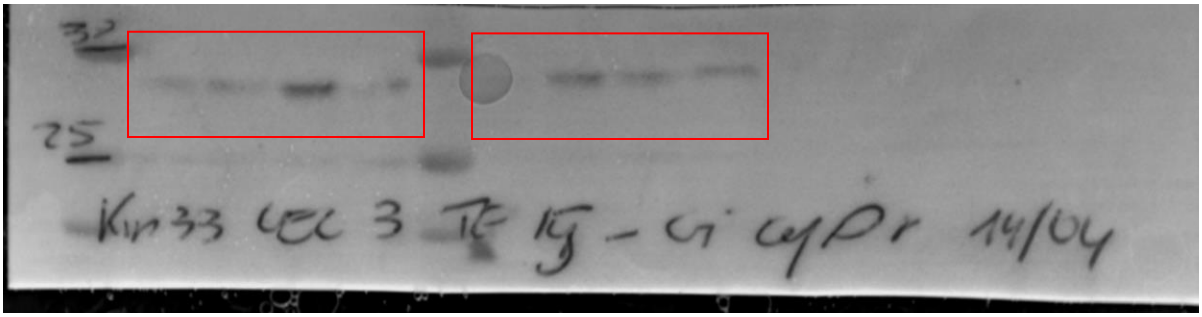

Cyclin-D1

TE-15 ECGI-10

Exposure 7 + marker

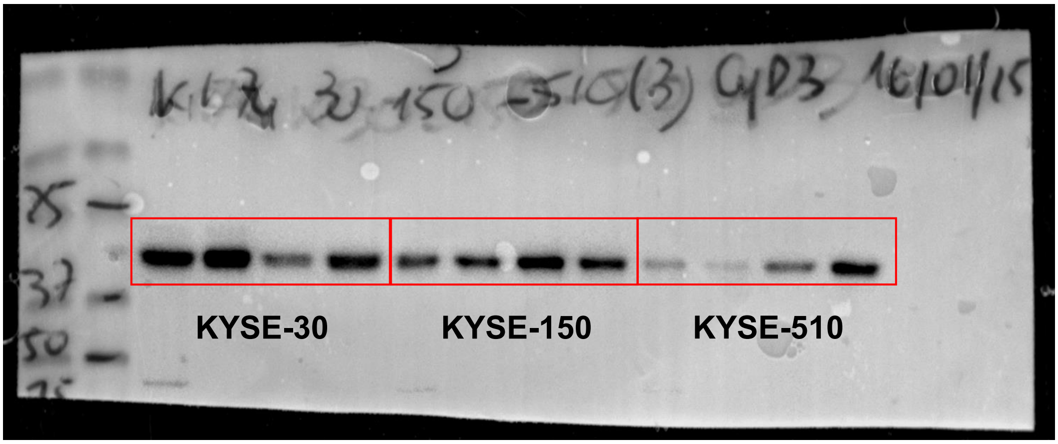


Cyclin-D3

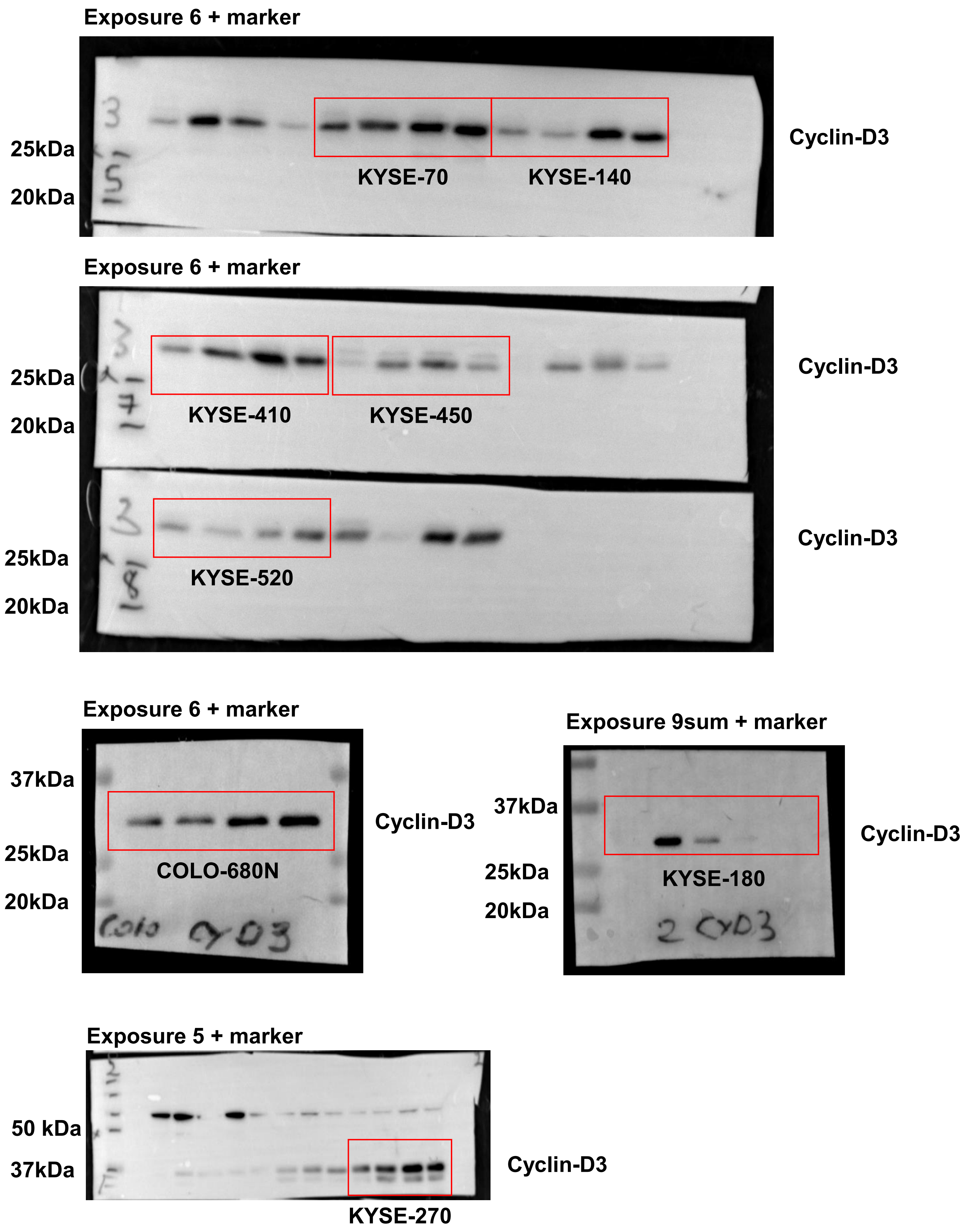
Exposure 6 + marker
25kDa
20kDa
KYSE-70
KYSE-140
Cyclin-D3
Exposure 6 + marker
25kDa
20kDa
KYSE-410
KYSE-450
Cyclin-D3
25kDa
20kDa
KYSE-520
Cyclin-D3
Exposure 6 + marker
37kDa
25kDa
20kDa
COLO-680N
Cyclin-D3
Exposure 9sum + marker
37kDa
25kDa
20kDa
KYSE-180
Cyclin-D3
Exposure 5 + marker
50 kDa
37kDa
KYSE-270
Cyclin-D3

Exposure 7sum + marker

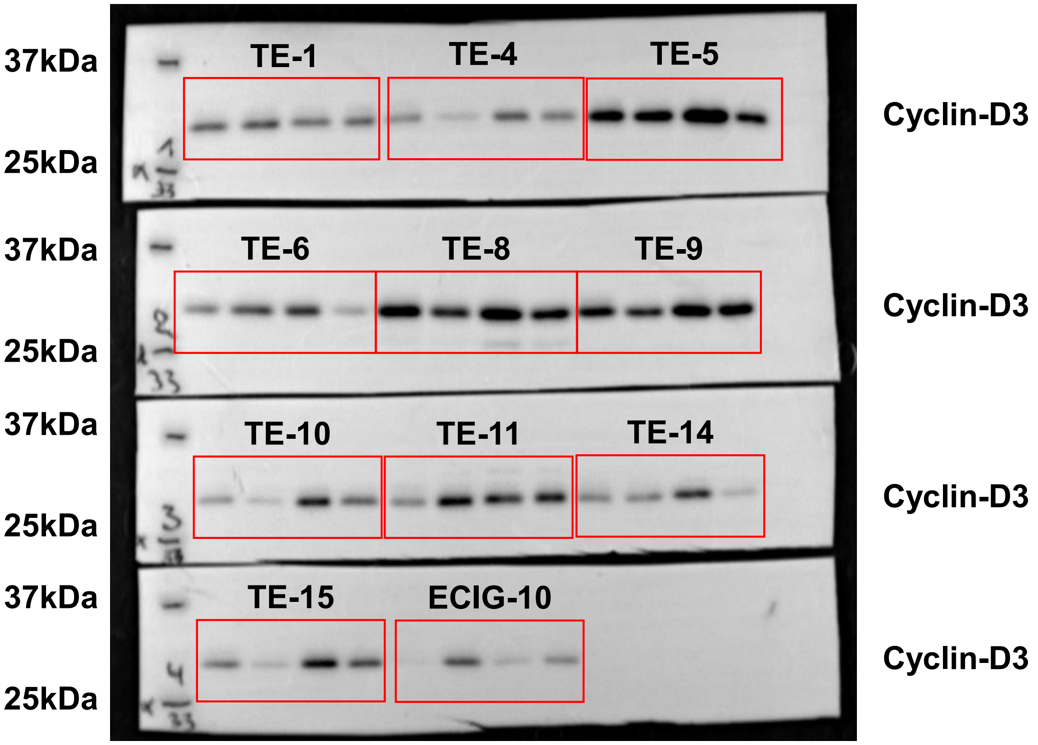


Exposure 10sum + marker

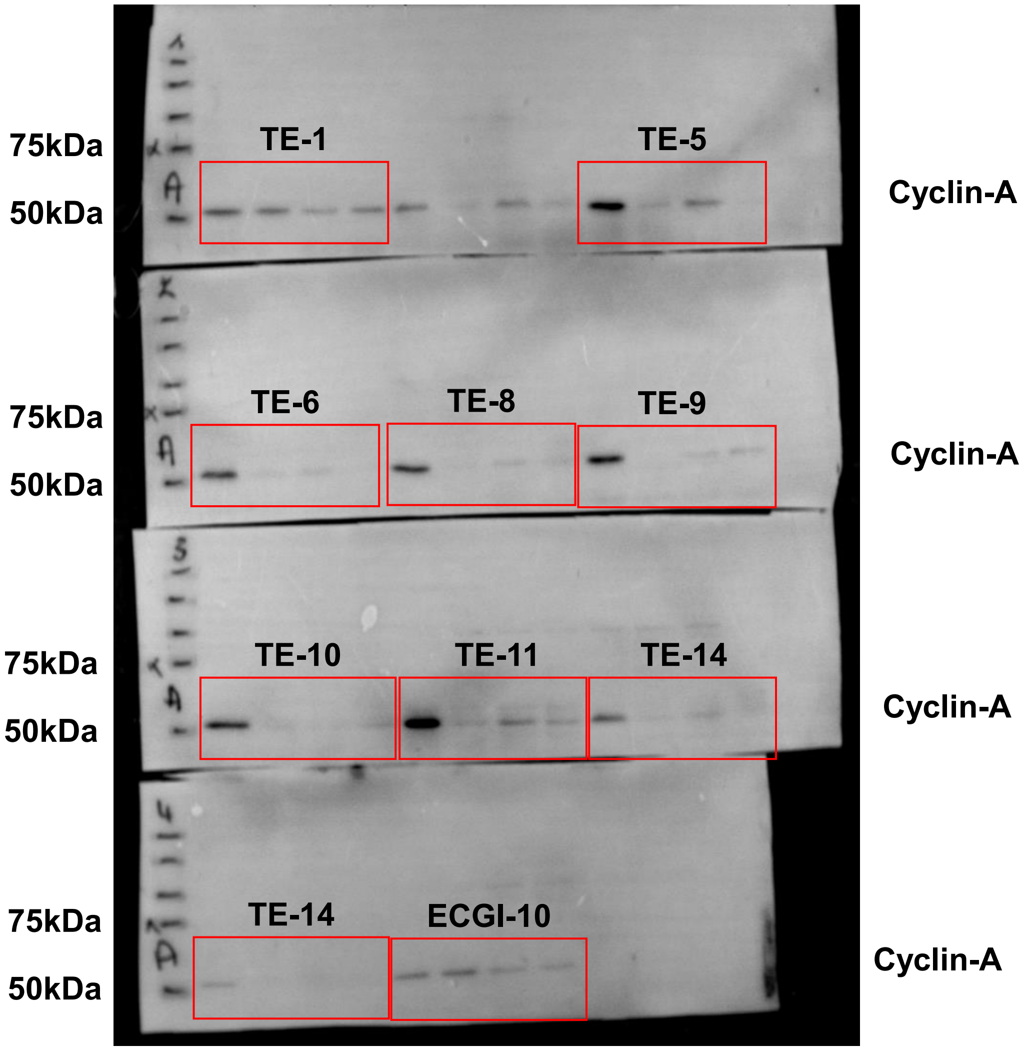


Exposure 7 + marker

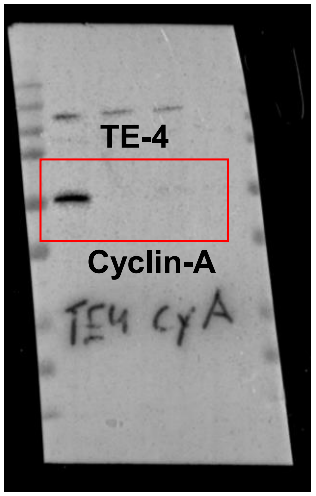

Exposure 10sum + marker

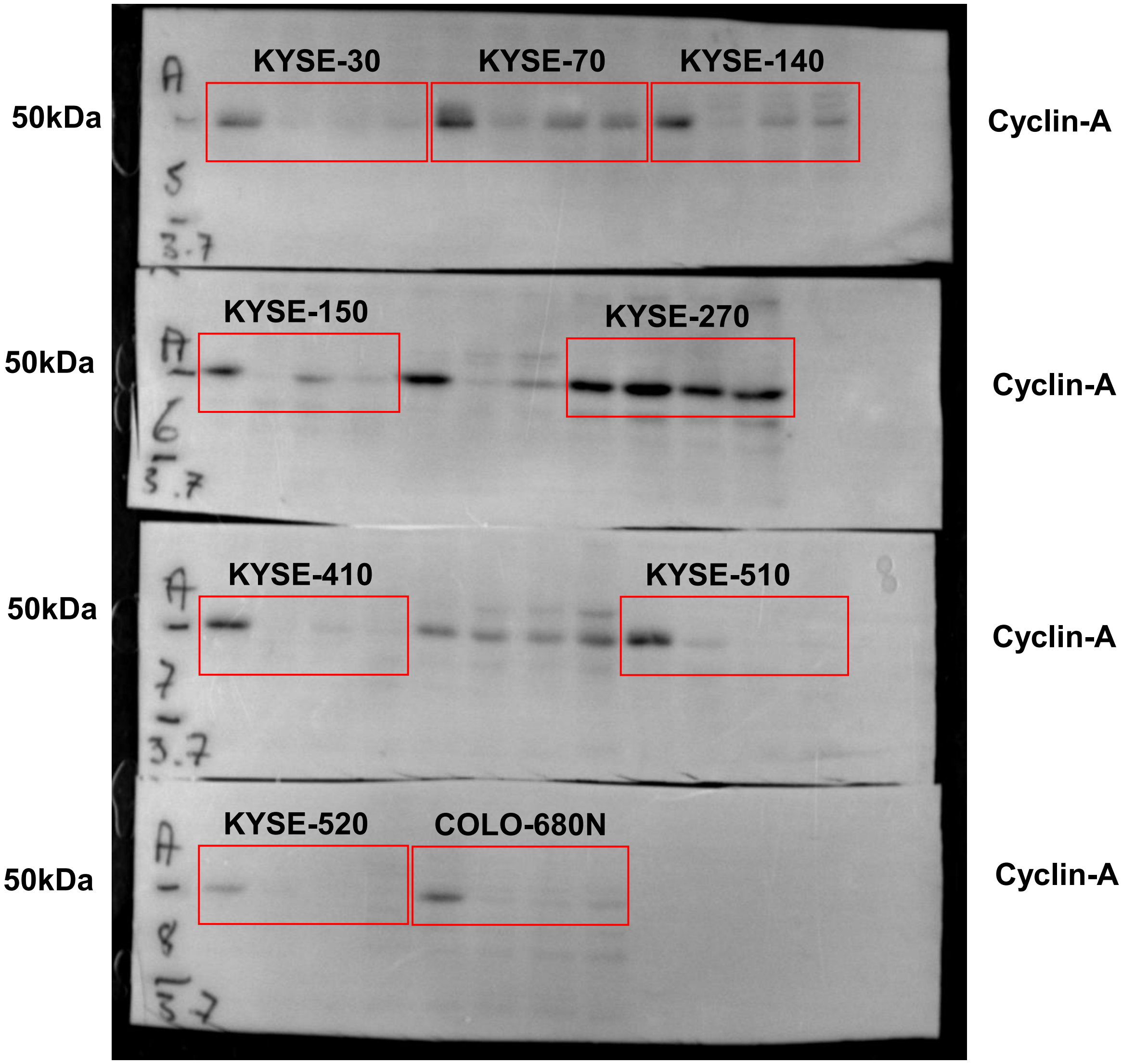


Exposure 8sum + marker

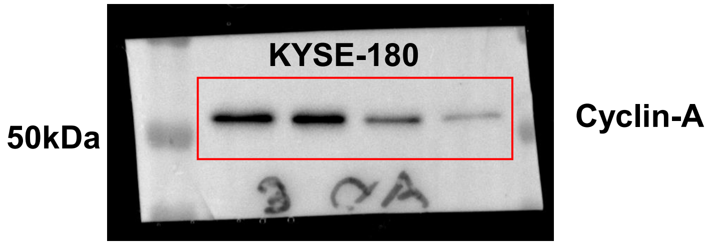


Exposure 9sum + marker

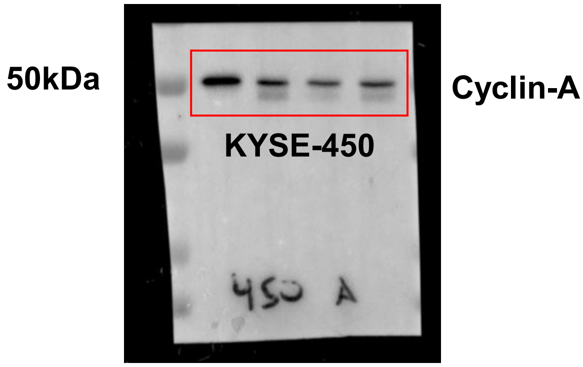

Exposure 9 + marker

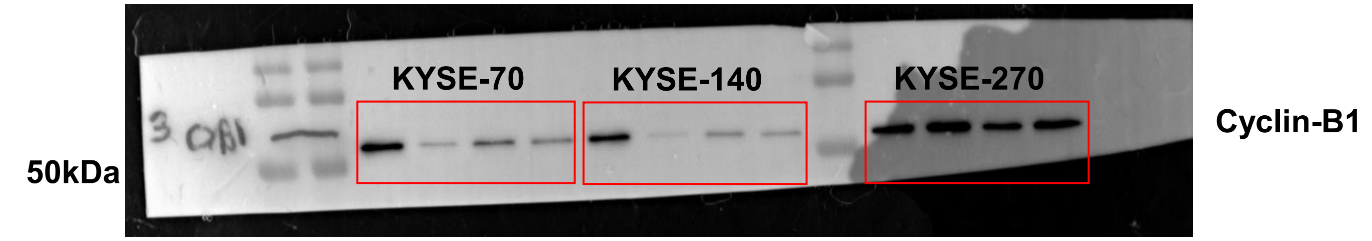


Exposure 9sum + marker

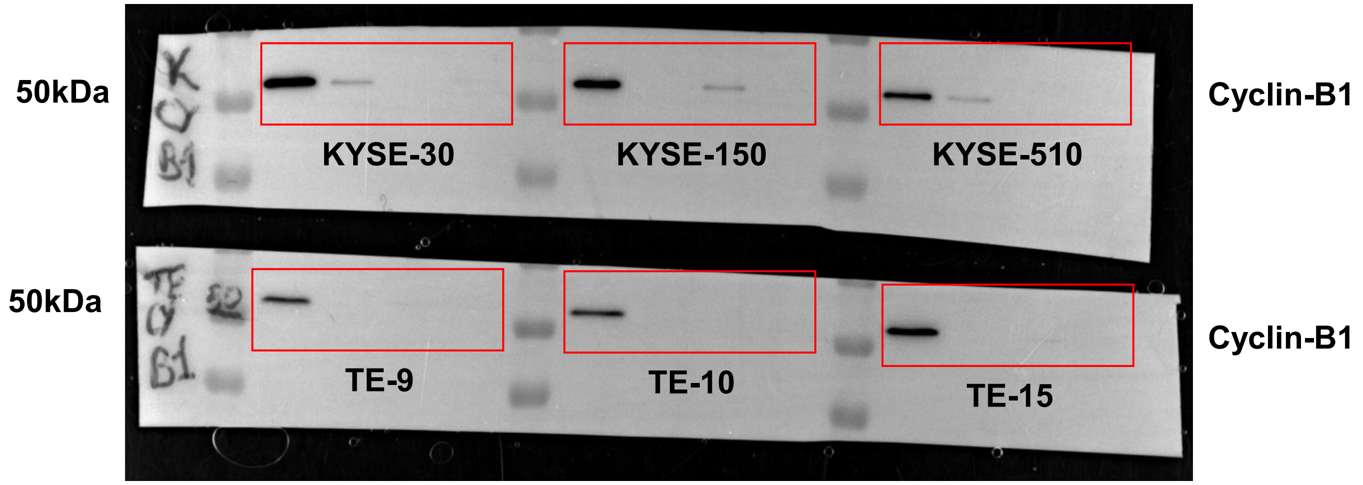


Exposure 9sum + marker

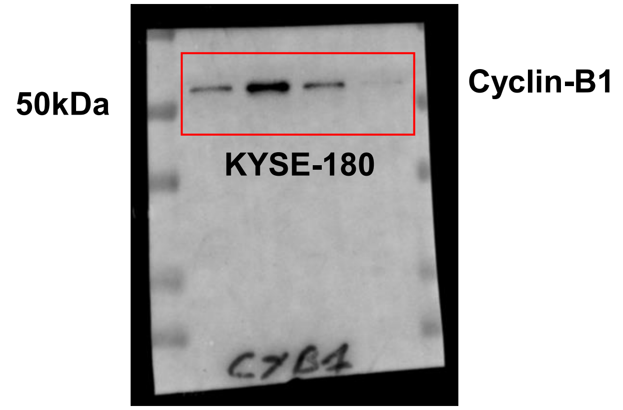


Exposure 6sum + marker

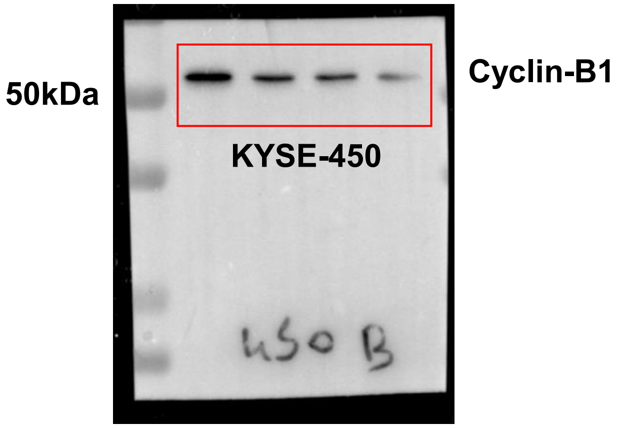


Exposure 5 + marker

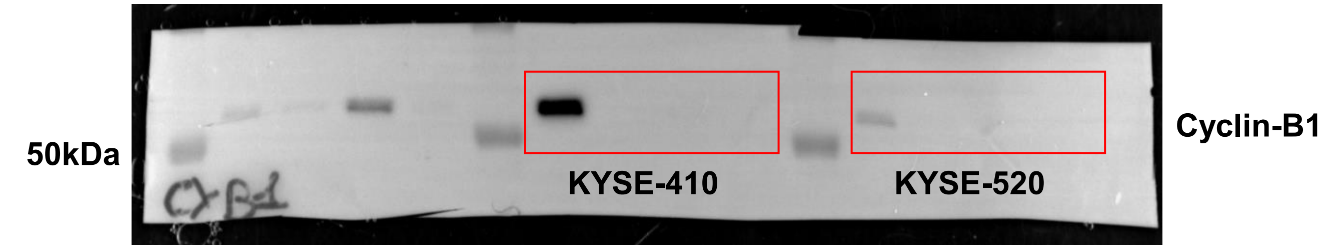

Exposure 9sum + marker

50kDa

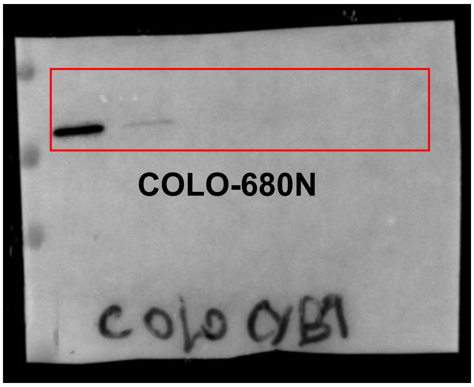


Cyclin-B1

Exposure 9sum + marker

50kDa

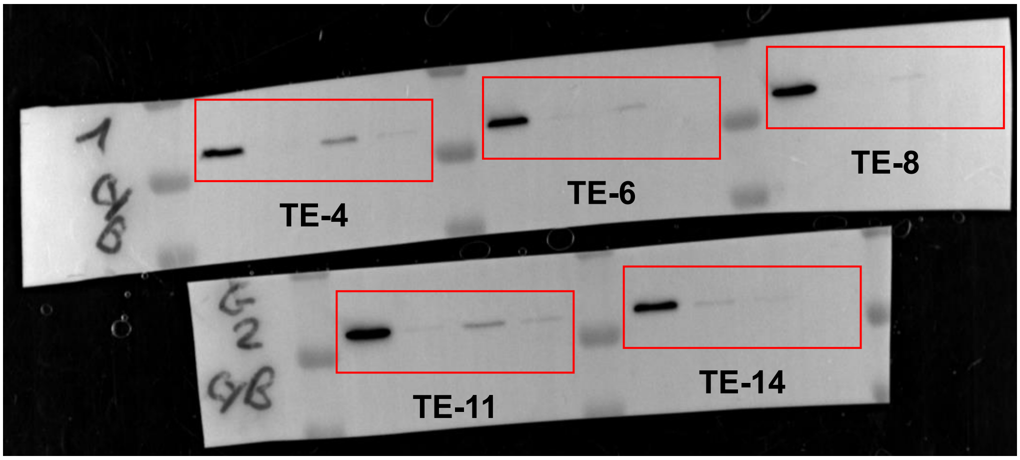


Cyclin-B1

Exposure 9

50kDa

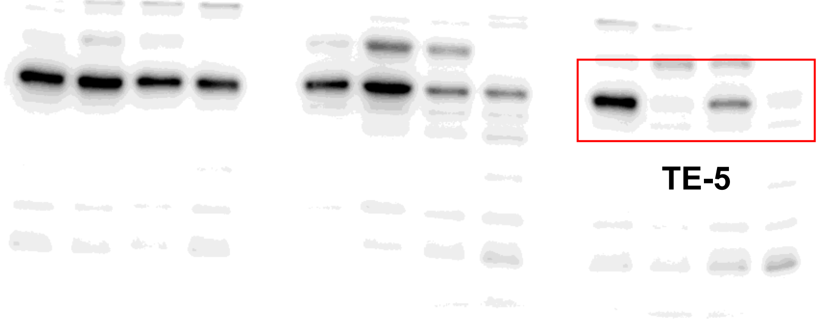


Cyclin-B1

Exposure 5sum

50kDa

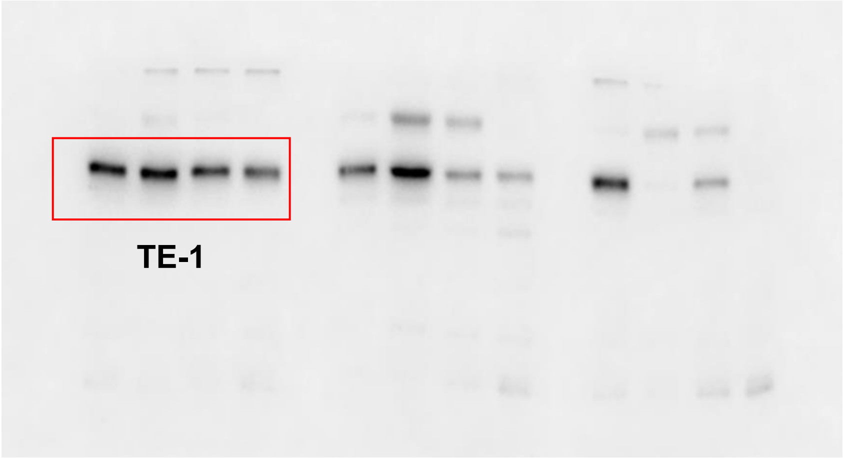


Cyclin-B1

Exposure 9sum + marker

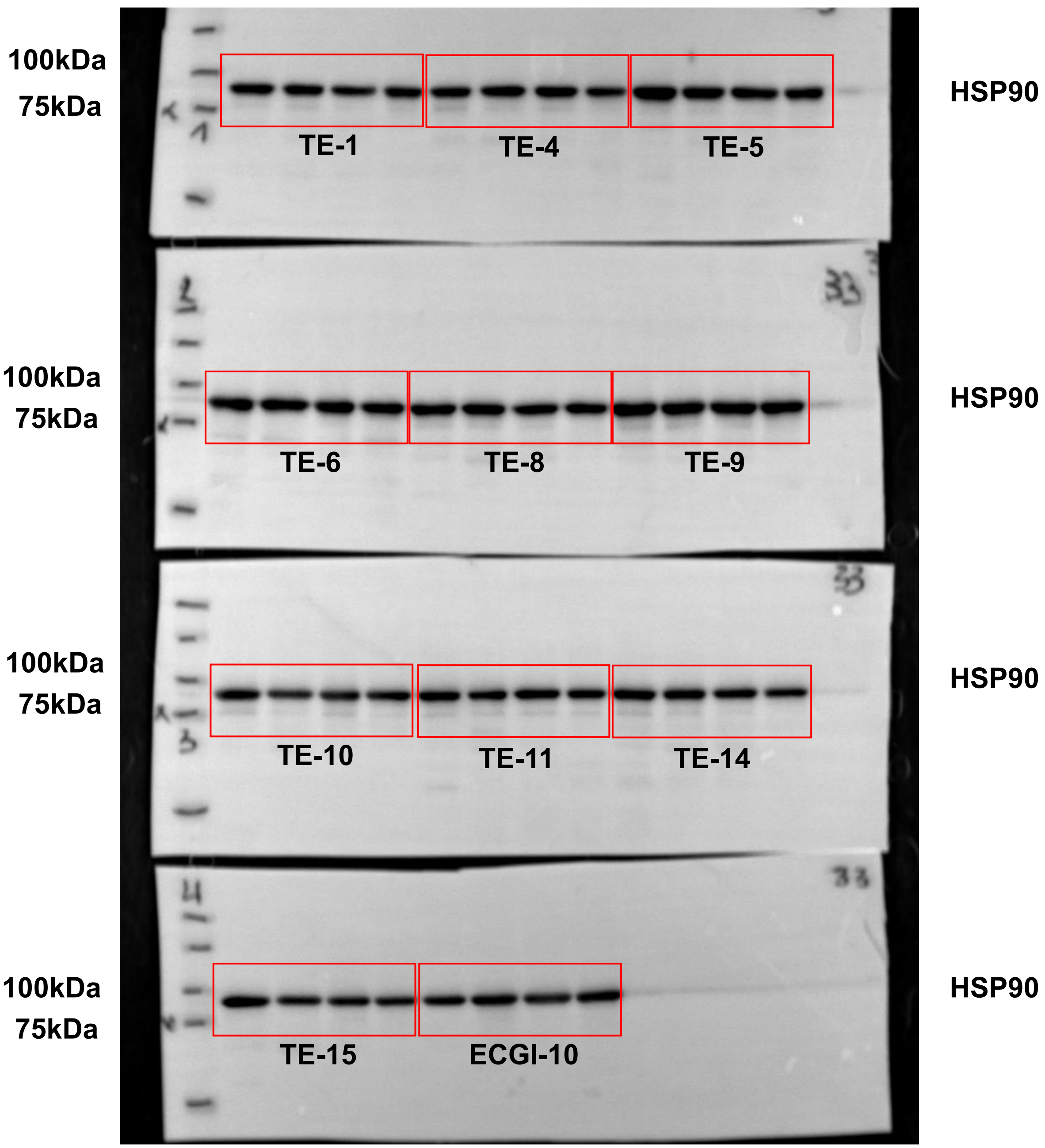


Exposure 2sum + marker

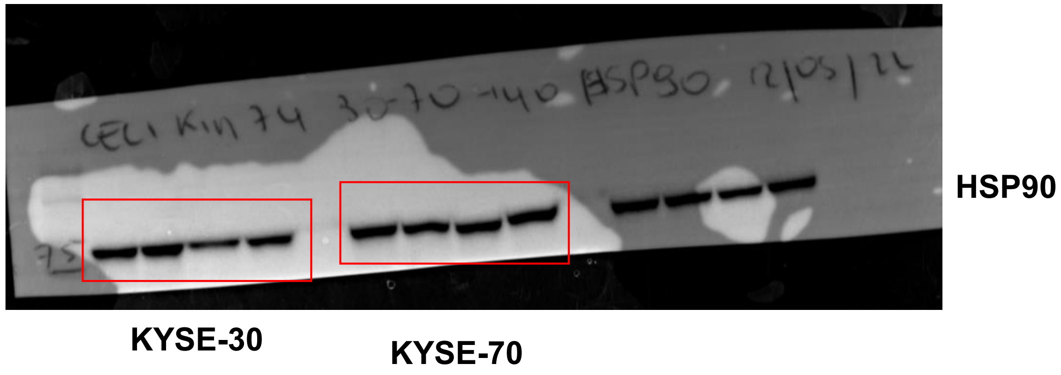

Exposure 3sum + marker

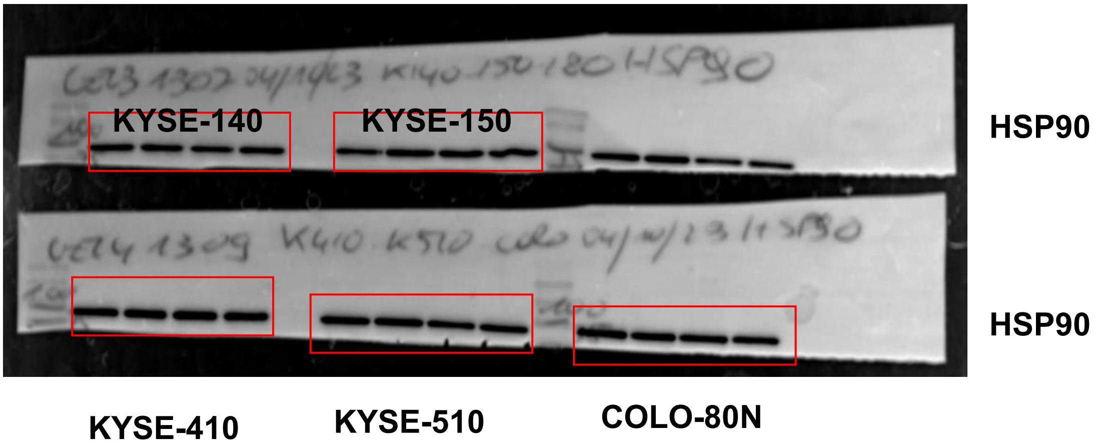


Exposure 2sum + marker

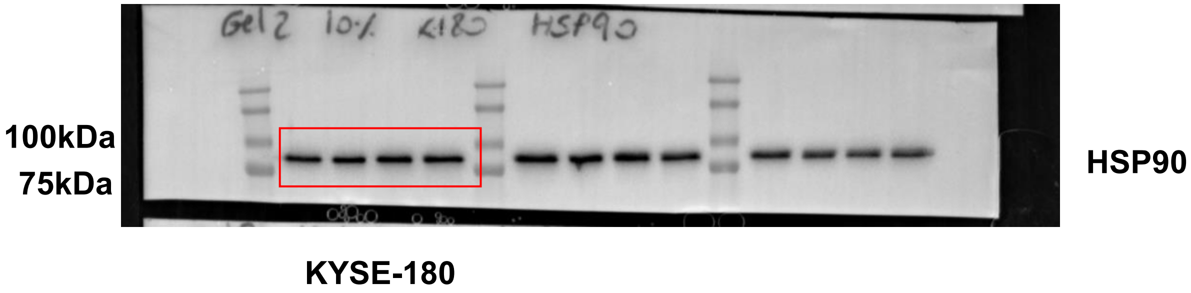


Exposure 2sum + marker

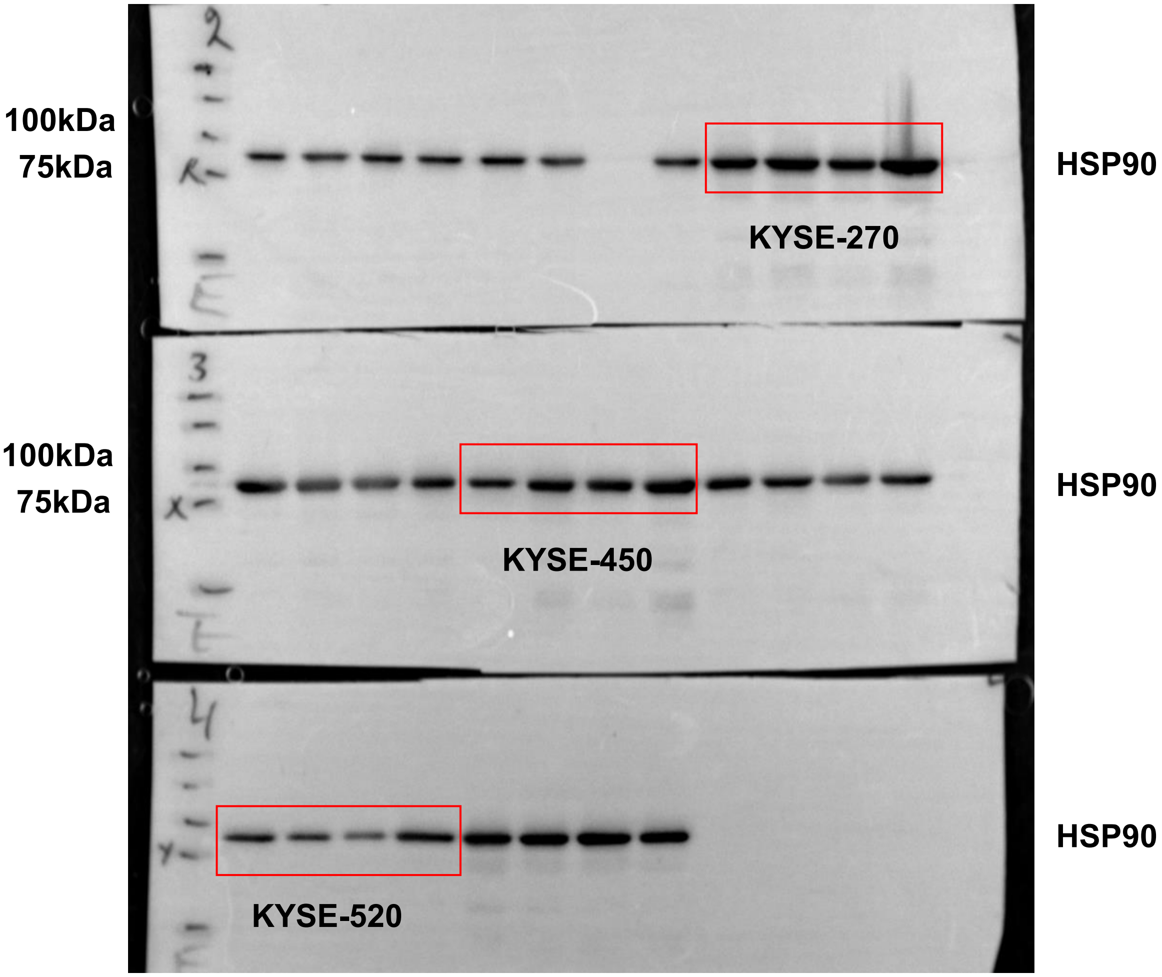

# Figure 4 & supplementary Figure 4

Exposure 5 + marker

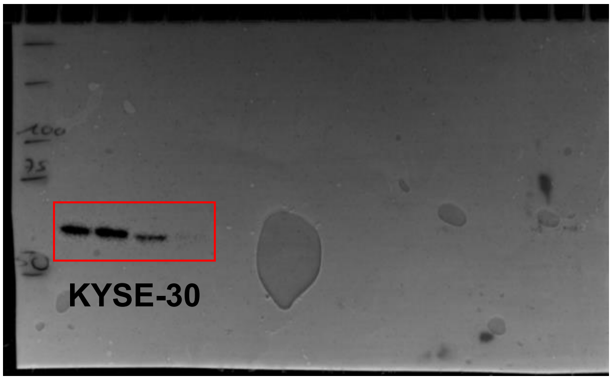


cGAS

Exposure 4sum + marker

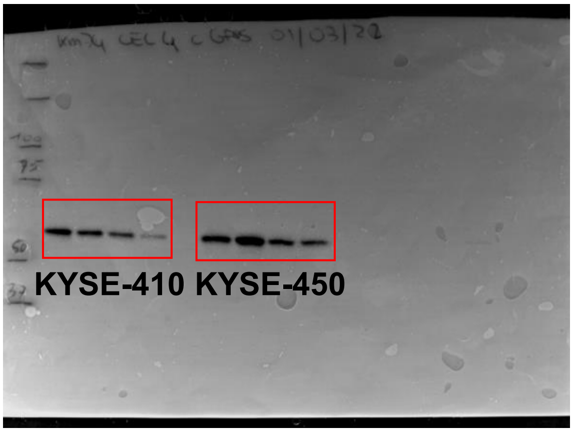


Exposure 8 + marker

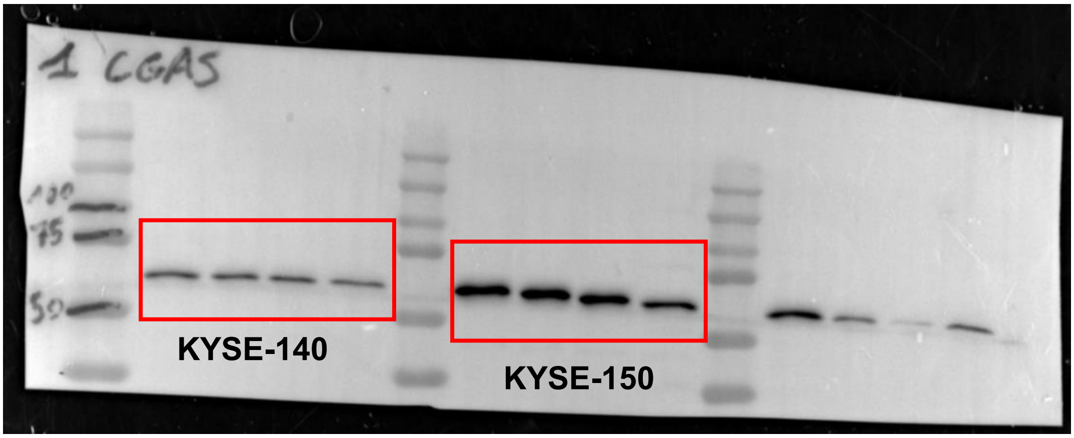


cGAS

Exposure 7 + marker

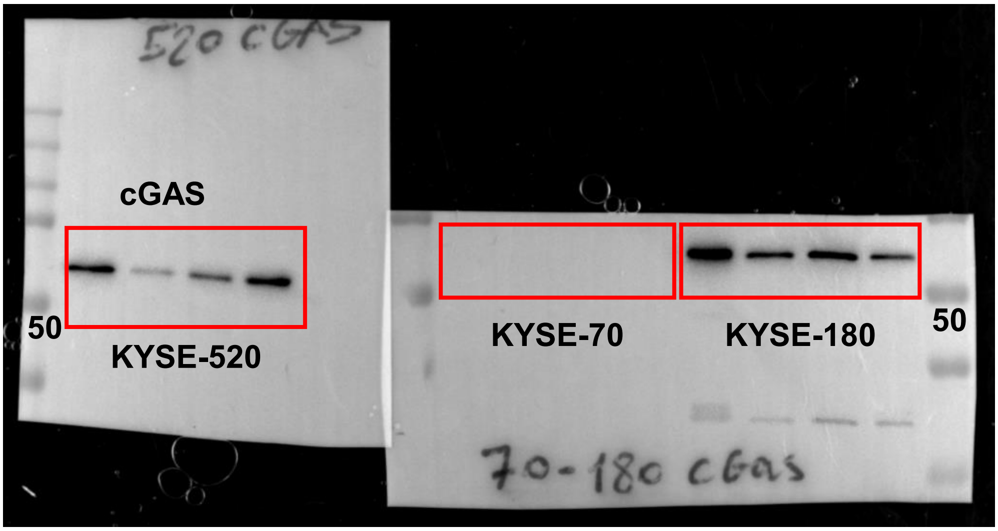


cGAS

**Exposure 7 + marker**

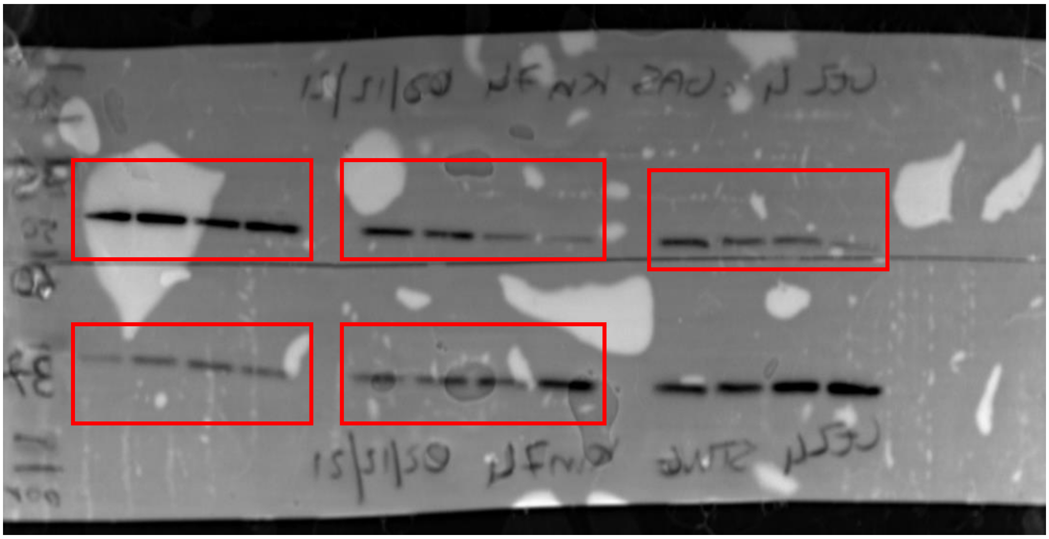

cGAS

STING

KYSE-270 KYSE-510 COLO-680N

**Exposure 5sum + marker**

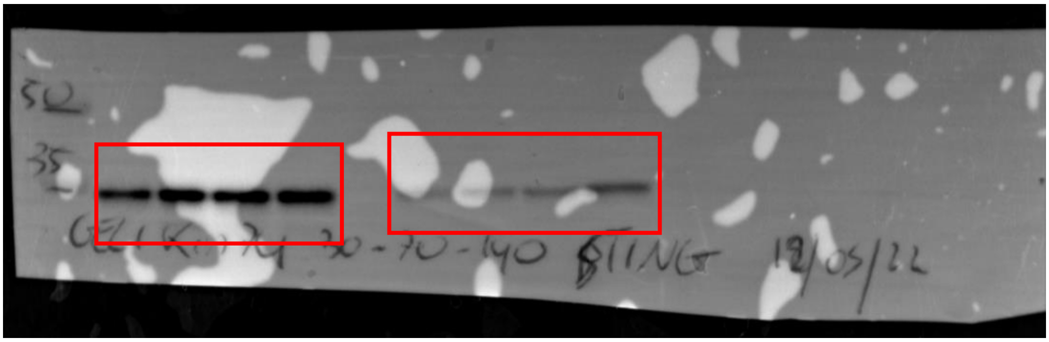

STING

KYSE-30 KYSE-70

**Exposure 6 + marker**

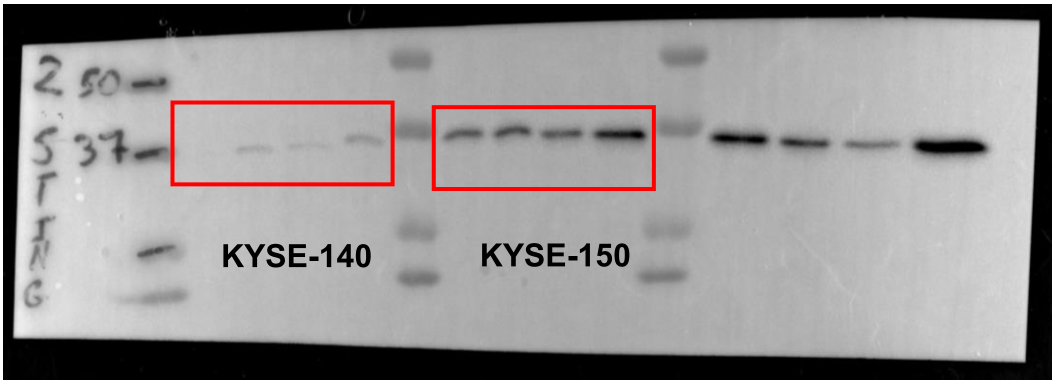


STING

**Exposure 6 + marker**

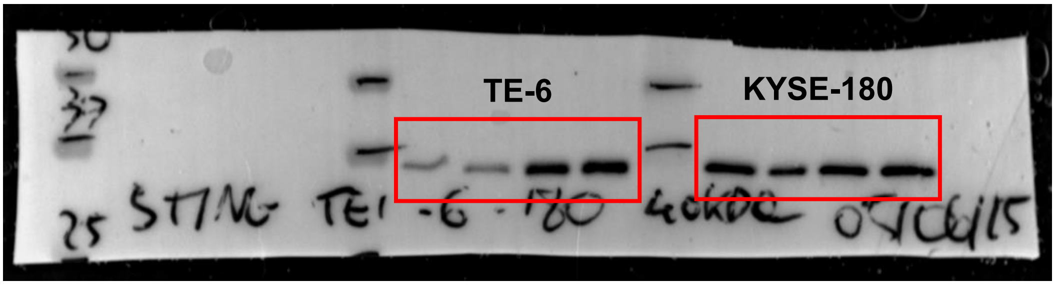


STING

**Exposure 5sum + marker**

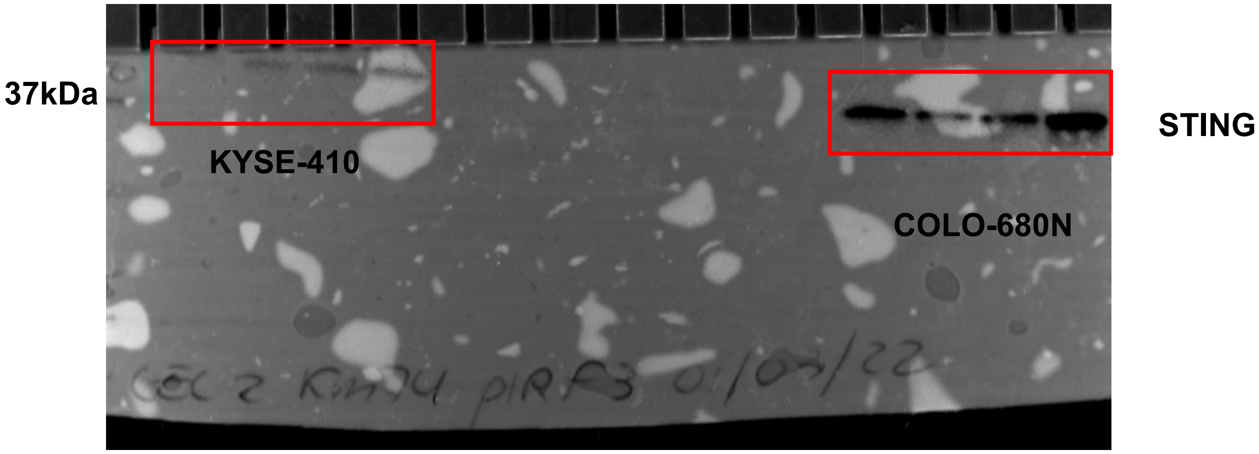


**Exposure 8 + marker**

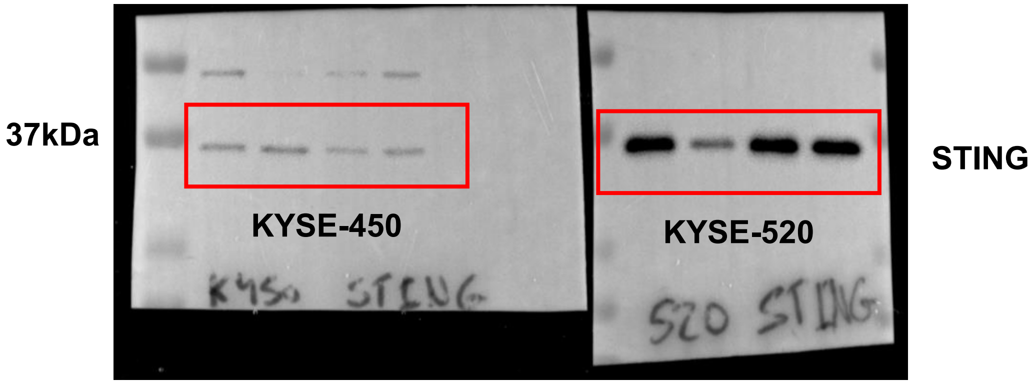


**Exposure 6 + marker**

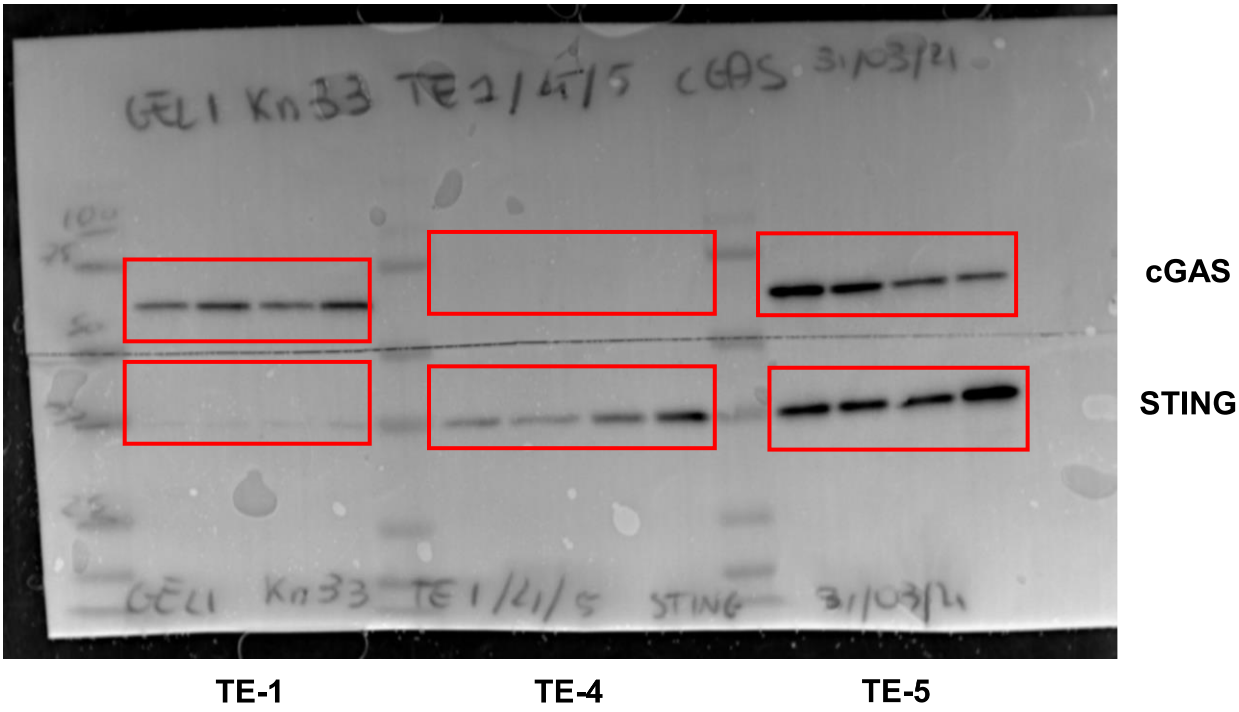

**Exposure 6 + marker**

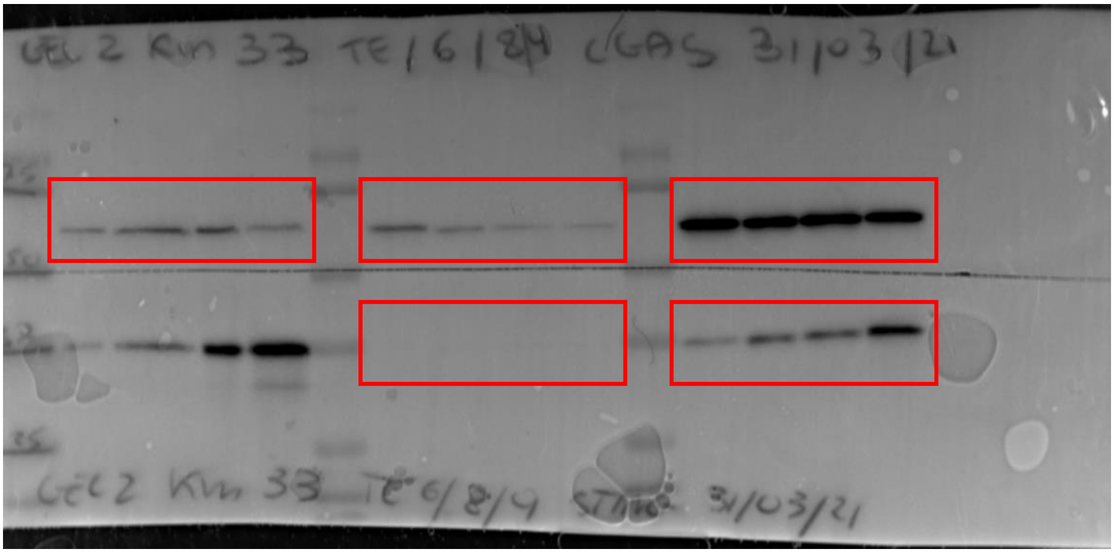

cGAS

STING

TE-6 TE-8 TE-9

**Exposure 7sum + marker**

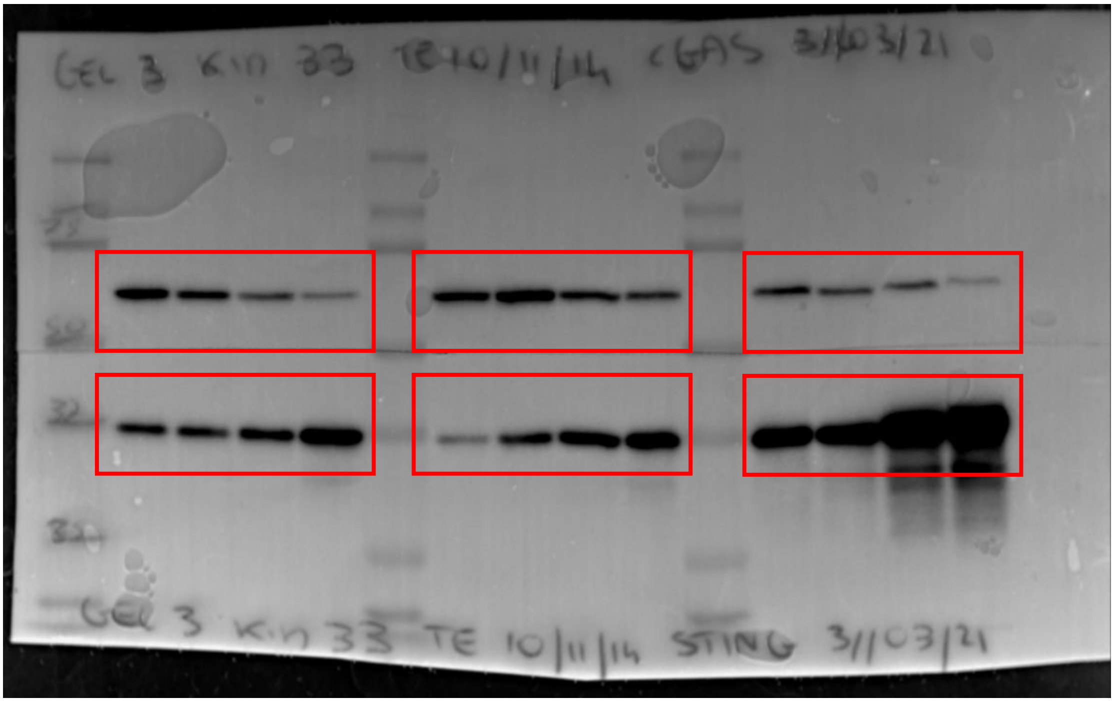

cGAS

STING

TE-10 TE-11 TE-14

Exposure 8sum + marker

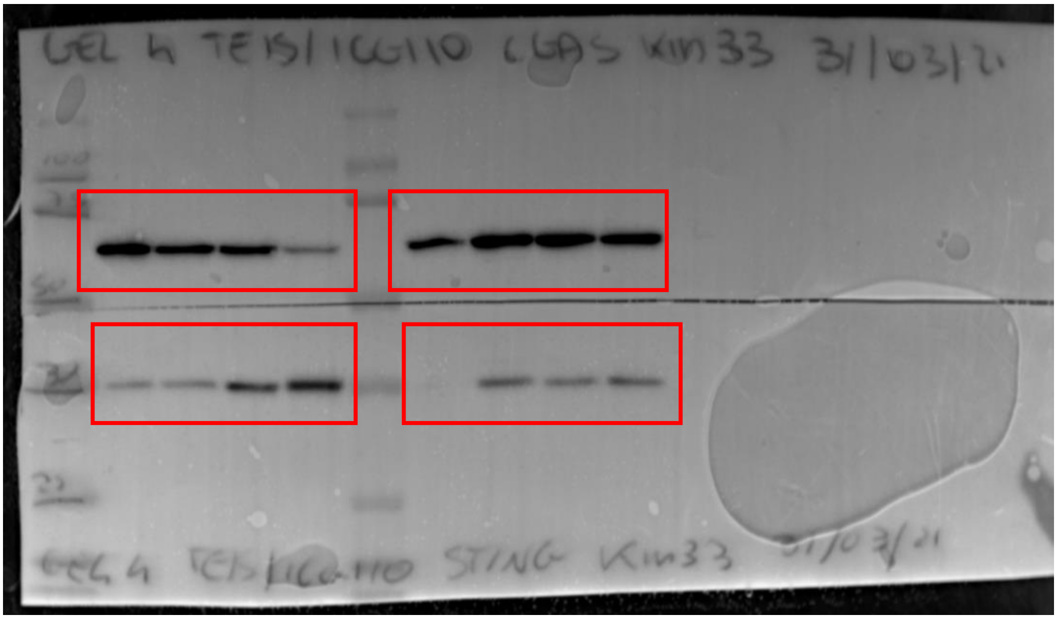

cGAS

STING

TE-15 ECGI-10

Exposure 7sum + marker

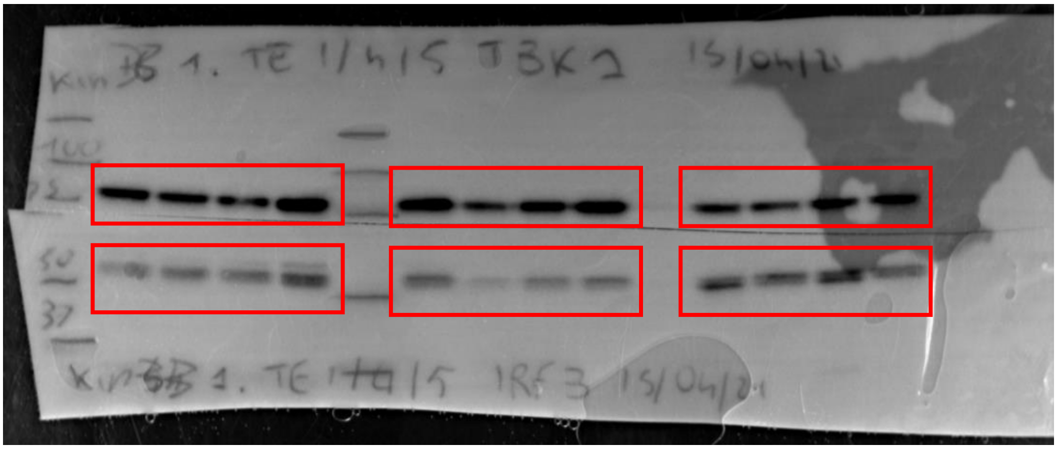

TBK1

IRF3

TE-1 TE-4 TE-5

Exposure 6sum + marker

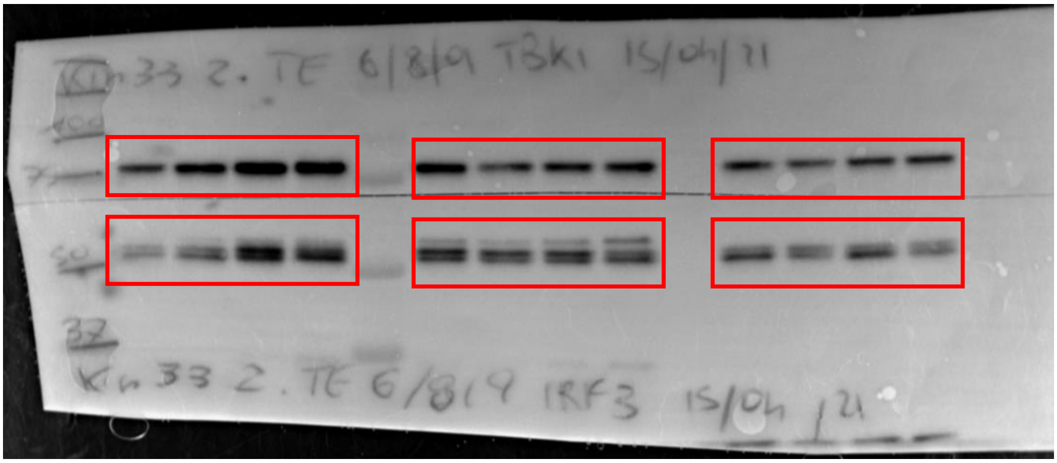

TBK1

IRF3

TE-6 TE-8 TE-9

**Exposure 7 + marker**

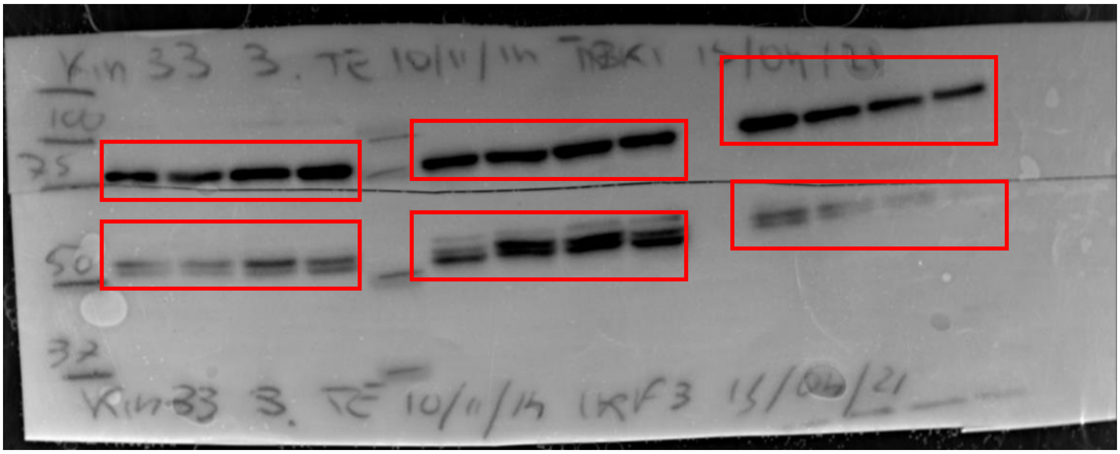

TBK1

IRF3

TE-10 TE-11 TE-14

**Exposure 6 + marker**

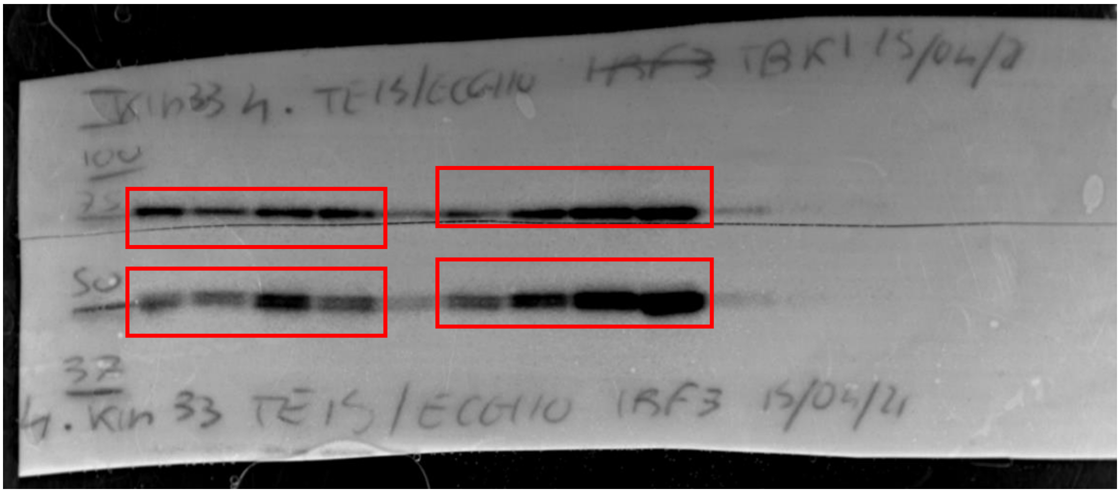

TBK1

IRF3

TE-15 ECGI-10

**Exposure 8 + marker**

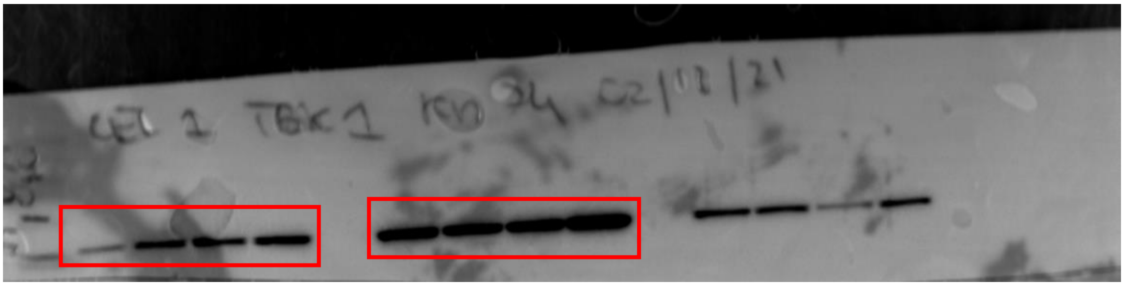

TBK1

KYSE-140 KYSE-150

**Exposure 7sum + marker**

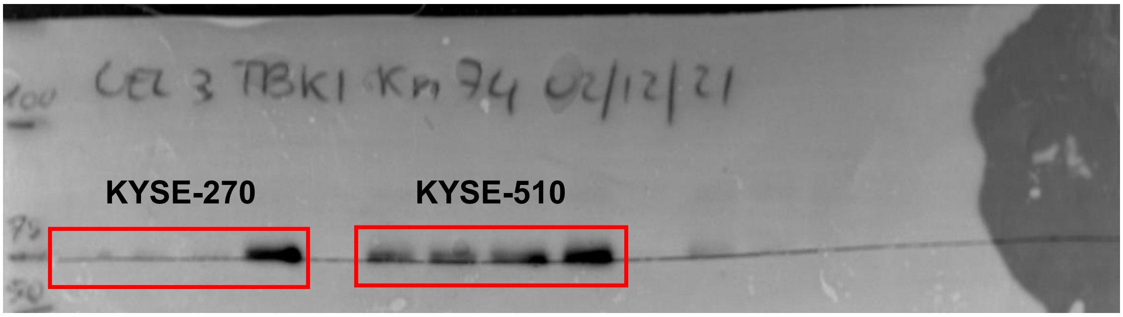


TBK1

Exposure 7sum + marker

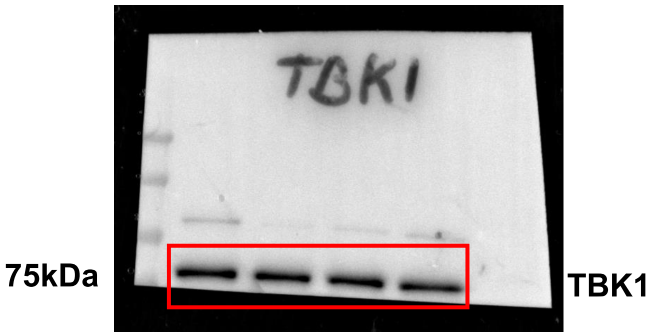


Exposure 5sum + marker

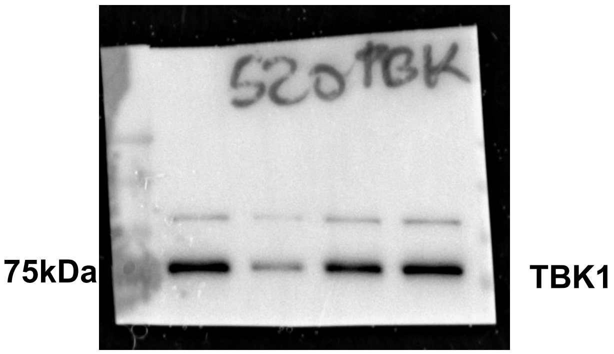


Exposure 7sum + marker

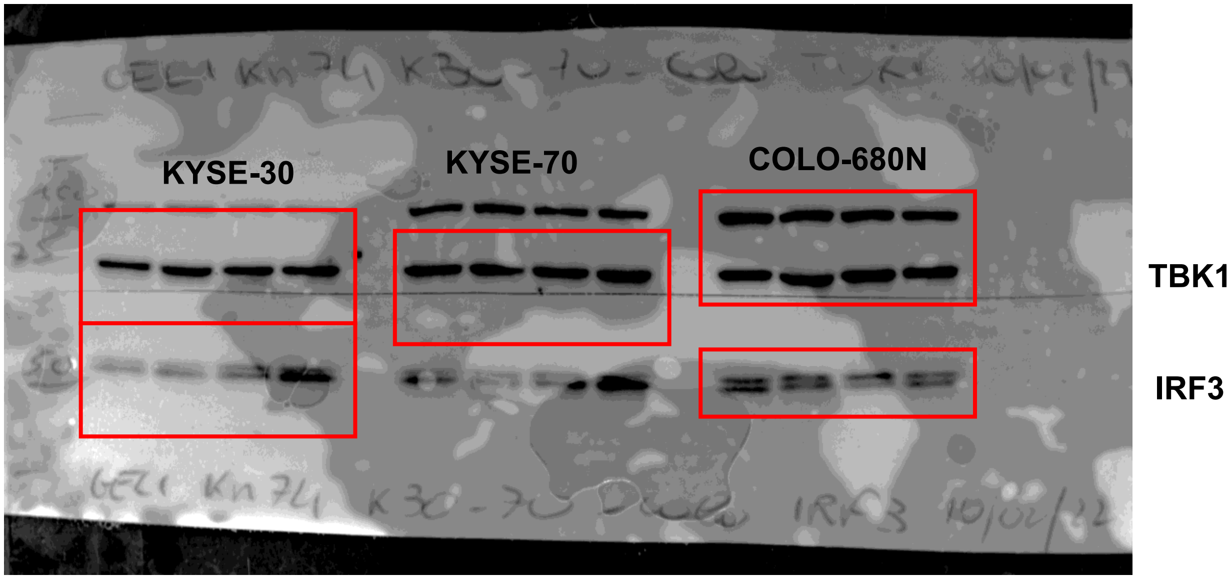


Exposure 5sum + marker

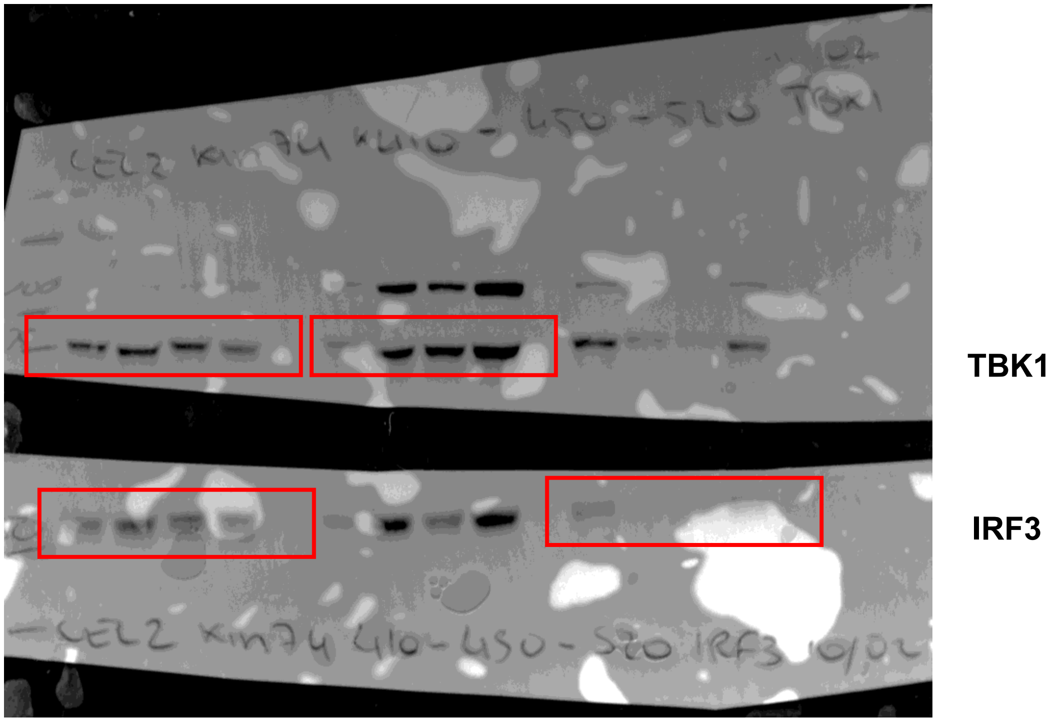

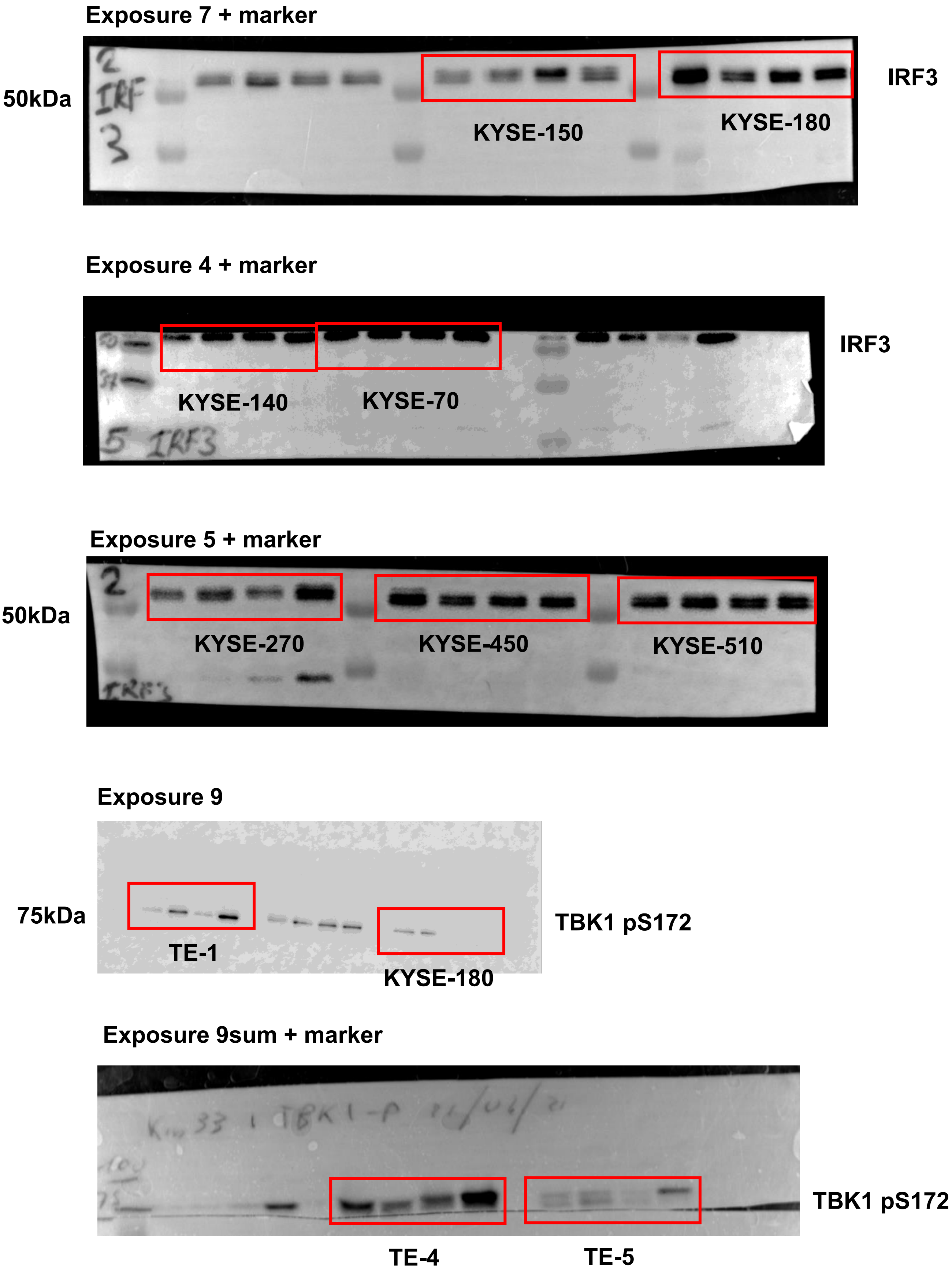
Exposure 7 + marker
50kDa
IRF3
KYSE-150
KYSE-180
Exposure 4 + marker
IRF3
KYSE-140
KYSE-70
Exposure 5 + marker
50kDa
KYSE-270
KYSE-450
KYSE-510
Exposure 9
75kDa
TE-1
KYSE-180
TBK1 pS172
Exposure 9sum + marker
TBK1 pS172
TE-4
TE-5

Exposure 9 + marker

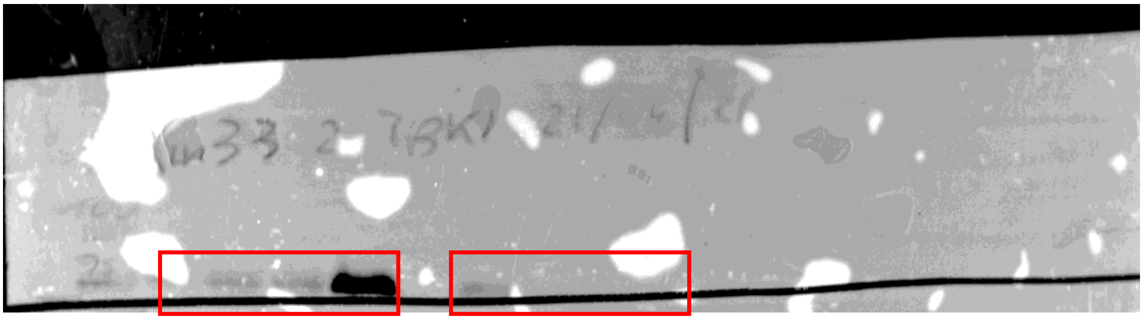

TBK1 pS172

TE-6 TE-8

Exposure 9 + marker

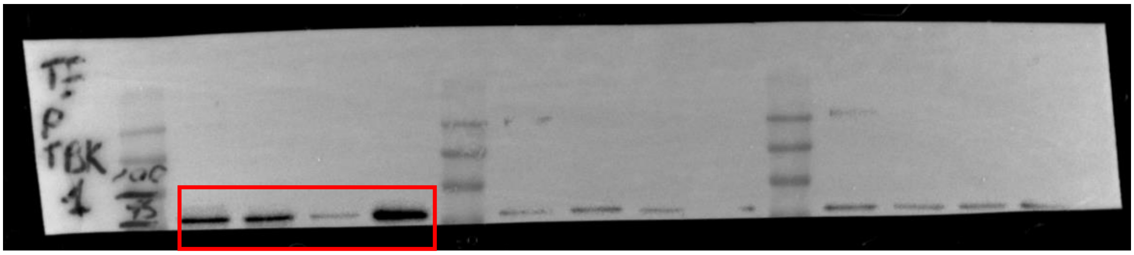

TBK1 pS172

TE-9

Exposure 7 + marker

75kDa

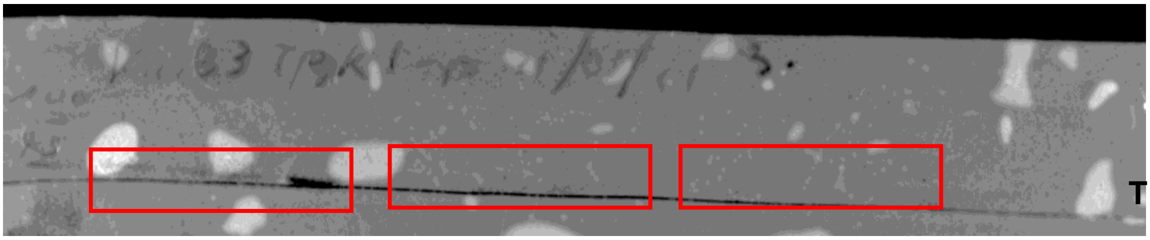

TBK1 pS172

TE-10 TE-11 TE-14

Exposure 9 + marker

75kDa

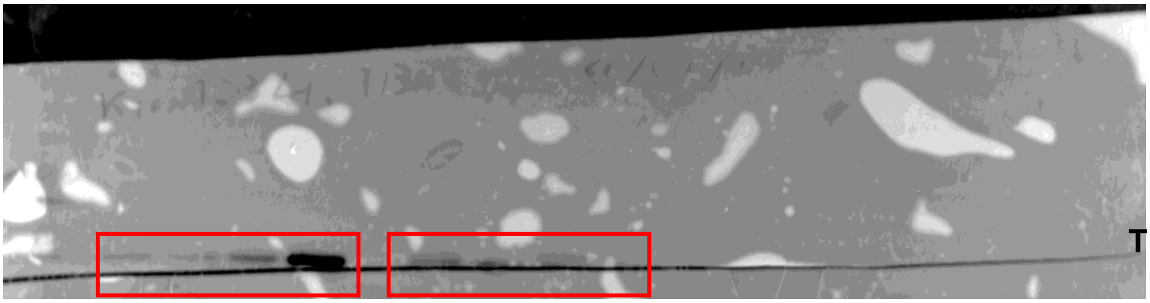

TBK1 pS172

TE-15 ECGI-10

Exposure 9 + marker KYSE-150 KYSE-510

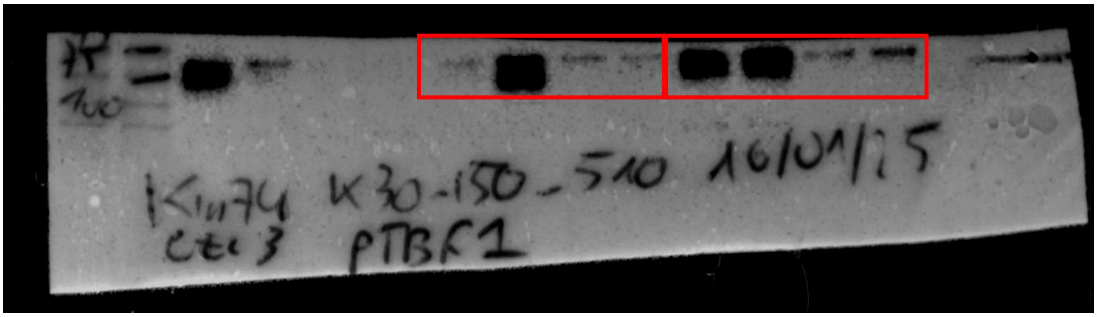

TBK1 pS172

Exposure 10sum

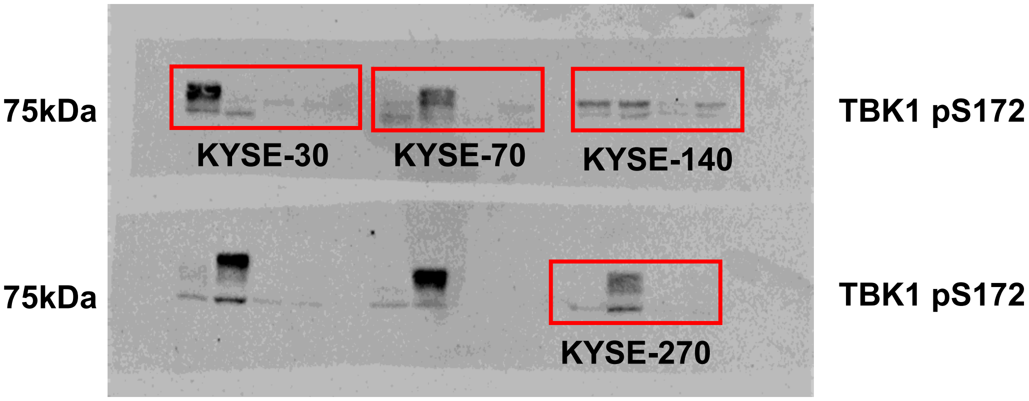


Exposure 9sum +marker

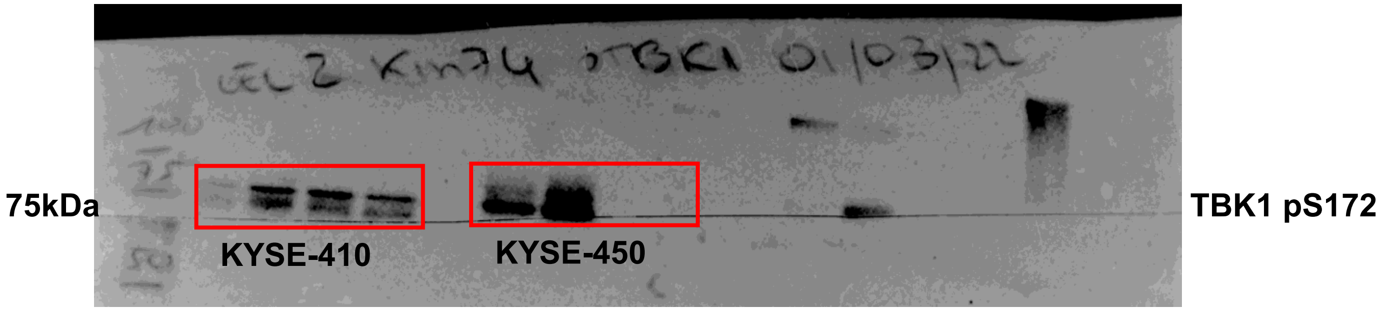


Exposure 10 +marker

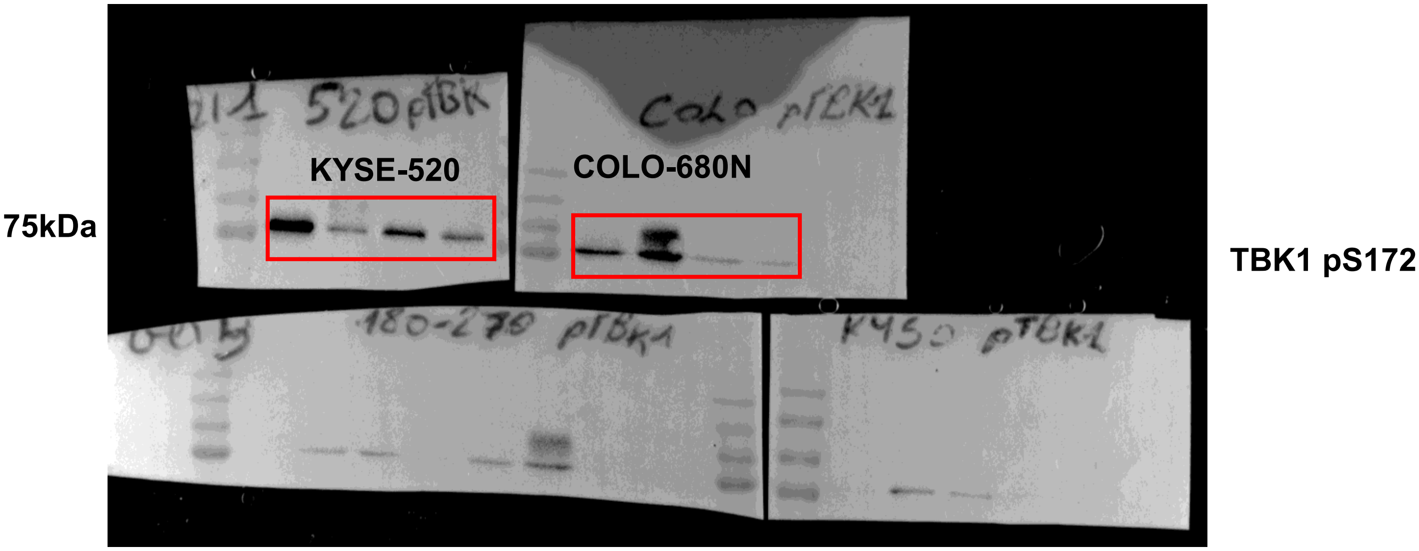


Exposure 7 +marker

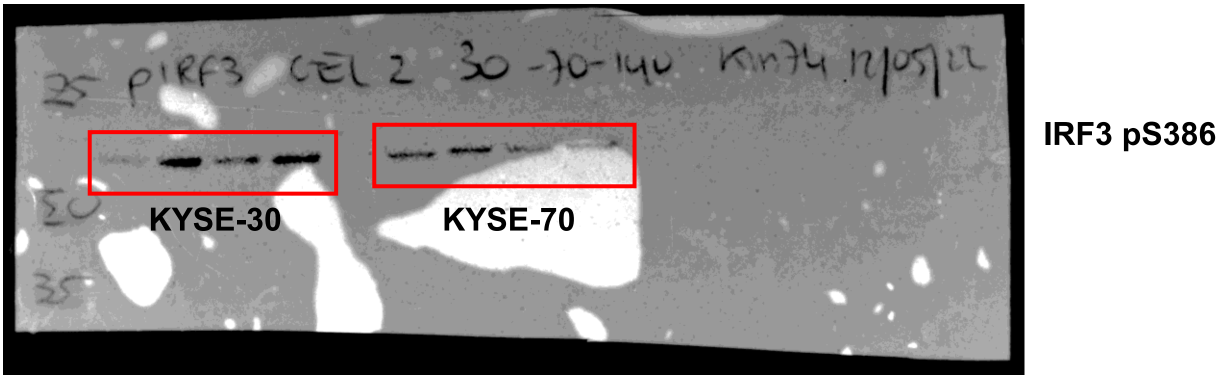

**Exposure 7 + marker**

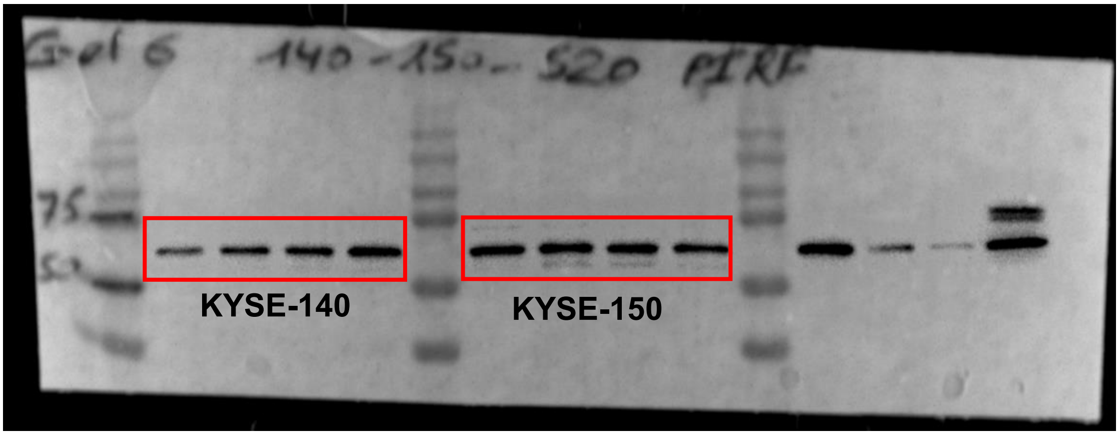


IRF3 pS386

**Exposure 8 + marker**

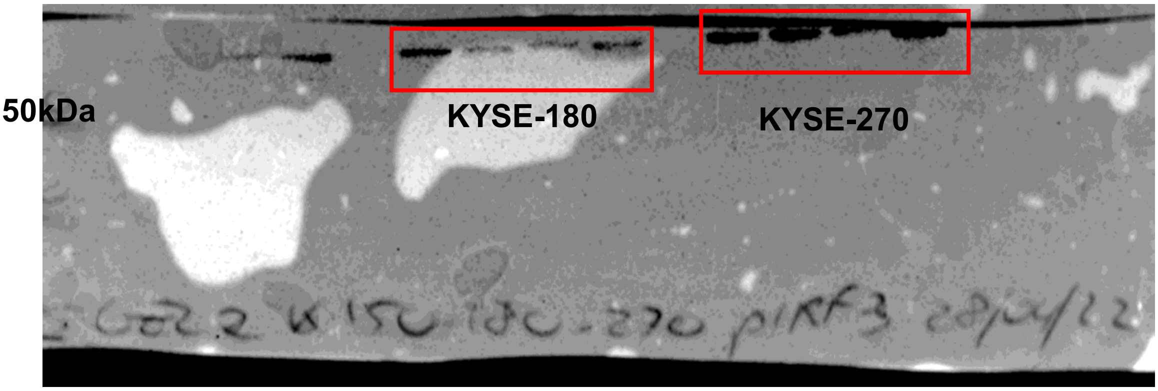


IRF3 pS386

**Exposure 9sum + marker**

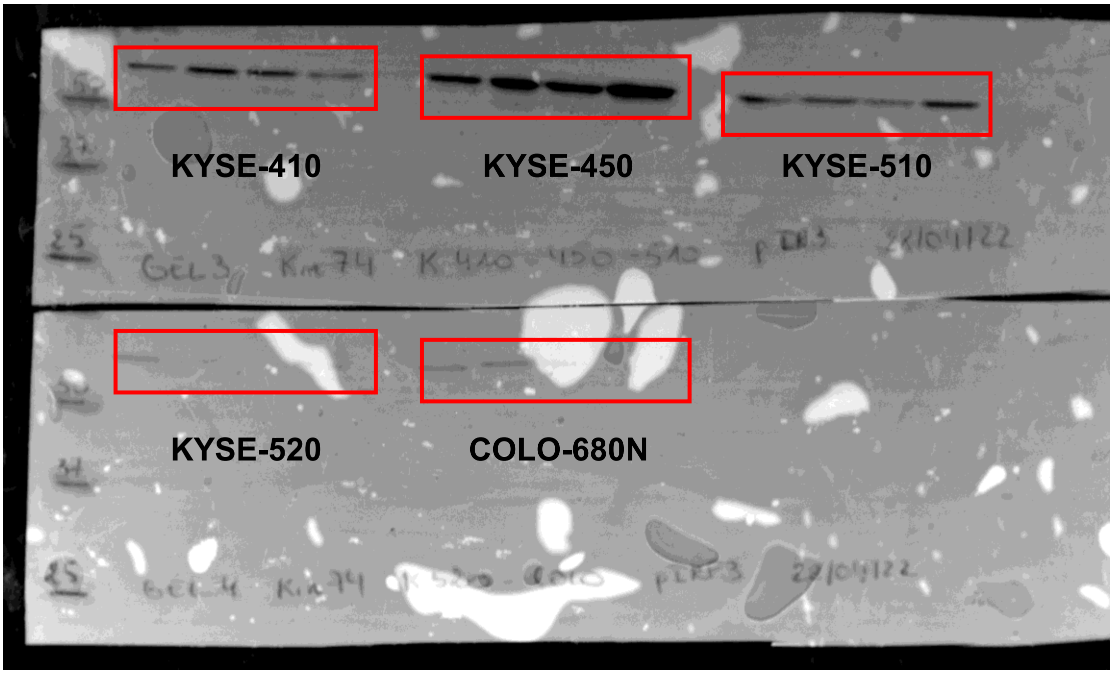


IRF3 pS386

IRF3 pS386

Exposure 9sum + marker

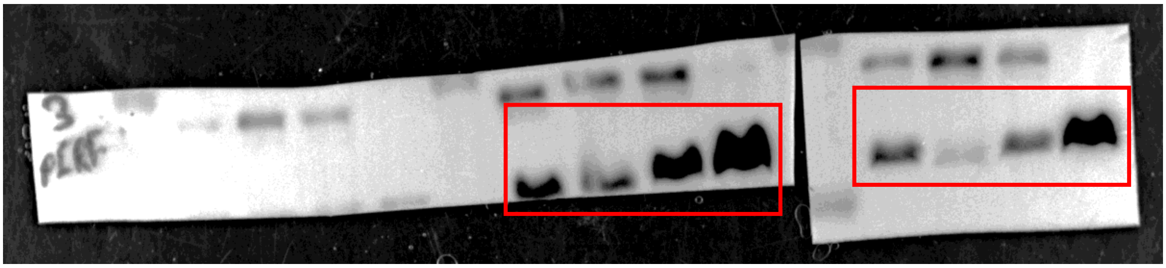


Exposure 7sum

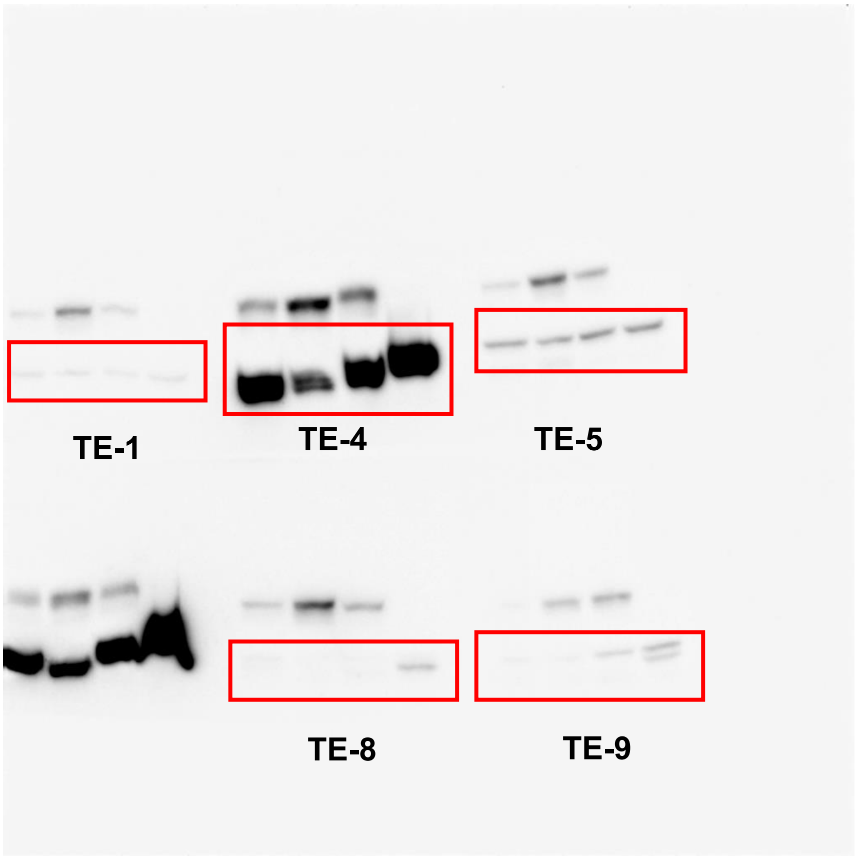


Exposure 7sum + marker

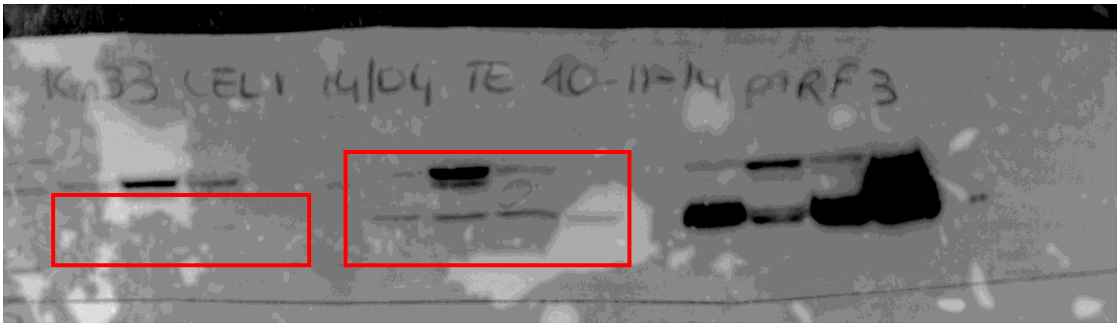

Exposure 9sum + marker

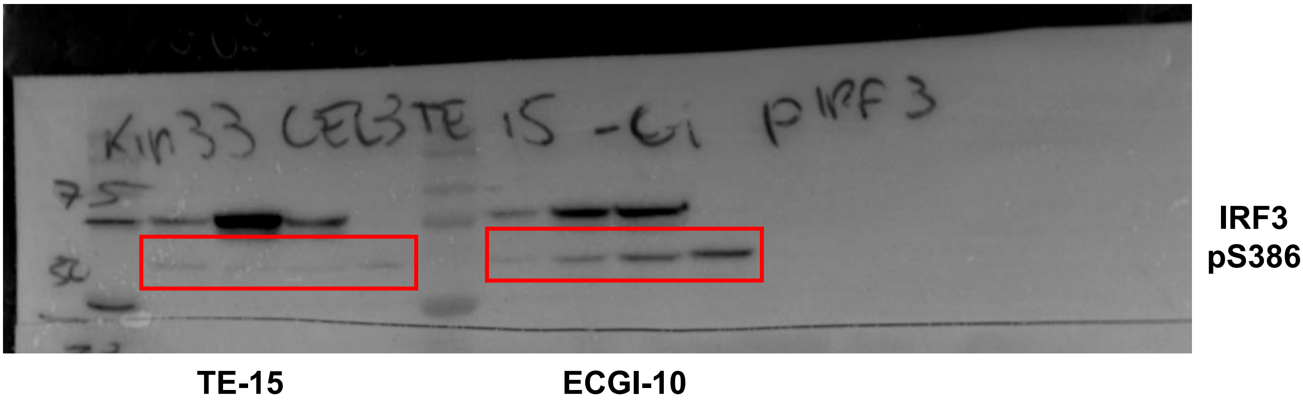

**Supplementary Figure 2 – 2D CDK4**

KYSE-30 exposure 2sum

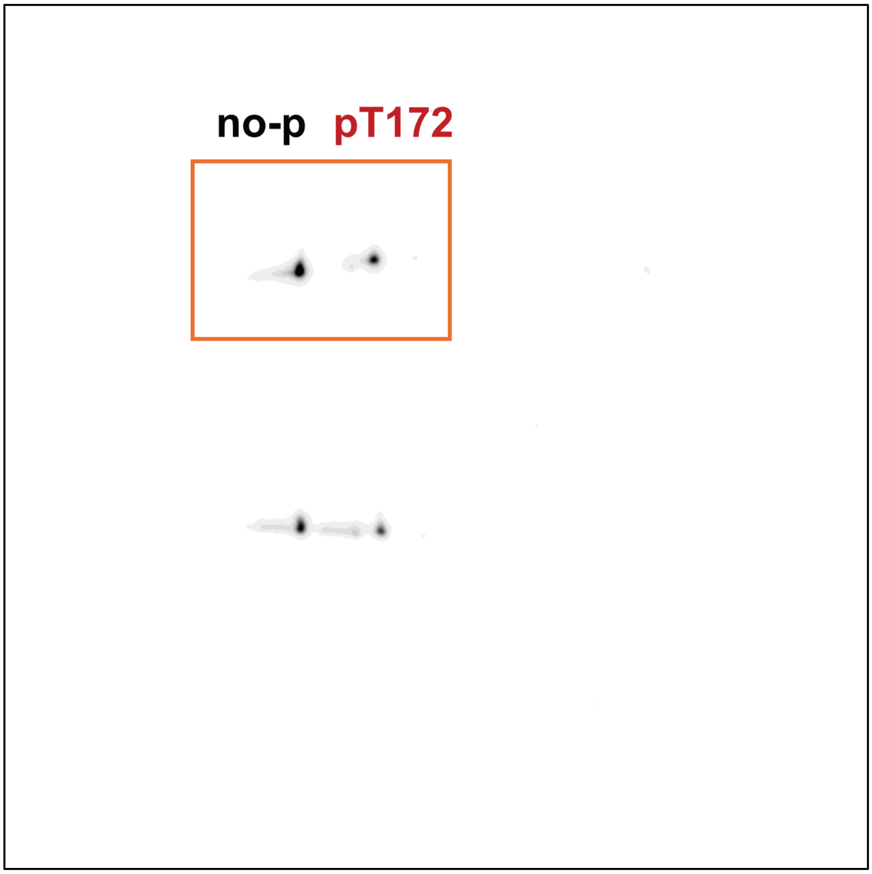


KYSE-70 exposure 2

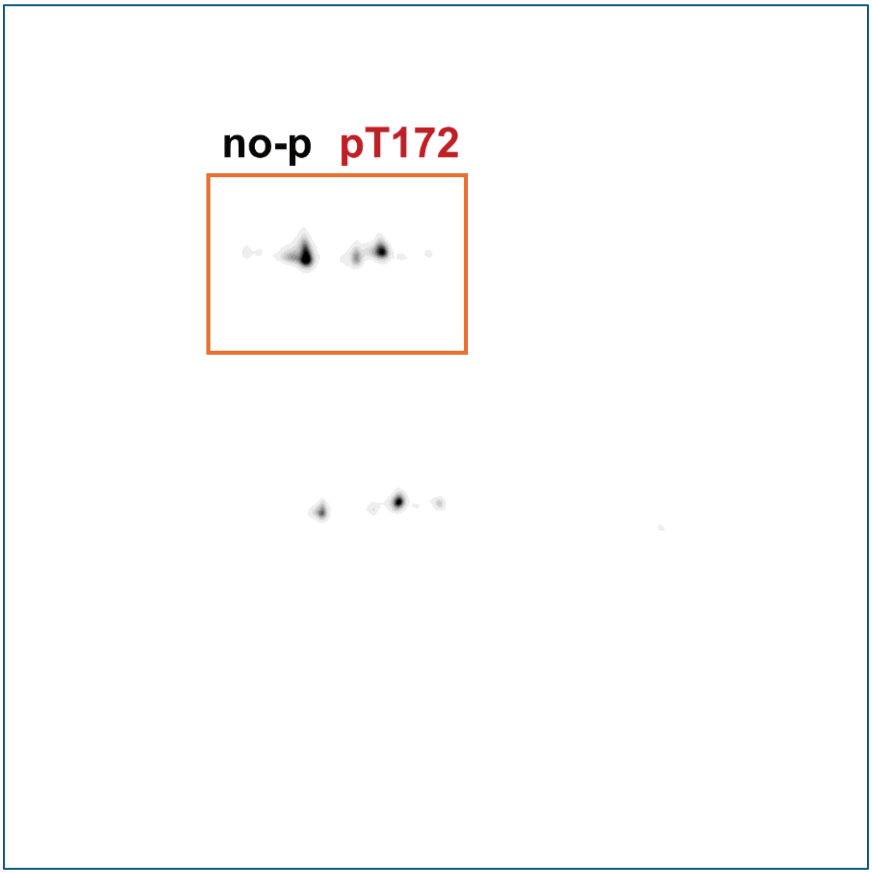

KYSE-140 exposure 2sum

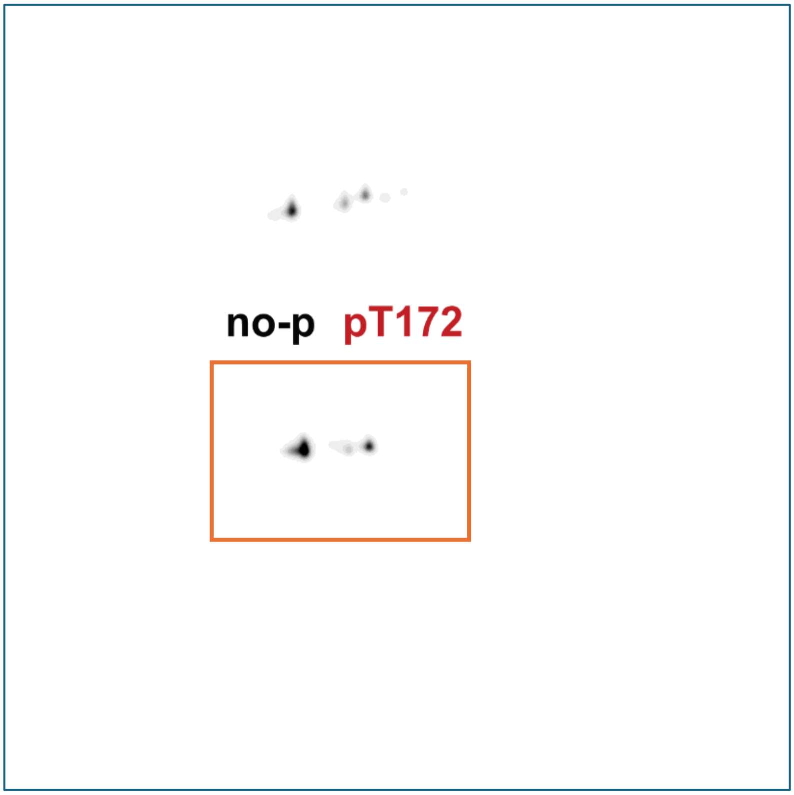


KYSE-150 exposure 2 sum

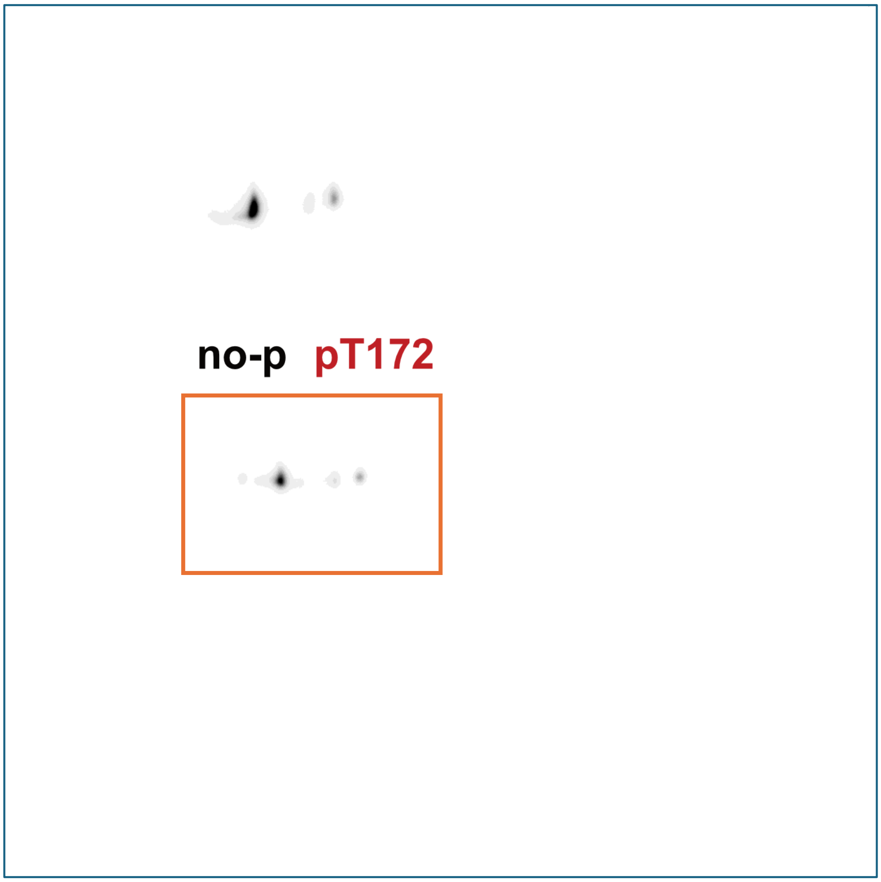

KYSE-180 exposure 2'sum

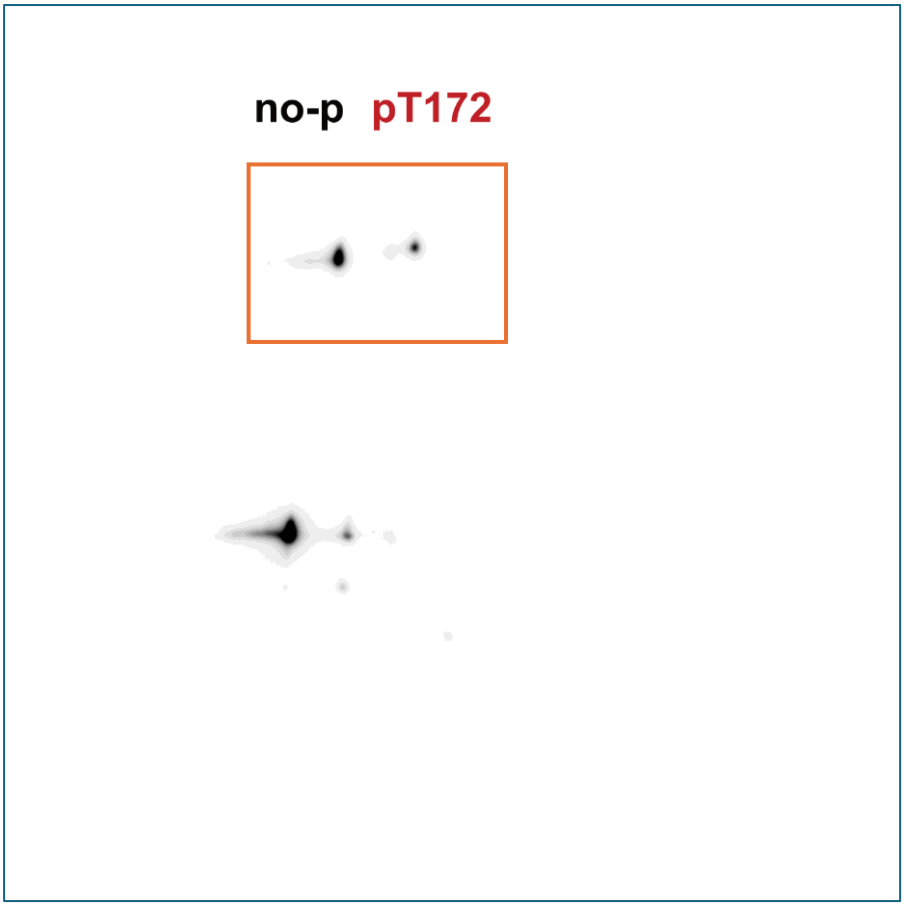


KYSE 270 exposure 2 sum

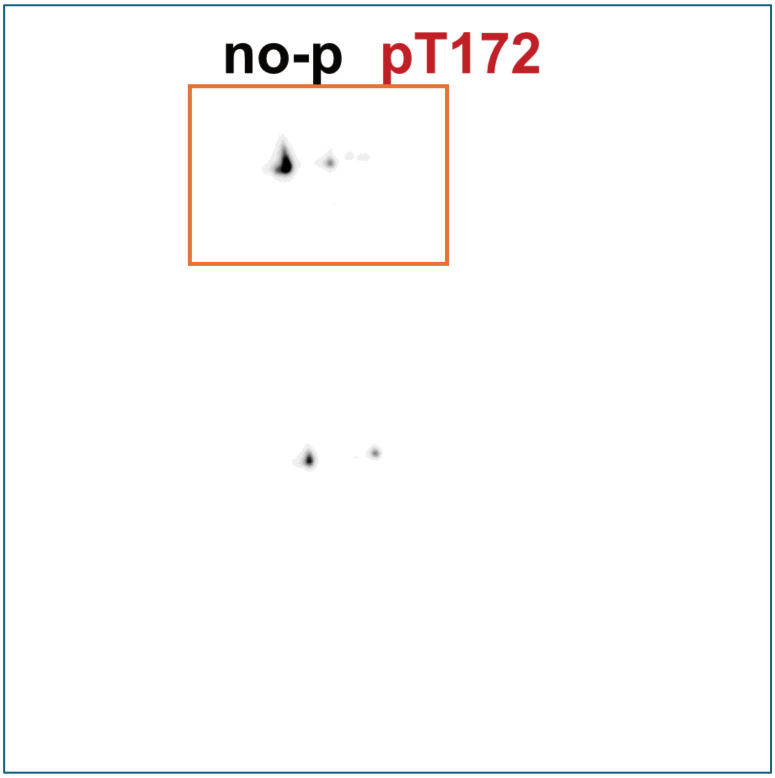

exposure 1

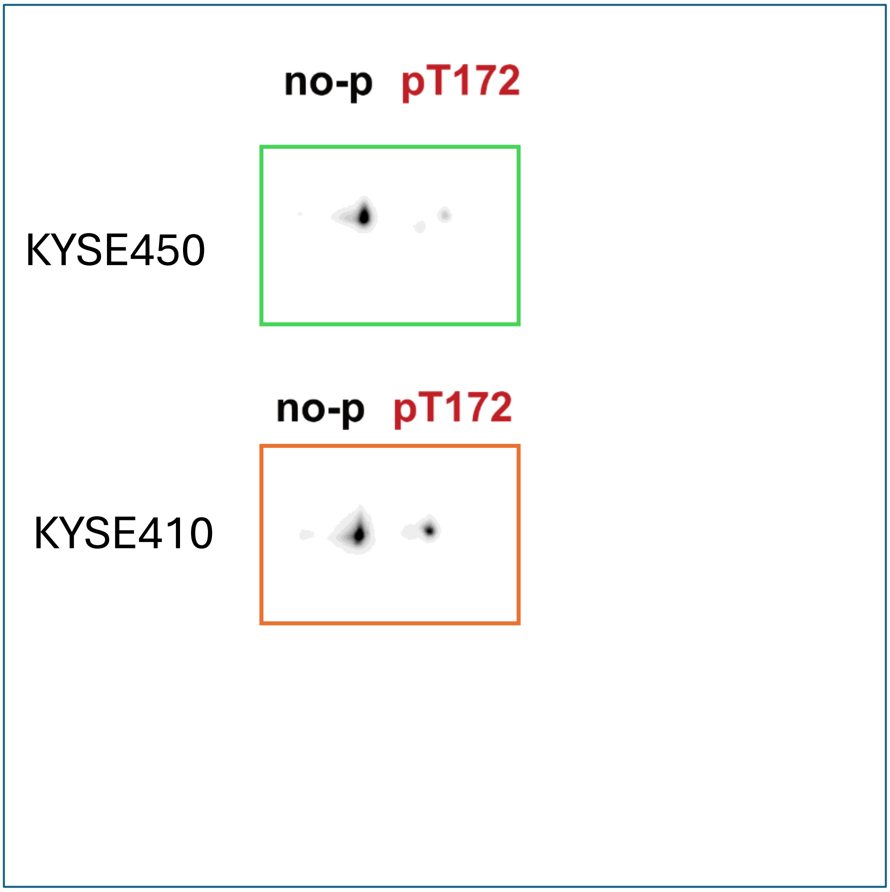

KYSE510 exposure 1

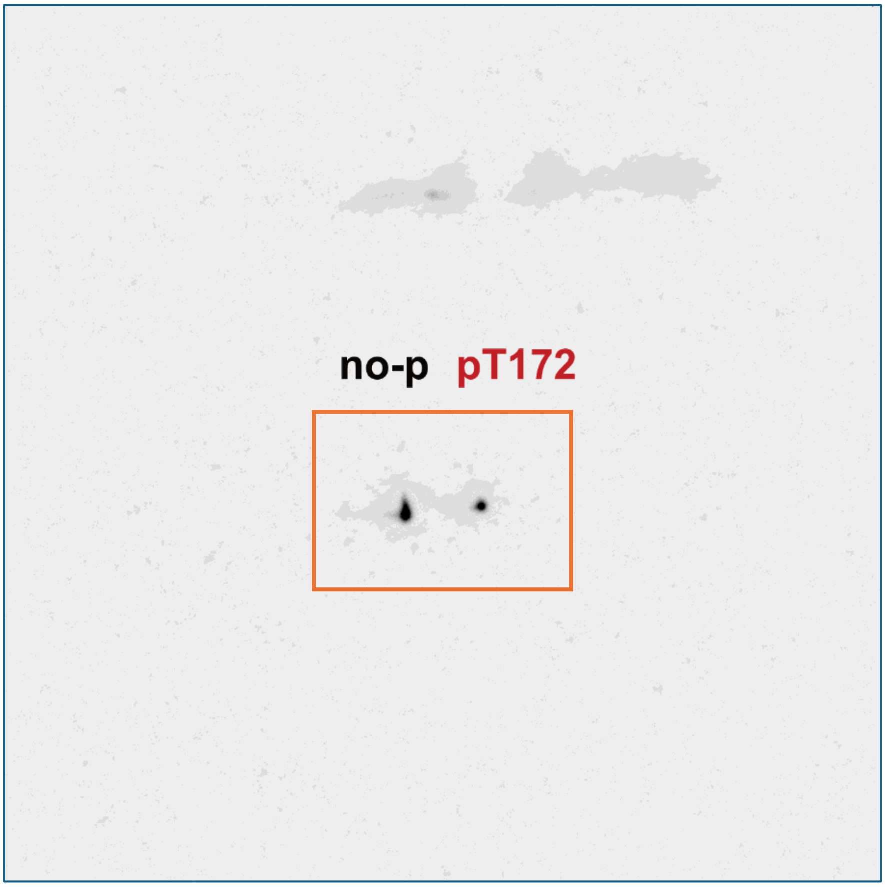


KYSE-520 exposure 2'sum

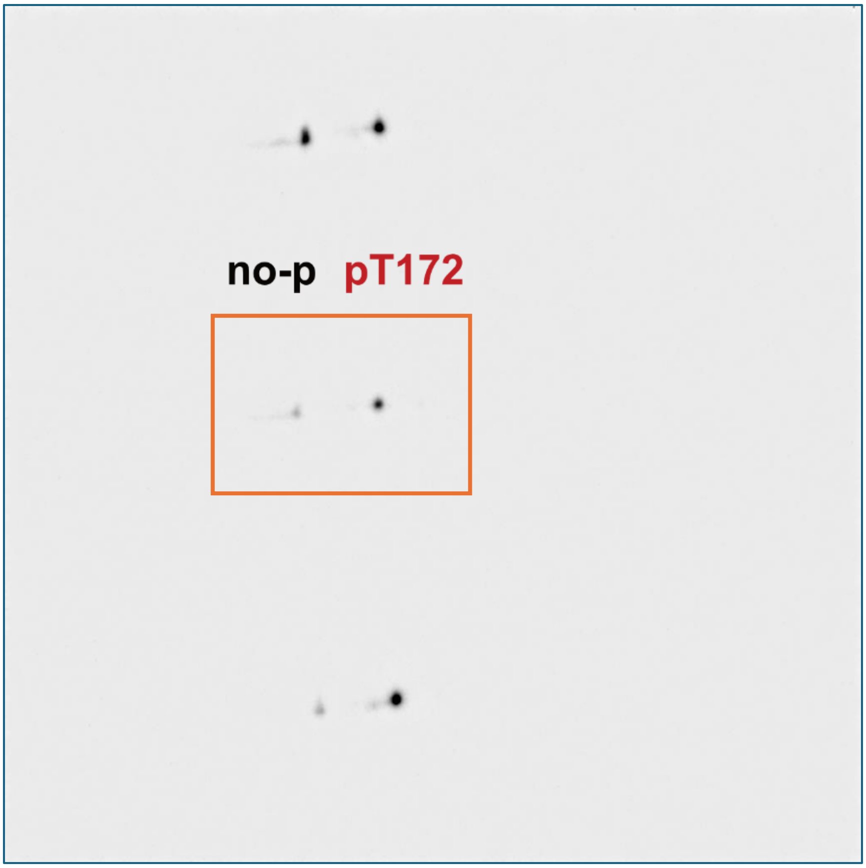

COLO680N exposure 2

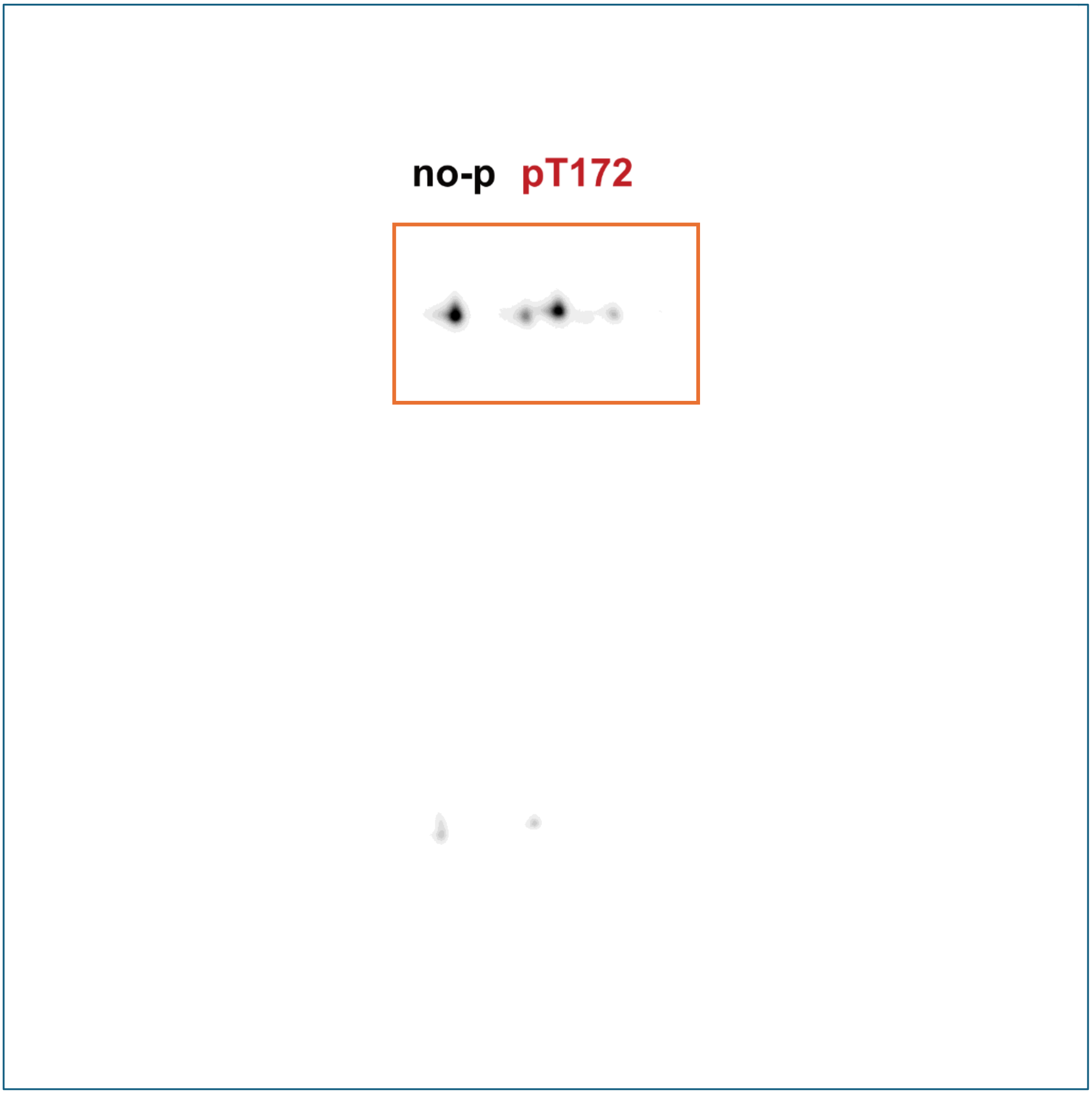

TE-1 exposure 5Sum

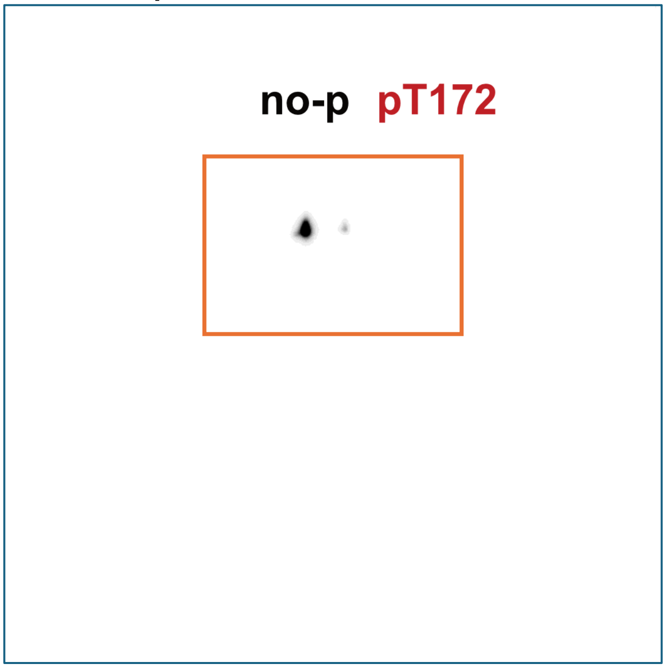


TE-4 exposure 8Sum

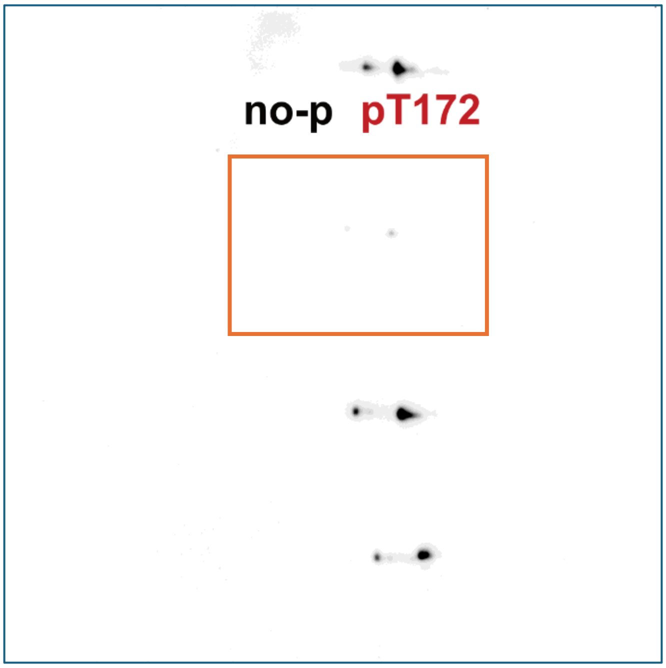

Exposure 9 sum

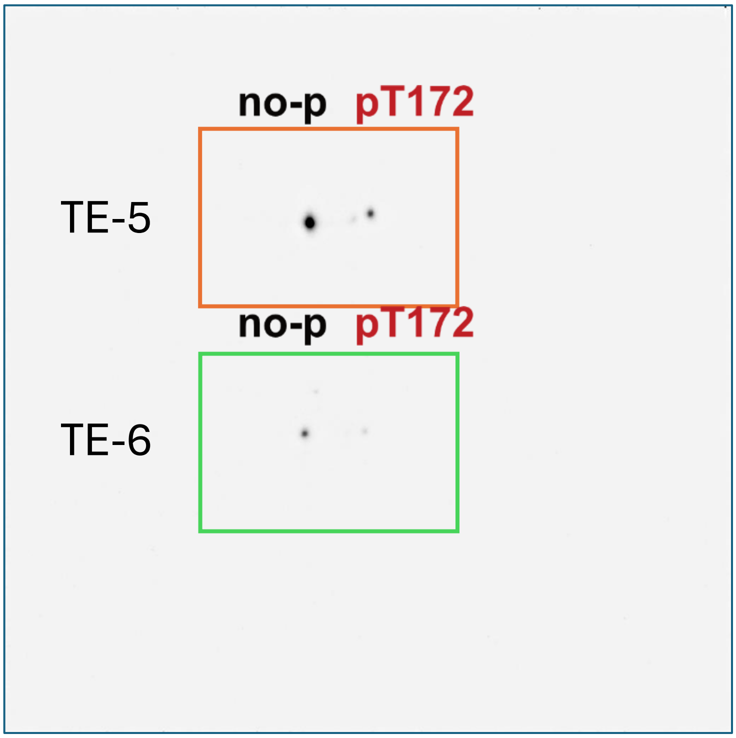

Exposure 8Sum

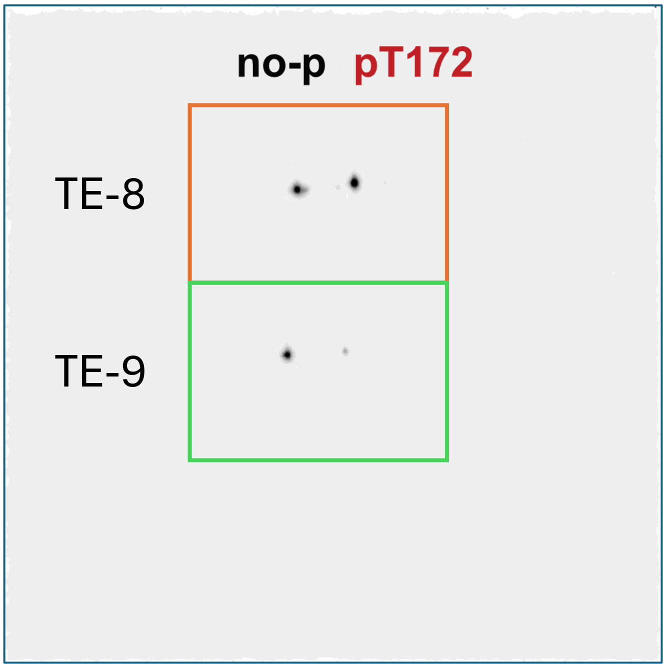

TE-10 exposure 8Sum

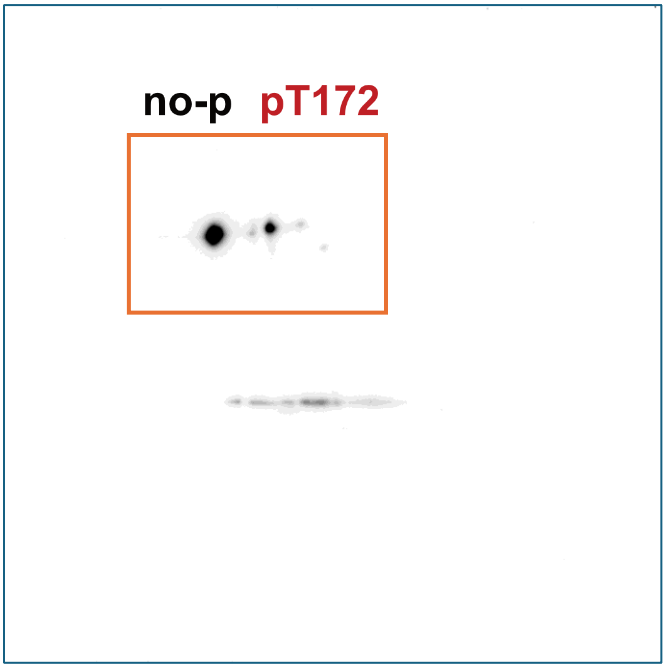


TE-11 2D exposure 5Sum

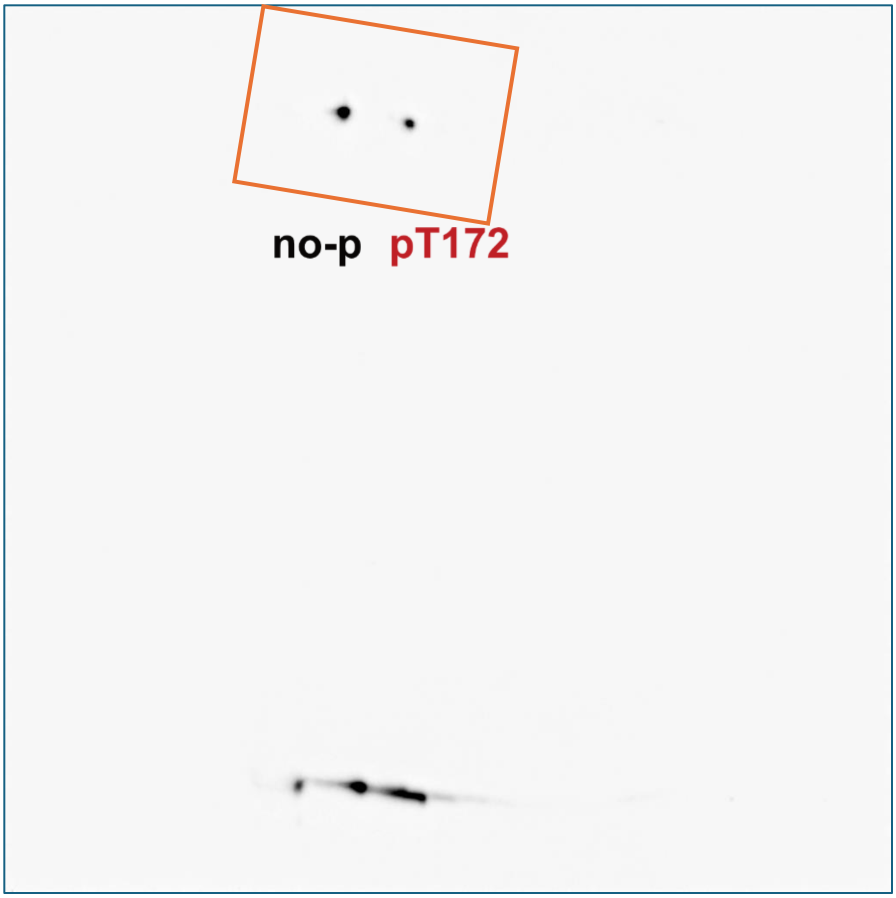

Exposure 7Sum

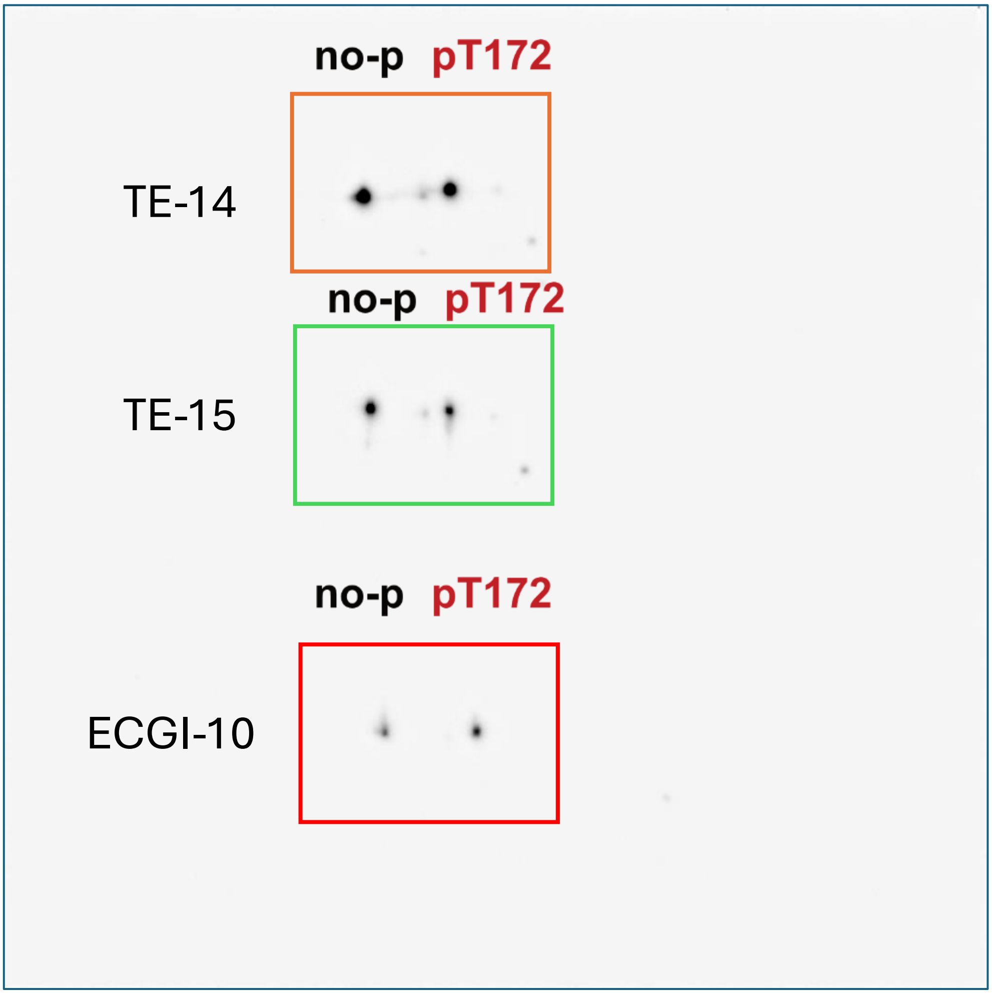

Fig S2A

MDA-MB231 MDA-MB468 HCC1806 KYSE-30 KYSE-70 KYSE-140 KYSE-150 KYSE-180 KYSE-270 KYSE-410 KYSE-450 KYSE-510 KYSE-520 COLO-680N MDA-MB231 MDA-MB468 HCC1806 TE-1 TE-4 TE-5 TE-6 TE-8 TE-9 TE-10 TE-11 TE-14 TE-15 ECGI-10

| | | |
|---|---|---|
| 1 | 2 | **Rb total** |
| 3 | 4 | **Rb pT807/T811** |
| 5 | 6 | **cyclin D1** |
| 7 | 8 | **cyclin D3** |
| 9 | 10 | **cyclin E** |
| 11 | 12 | **cyclin A** |
| 13 | 14 | **cyclin B1** |
| 15 | 16 | **CDK4** |
| 17 | 18 | **CDK6** |
| 19 | 20 | **CDK2** |
| 21 | 22 | **p16** |
| 23 | 24 | **p53** |
| 25 | 26 | **p63** |
| 27 | 28 | **HSP90** |

# Rb total

1- KYSE-COLO exposure 6

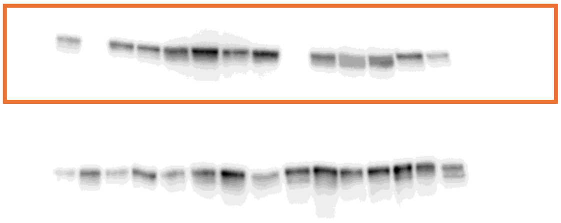

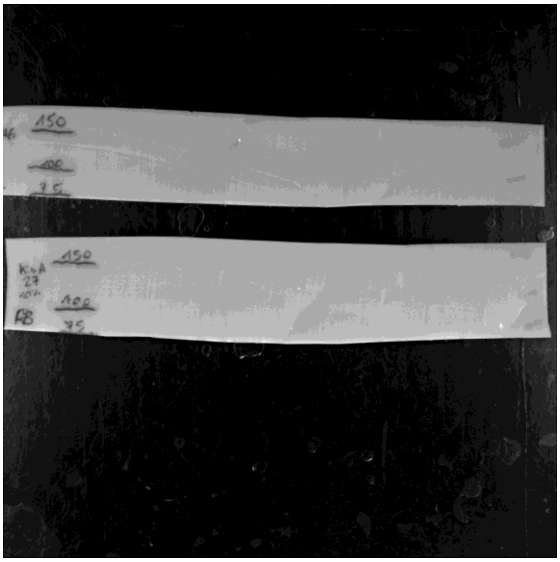

2 - TE exposure 6Sum

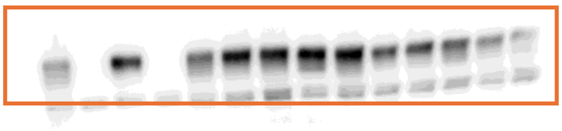

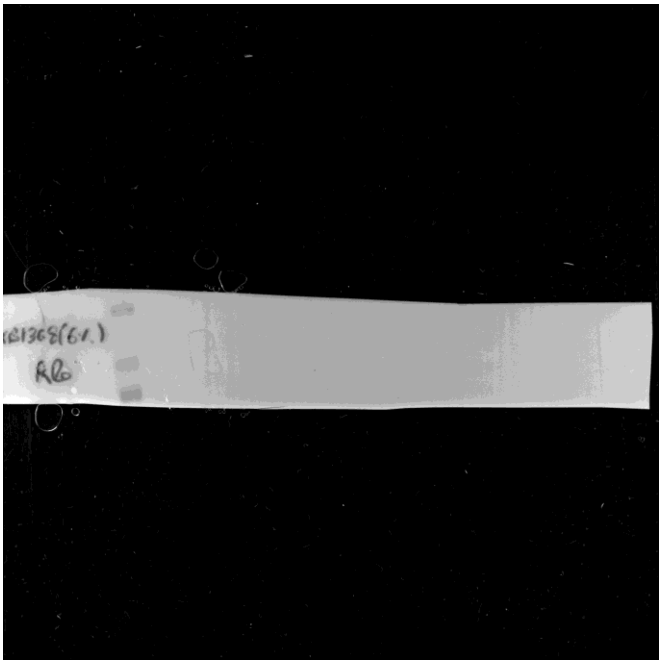

# Rb pT807/T811

3- KYSE –COLO exposure 1'Sum

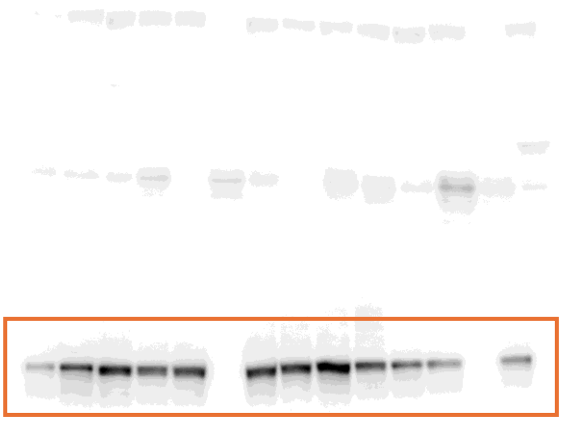

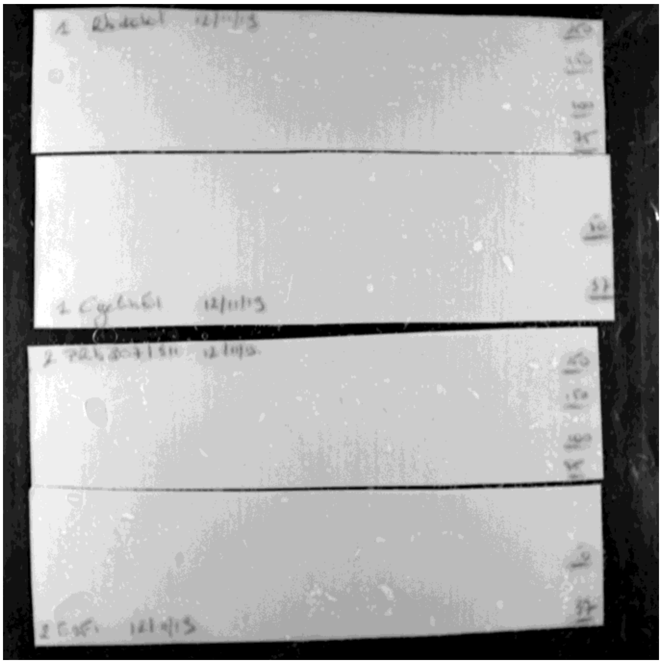

4- TE- exposure 3sum

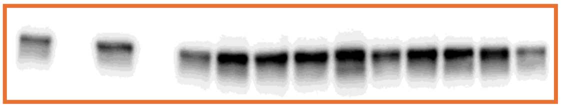

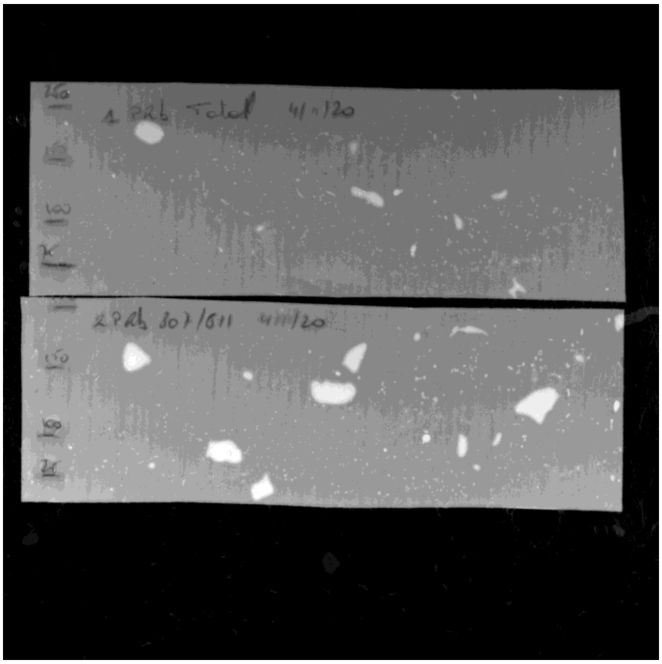

# cyclin D1

5- KYSE-COLO exposure 6'Sum

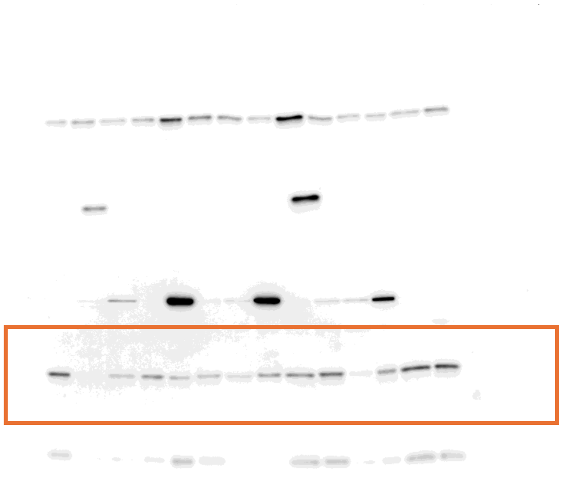

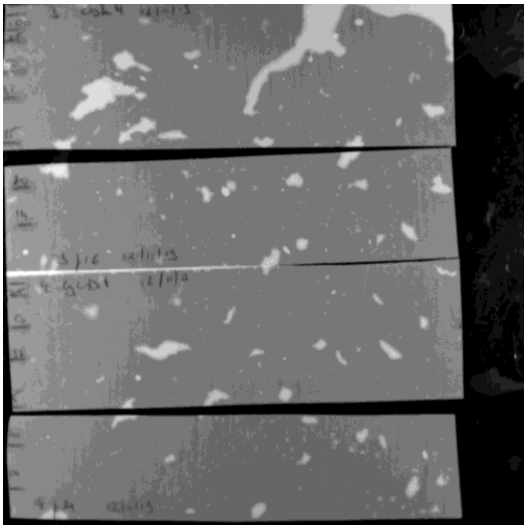

6- TE exposure 7sum

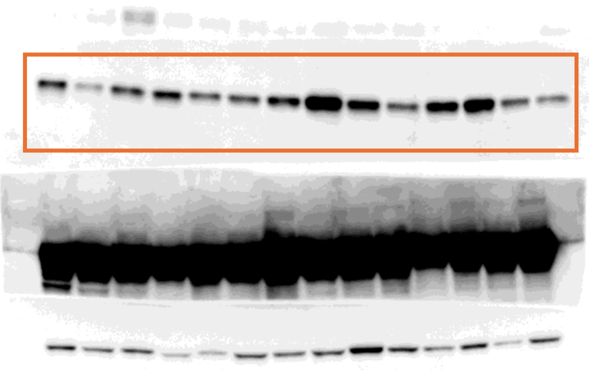

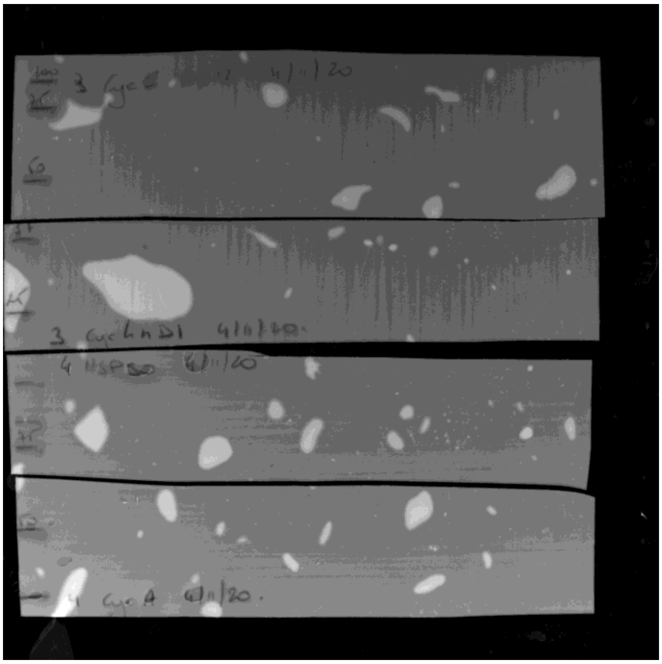

# cyclin D3

7- KYSE – exposure 4

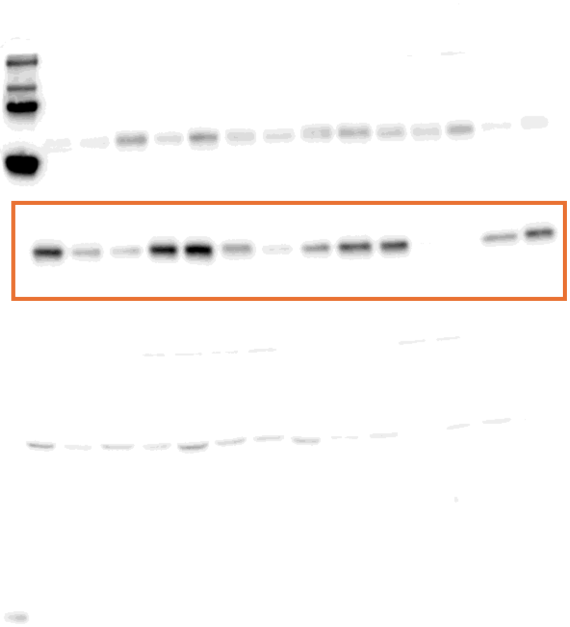

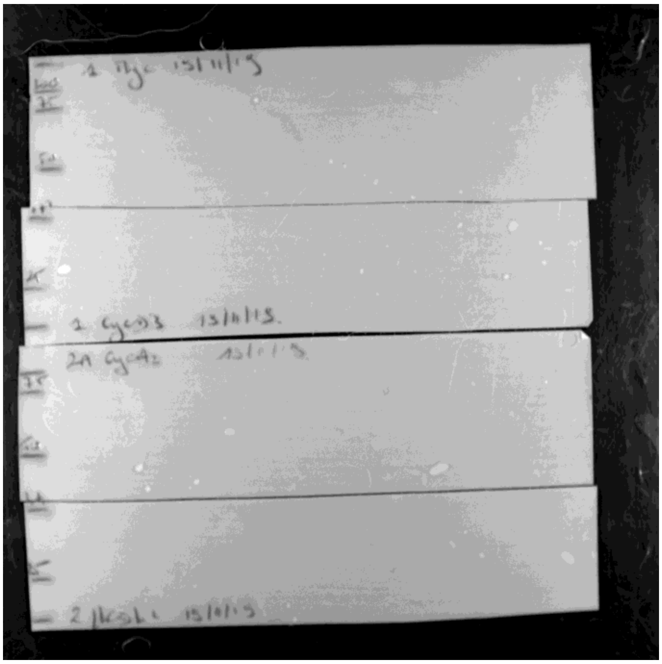

8- TE- exposure 8sum

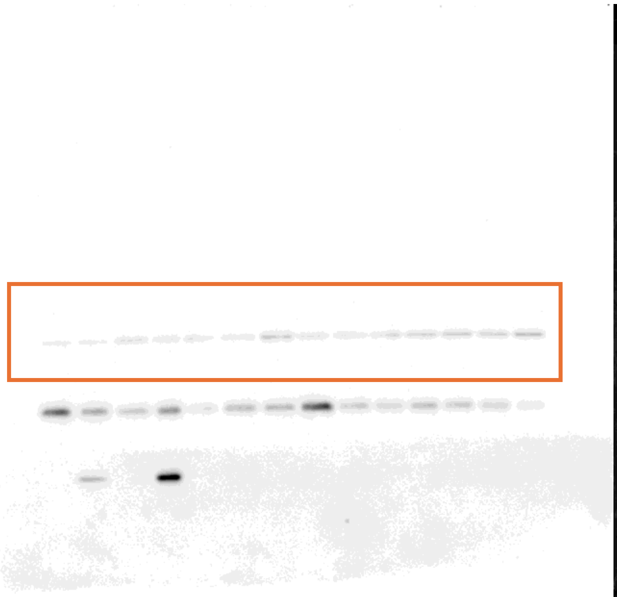

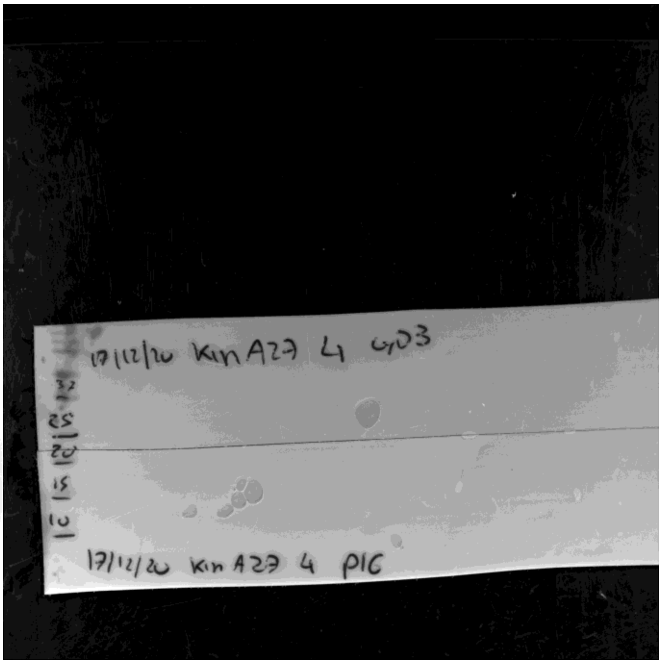

# cyclin E

9- KYSE- exposure 2'Sum

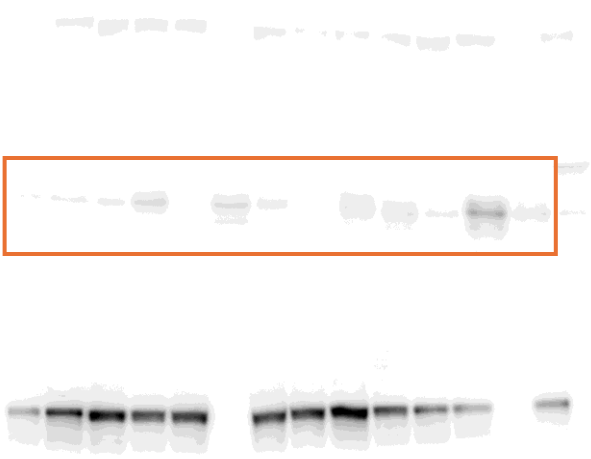

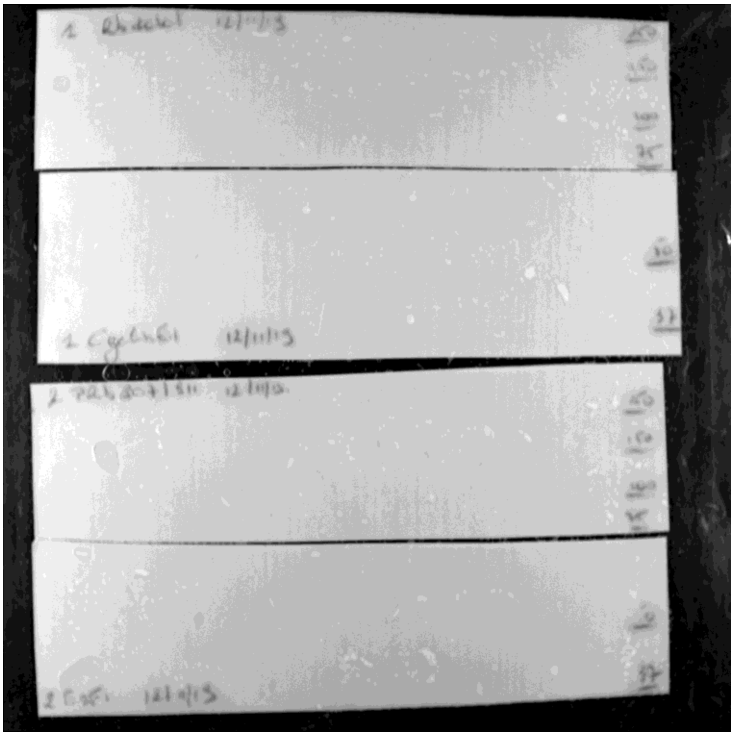

10- TE- exposure 6 sum

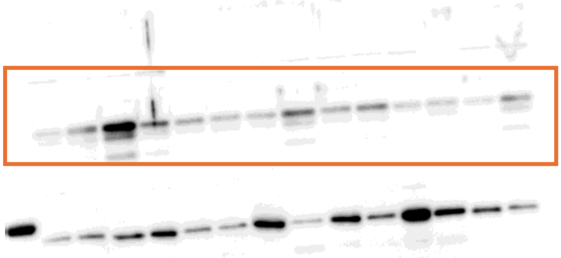

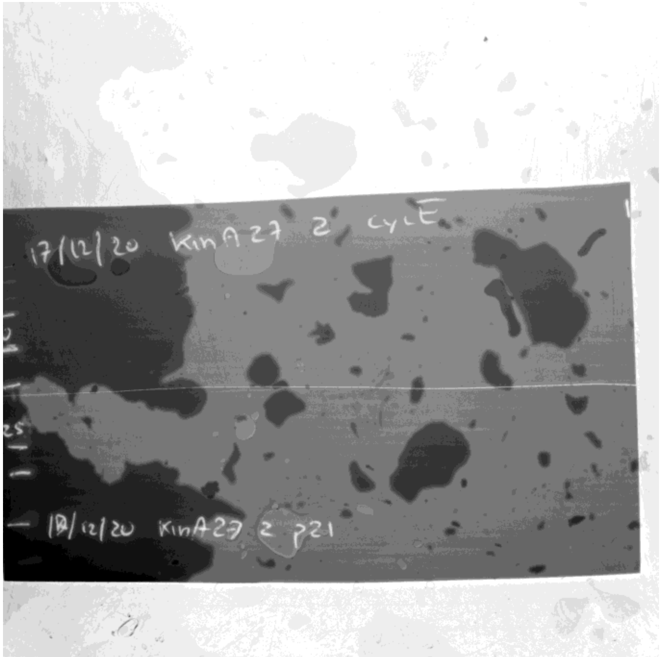

# cyclin A

11- KYSE - exposure 8sum

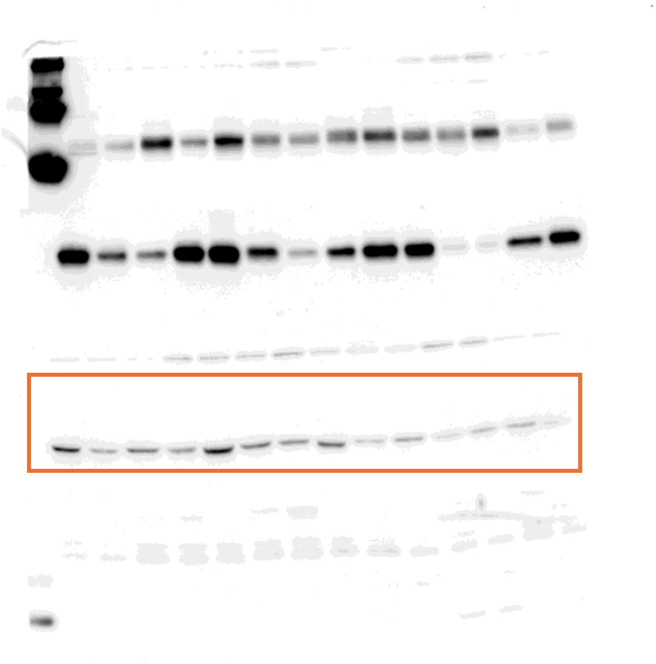

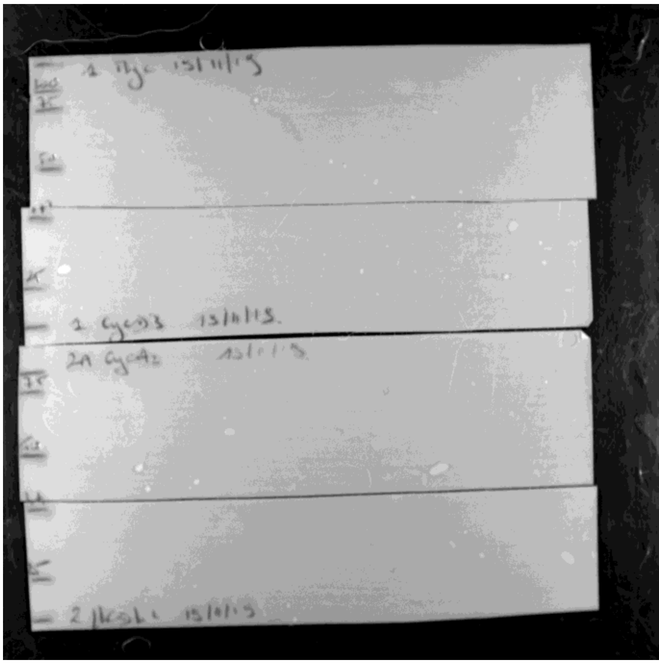

12- TE- exposure 8

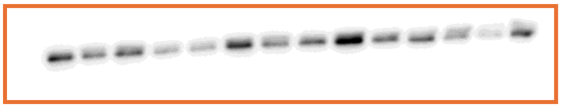

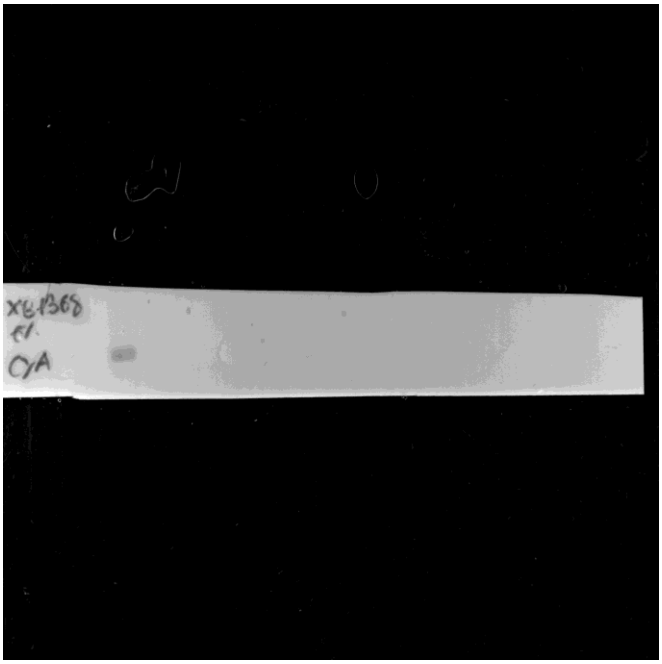

# cyclin B1

13- KYSE- exposure 4'sum

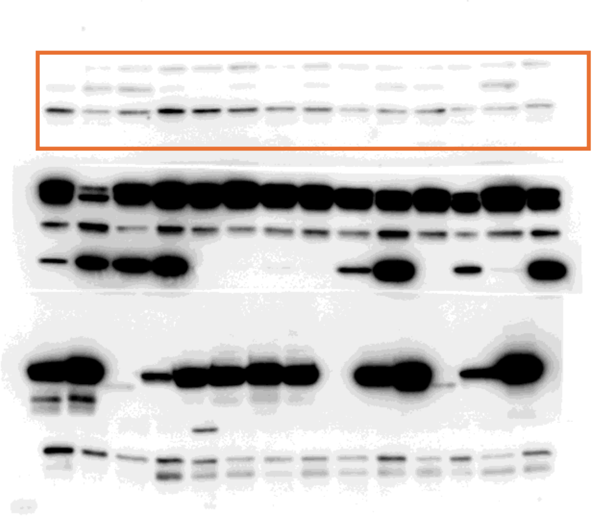

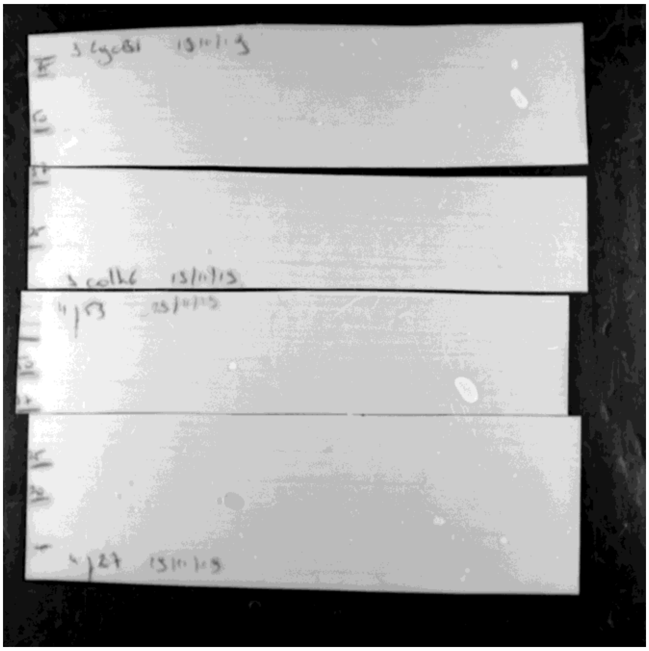

14- TE- exposure 6sum

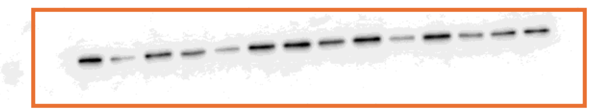

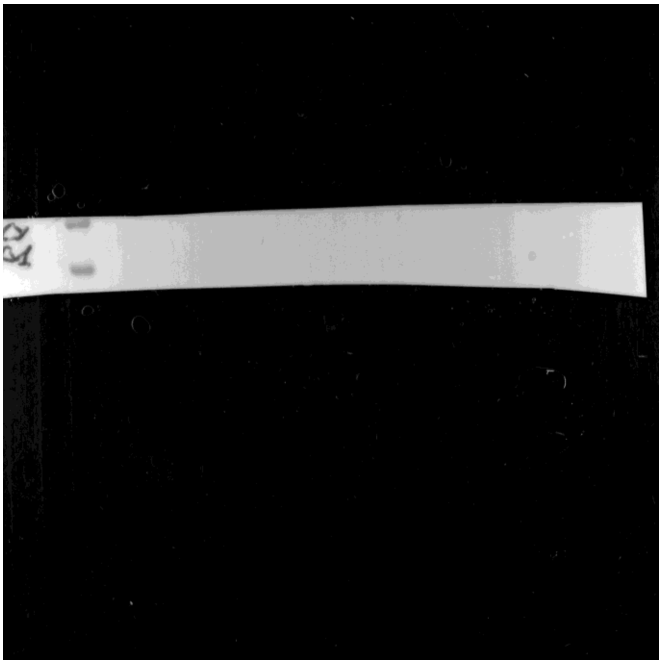

# CDK4

15 – KYSE exposure 6'Sum

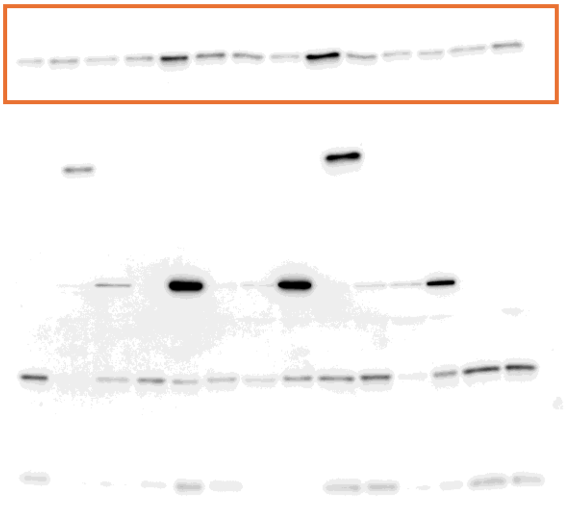

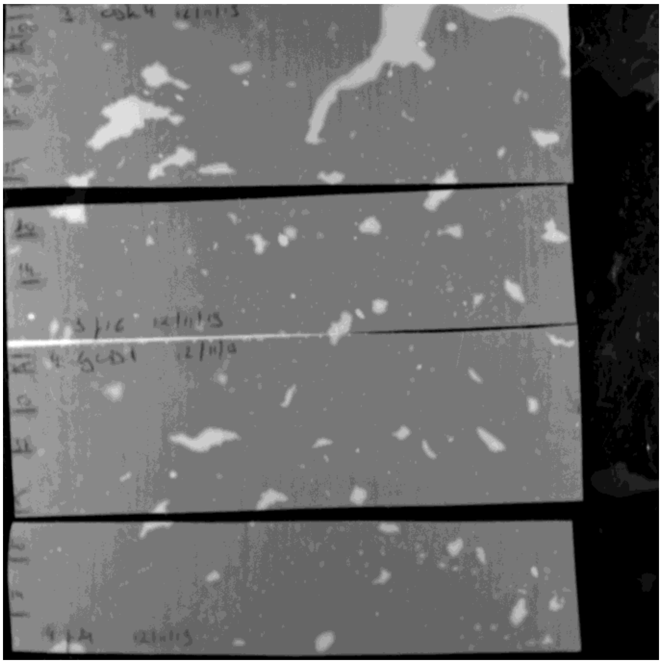

16- TE- exposure - 6

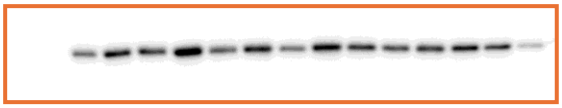

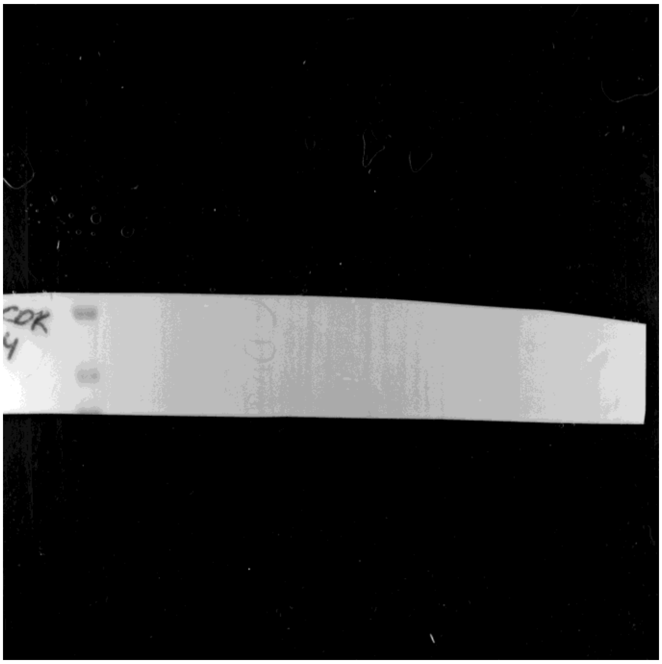

# CDK6

17- KYSE exposure 6Sum

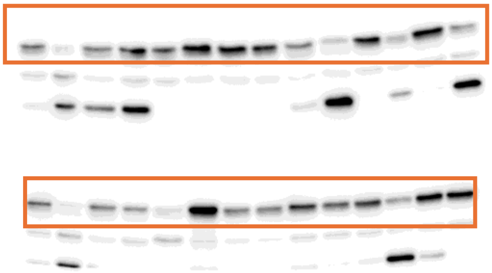

18- TE- exposure 6sum

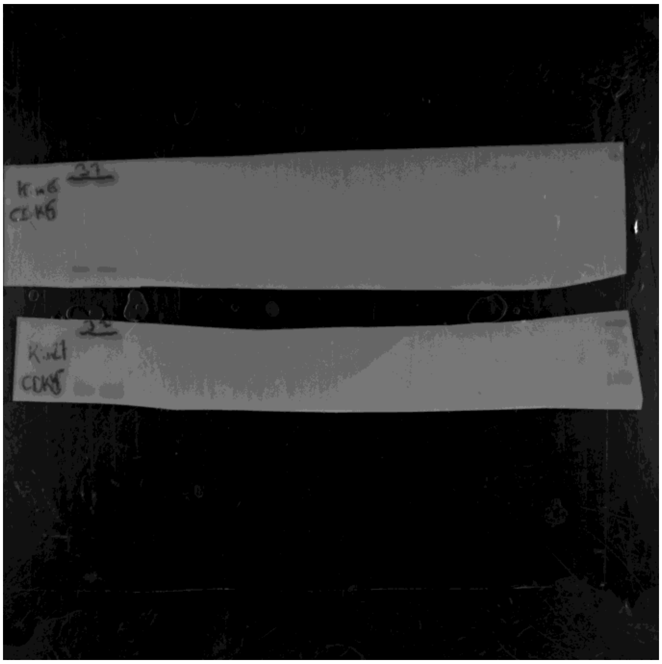

# CDK2

19- KYSE exposure 6sum

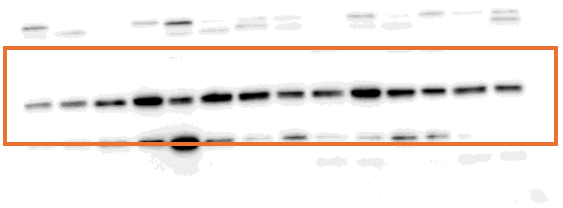

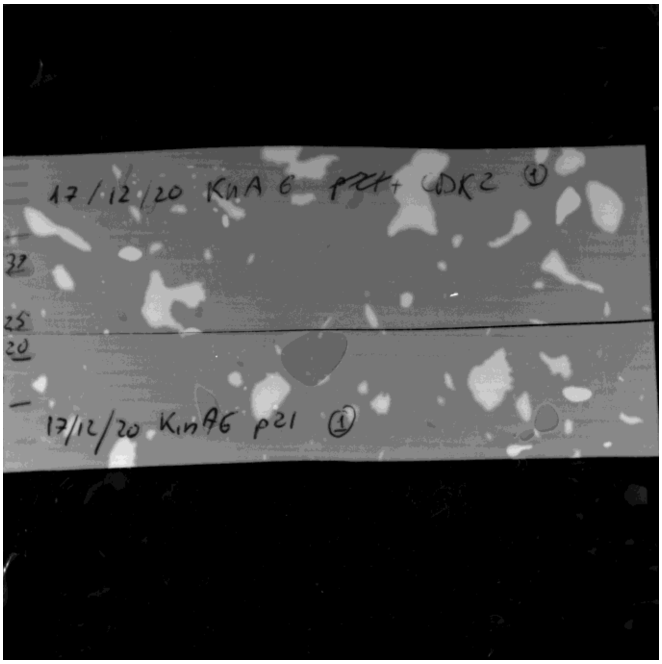


20- TE-  exposure 6Sum

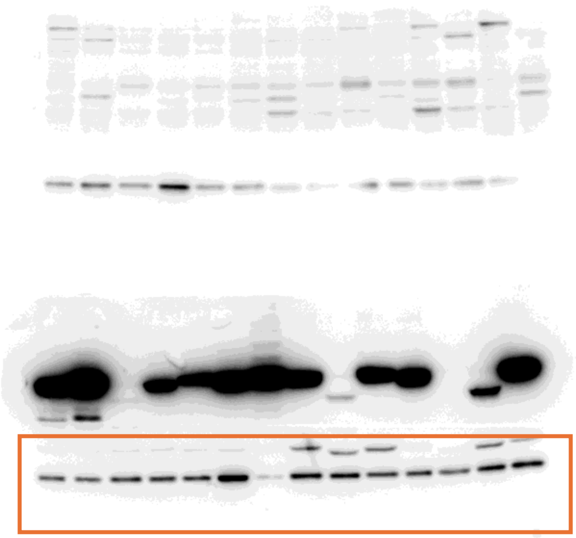

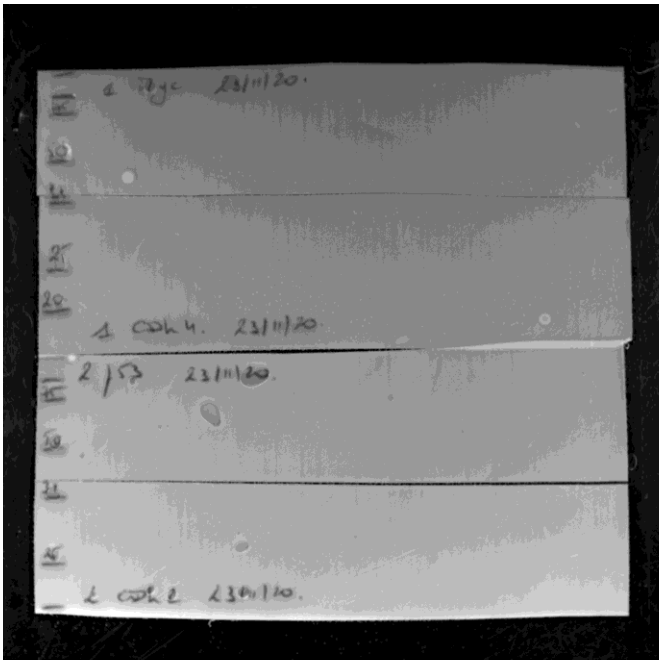

# p16

21 – KYSE - exposure 6'Sum

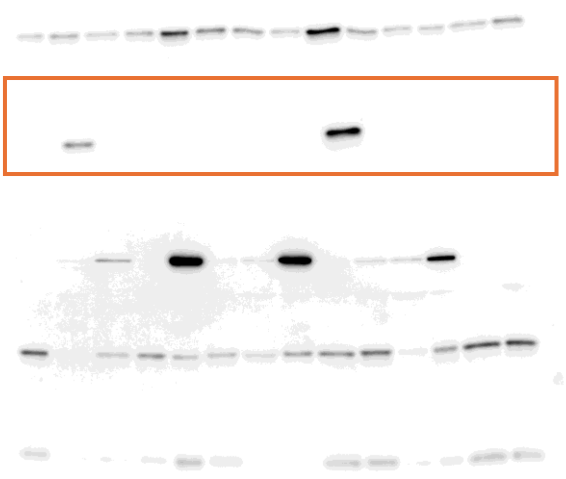

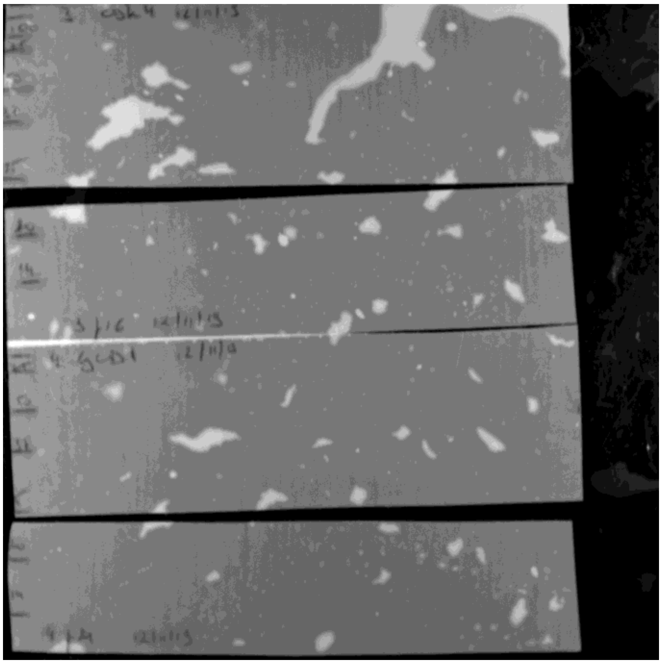

22- TE- exposure 8sum

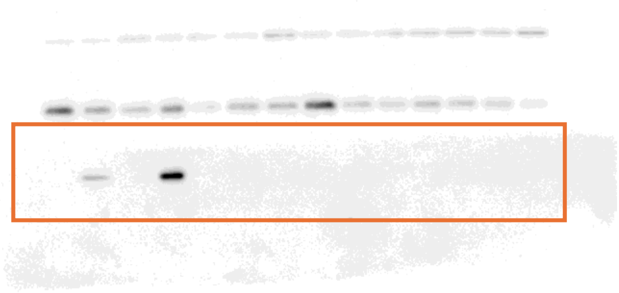

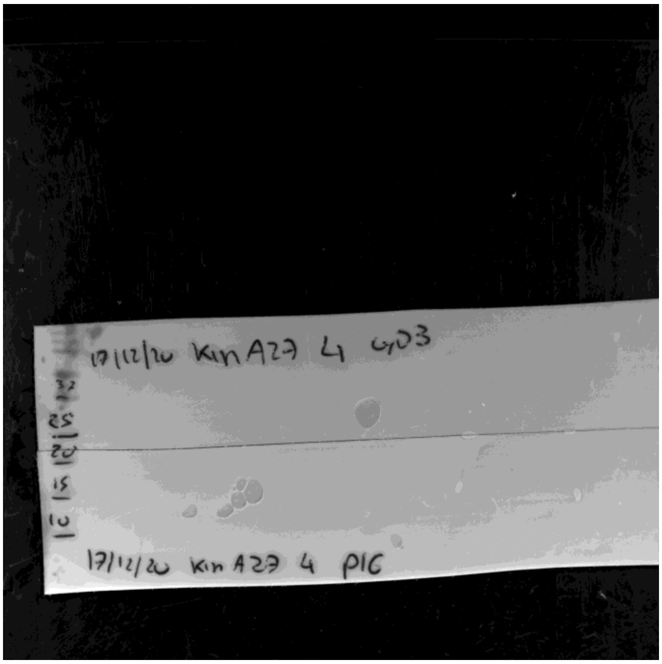

# p53

XB_1084 ID p53 30''

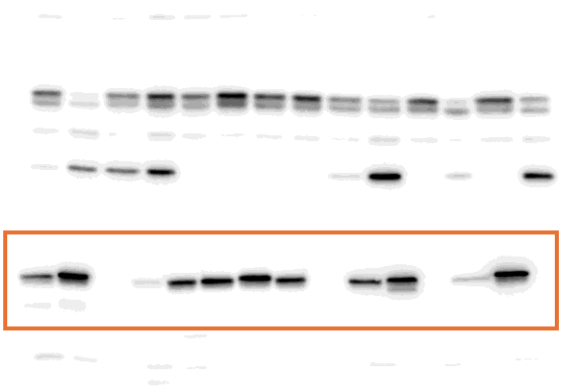

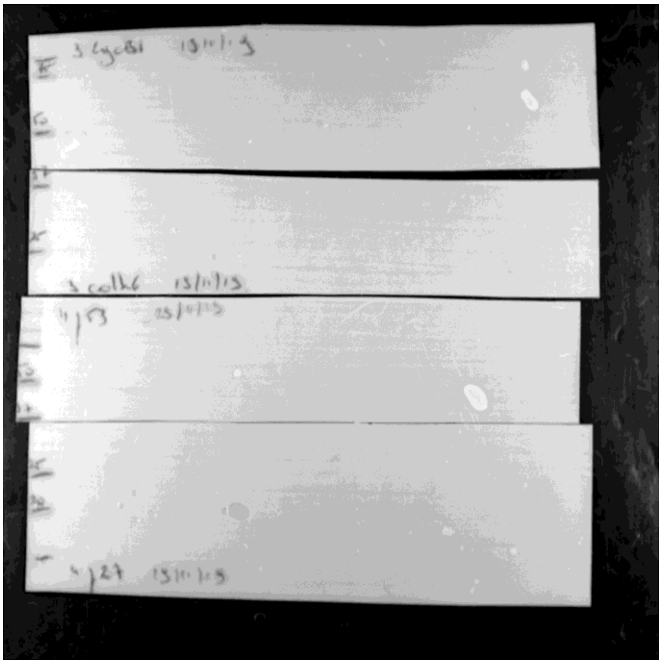

XB_1161 ID p53 73um

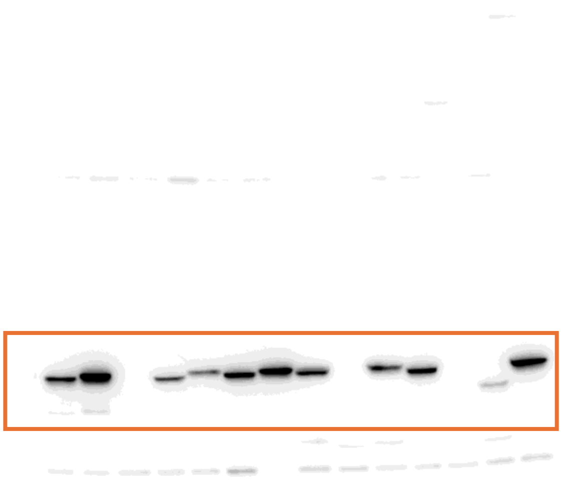

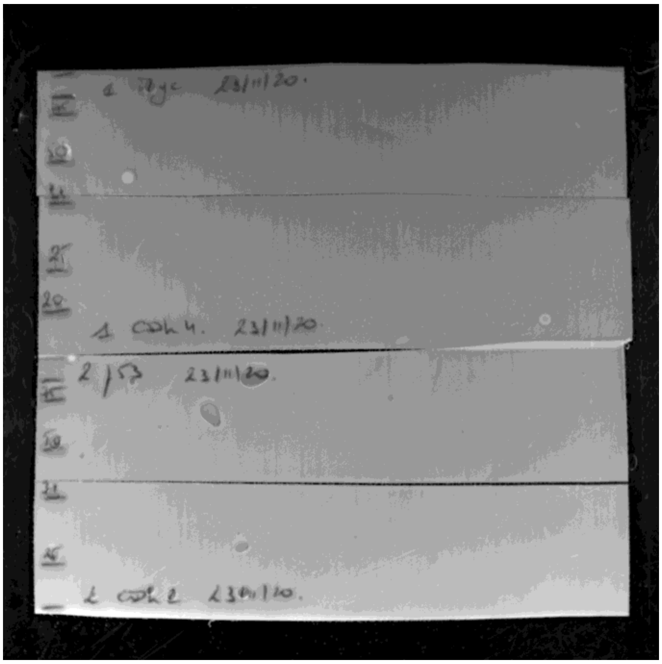

# p63

23- KYSE- exposure 4

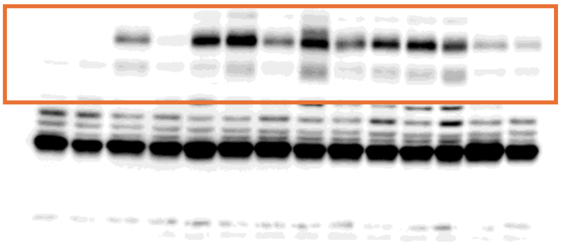

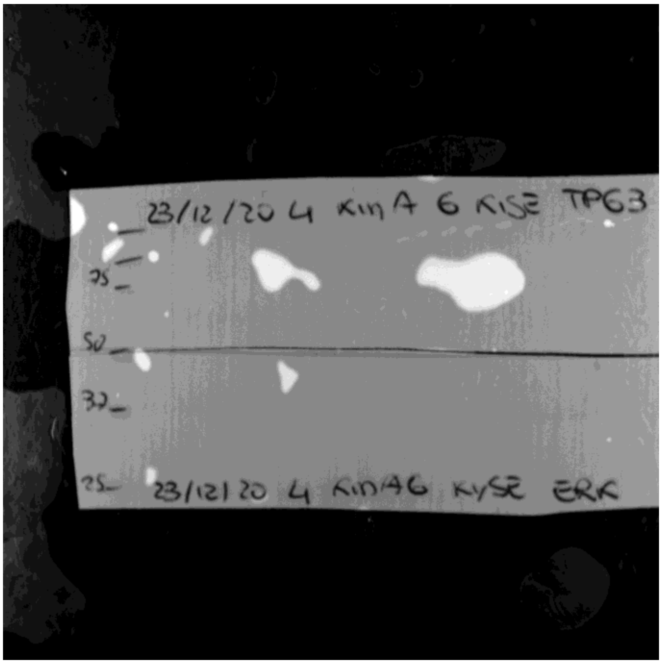


24- TE- exposure 6sum

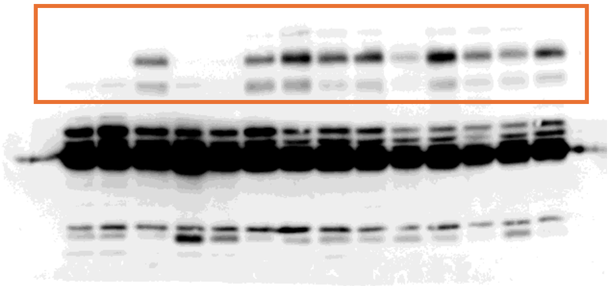

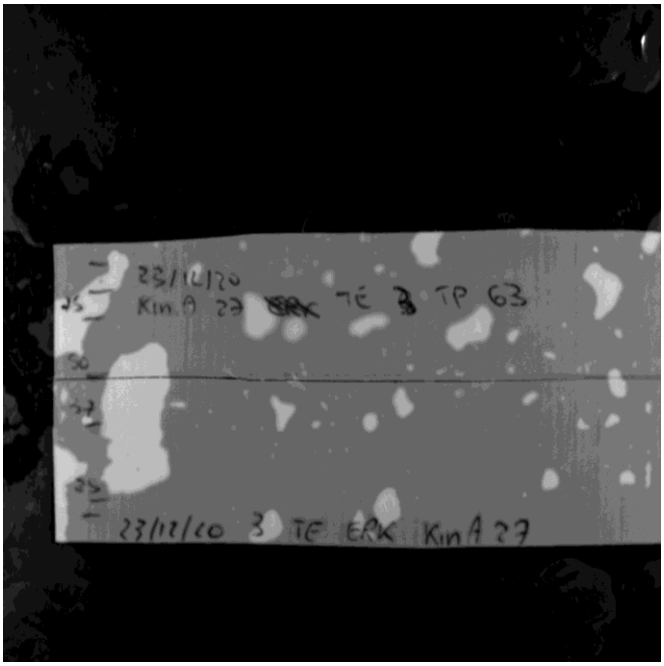

# HSP90

25- KYSE- exposure 2Sum

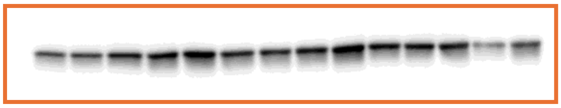

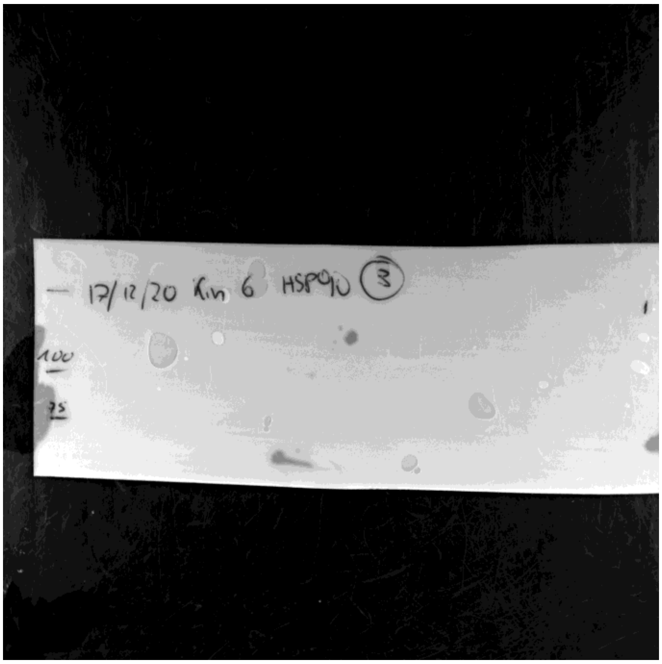


26- TE- exposure 2sum

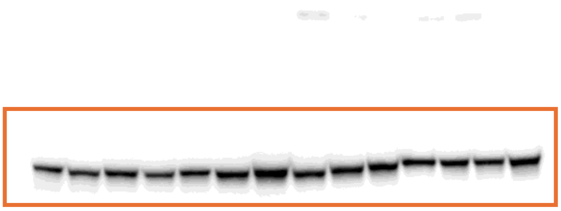

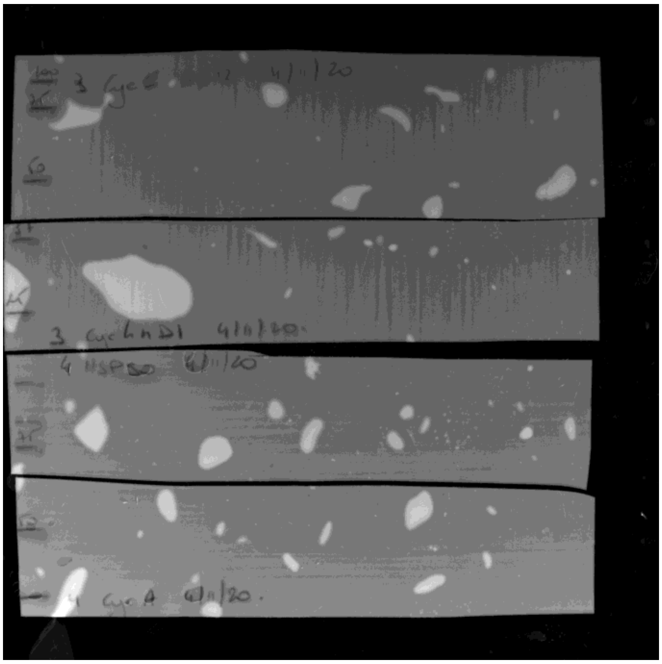

Supplement: Supplementary file 1 — Supplementary Figure and Tables Legends [file 41419_2026_8892_MOESM1_ESM.pdf]
